# Development, implementation and evaluation of an evidence-based program for introduction of new health technologies and clinical practices in a local healthcare setting

**DOI:** 10.1186/s12913-015-1178-4

**Published:** 2015-12-28

**Authors:** Claire Harris, Marie Garrubba, Kelly Allen, Richard King, Cate Kelly, Malar Thiagarajan, Beverley Castleman, Wayne Ramsey, Dina Farjou

**Affiliations:** School of Public Health and Preventive Medicine, Monash University, Victoria, Australia; Centre for Clinical Effectiveness, Monash Health, Victoria, Australia; Medicine Program, Monash Health, Victoria, Australia; Medical Services, Alfred Health, Victoria, Australia; Legal Office, Monash Health, Victoria, Australia; Consumer Representative, Monash Health, Victoria, Australia; Medical Services and Quality, Monash Health, Victoria, Australia

**Keywords:** Health technology, HTA, TCP, Investment, Resource allocation, Decision-making, Implementation, Health services research

## Abstract

**Background:**

This paper reports the process of establishing a transparent, accountable, evidence-based program for introduction of new technologies and clinical practices (TCPs) in a large Australian healthcare network. Many countries have robust evidence-based processes for assessment of new TCPs at national level. However many decisions are made by local health services where the resources and expertise to undertake health technology assessment (HTA) are limited and a lack of structure, process and transparency has been reported.

**Methods:**

An evidence-based model for process change was used to establish the program. Evidence from research and local data, experience of health service staff and consumer perspectives were incorporated at each of four steps: identifying the need for change, developing a proposal, implementation and evaluation. Checklists assessing characteristics of success, factors for sustainability and barriers and enablers were applied and implementation strategies were based on these findings. Quantitative and qualitative methods were used for process and outcome evaluation. An action research approach underpinned ongoing refinement to systems, processes and resources.

**Results:**

A Best Practice Guide developed from the literature and stakeholder consultation identified seven program components: Governance, Decision-Making, Application Process, Monitoring and Reporting, Resources, Administration, and Evaluation and Quality Improvement. The aims of transparency and accountability were achieved. The processes are explicit, decisions published, outcomes recorded and activities reported. The aim of ascertaining rigorous evidence-based information for decision-making was not achieved in all cases. Applicants proposing new TCPs provided the evidence from research literature and local data however the information was often incorrect or inadequate, overestimating benefits and underestimating costs. Due to these limitations the initial application process was replaced by an Expression of Interest from applicants followed by a rigorous HTA by independent in-house experts.

**Conclusion:**

The program is generalisable to most health care organisations. With one exception, the components would be achievable with minimal additional resources; the lack of skills and resources required for HTA will limit effective application in many settings. A toolkit containing details of the processes and sample materials is provided to facilitate replication or local adaptation by those wishing to establish a similar program.

**Electronic supplementary material:**

The online version of this article (doi:10.1186/s12913-015-1178-4) contains supplementary material, which is available to authorized users.

## Background

New health technologies and clinical practices (TCPs) are defined as therapeutic interventions or diagnostic procedures that are considered by a reasonable body of clinical opinion to be significantly different from existing clinical practice. Therapeutic interventions include prostheses, implantable devices, vaccines, pharmaceuticals and medical, surgical or other clinical procedures [1].

Australia has robust evidence-based processes for assessment of new health technologies, clinical practices and medications through the national Medical Services Advisory Committee (MSAC) and Pharmaceutical Benefits Advisory Committee (PBAC). Although these processes are rigorous and provide trustworthy information, they do not address all the requirements of healthcare decision-makers. MSAC decisions only cover therapeutic and diagnostic procedures provided by doctors; they do not include activities of nursing and allied health professionals, models of care or service delivery. PBAC decisions only consider pharmaceuticals for community use and do not include some therapeutic agents used solely in the hospital context. Not all topics being considered by decision-makers have been addressed in national recommendations and central agencies cannot take into account local factors such as population needs, organisational priorities, budgets, capacity or capability. Hence many decisions about the use of TCPs have to be made at the state, regional and hospital levels. At national level, evidence-based assessment and development of recommendations for application and funding of new TCPs is enabled by rigorous processes, underpinned by appropriate resources and expertise. However at the local level limitations in processes, resources and expertise means that decision-making is undertaken with varying degrees of rigour, structure and transparency [2–4].

Monash Health (previously Southern Health) is a large health service network providing primary and secondary care in the south east of Melbourne and tertiary and quaternary care in specialist areas across the state of Victoria, Australia. In 2000, Monash Health established the first Technology/Clinical Practice Committee (TCPC) in Victoria to assess TCPs prior to their introduction. Since then, a number of factors have influenced health agencies around the world in how they approach assessment of new TCPs. These global challenges arise from rapid advances in health technologies; consumers’ desires to be well informed and participate in decision-making; imperatives for transparent, accountable and evidence-based decision-making (EBDM); and the need to get best value from finite or decreasing resources.

Although early leaders in this area, the TCPC acknowledged that there were opportunities for improvement. Limitations of the Monash Health system included inadequate transparency and lack of explicit criteria in decision-making, lack of high quality information for decision-making, meetings called at short notice resulting in lack of representative views and inadequate preparation time, lack of awareness of requirements for applications, need for improved accessibility of application materials and limited resources to monitor newly introduced TCPs. The need for more rigorous processes to ensure safe introduction of new TCPs was identified as a priority for the organisation.

This project was undertaken by the Centre for Clinical Effectiveness (CCE), an in-house ‘Evidence Based Practice Hospital Support Unit’ providing expertise in evidence synthesis, implementation and evaluation [5].

### Aims

The aim of the project was to establish a sustainable, transparent, accountable and evidence-based program for introduction of new TCPs in the local healthcare setting.

This paper aims to outline the development, implementation and evaluation of the program.

A toolkit for introduction of new TCPs in hospitals and health care organisations has also been developed which aims to assist health service staff to establish similar programs by providing detailed descriptions of the components, templates of useful documents and links to resources [Additional file 1].

### Research questions

What is best practice for introduction of new TCPs?

How can best practice be implemented most effectively?

What are the outcomes of implementation and the factors for success?

## Methods

### Design

**Fig. 1:**
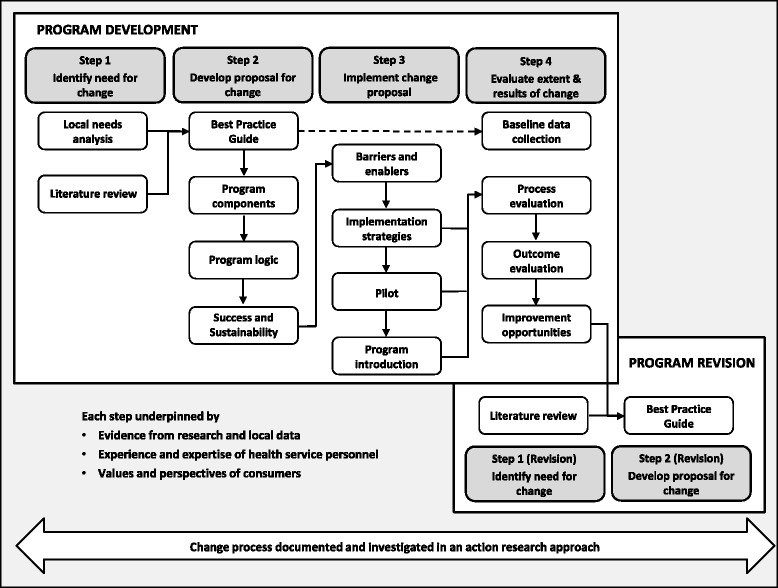
Four step model for evidence-based process change

This project was undertaken using the SEAchange model for sustainable, effective and appropriate change in health services [6]. The model involves four key steps: identifying the need for change, developing a proposal to meet the need, implementing the proposal and evaluating the extent and impact of the change. Each step is underpinned by principles of EBDM to ensure that the best available evidence from research and local data, the experience and expertise of health service staff and the values and perspectives of consumers are taken into account. Factors related to sustainability, avoidance of duplication and integration with existing systems are explicitly considered. Adaptation of the model for this project is outlined in Fig. 1.

Mixed methods were used for process and outcome evaluation. Quantitative methods included audit, surveys and document analysis. Qualitative methods included workshops, individual and group discussions and feedback forms.

An action research approach was adopted based on the ‘researcher as facilitator for change’ model defined by Meyer; researchers working explicitly with and for people rather than undertaking research on them [7, 8]. In this capacity, CCE staff took on the roles of TCPC Executive Officer and Administrative Officer during the development and revision phases of the project. Observations and reflections of the project team and committee members were used for ongoing improvements to the program components and implementation process. Consideration of ‘what worked, what didn’t, why and how it could be improved’ was used throughout.

A timeline for the project is included in the toolkit [Additional file 1].

### Participants

Three stakeholder groups participated in the design, implementation and evaluation of the TCP program.

1. ‘Decision-makers’ were members of the TCPC including an Executive Sponsor; representatives with expertise in operations, finance, evidence-based practice, ethical and legal considerations; clinical program directors and health service consumers. Further details are available in the Terms of Reference [Additional file 1: Appendix 7].
2. ‘Administrators’ were health service staff managing the processes related to making, implementing, monitoring and reporting decisions related to introduction of new TCPs and specialist staff who provided expertise to assist applicants in use of evidence (CCE) and health service utilisation data (Clinical Information Management), coding (Health Information Services), credentialing and scope of practice (Medical Support Unit) and development of business cases (Finance and Business Managers).
3. ‘Applicants’ were clinicians (medical, nursing or allied health) or clinical managers who were seeking authorisation to introduce a new TCP.

### Data collection

Data were collected in an ongoing process over two years and methods were designed to minimise the time and effort required of participants. Scheduled meetings of the TCPC were used for formal workshops and informal group discussions with the decision-makers. Informal interviews with administrators were undertaken during routine meetings or by appointment. The applicants were clinicians based across a number of campuses who found it difficult to attend additional meetings, hence a range of options for individual feedback was provided (details are noted below and examples are provided in Additional file 1).

Discussion papers, background documents and formal presentations were prepared for workshops. All group and individual meetings had an agenda which included the topics for discussion and decisions required.

### Deliberation

Proposals for program design, implementation strategies and evaluation plans were drafted by the project team based on findings from the literature and local research. These were refined based on stakeholder feedback. Decisions were made by the TCPC, discussion was informal and decisions were based on consensus.

### Step 1: Identify the need for change

#### Needs assessment

The views of decision-makers and administrators were sought in group and individual discussions.

To capture feedback from previous and potential applicants, recent users of the existing system were contacted personally and a generic invitation to provide feedback was circulated via the ‘Senior Medical Staff’, ‘All Managers’ and ‘Department Head’ email lists. Email, phone and face-to-face responses were accepted.

Although the four stages in this model are sequential (Fig. 1), change processes are not always linear and often require iterative changes to decisions made in earlier steps. Additional needs were elicited during the implementation and evaluation steps using action research methods including feedback sheets on pilot documents and applicant’s responses to invitations to provide feedback and reflections and observations of the committee and project team.

#### Literature review

A review of international practice to identify key principles for a TCP Program was undertaken. It was anticipated that guidance on development of a systematic approach to governance and decision-making in a health service would be found in policy documents, reports, government publications and research studies. All publication types were eligible and would be included if they addressed methods, processes, recommendations or guidance for introduction of new TCPs.

Finding appropriate search terms for use in health databases was problematic. Broad searches returned too many titles to process. Narrower searches failed to find any relevant literature and it was unclear whether this was due to lack of available information or limitations of the search terms. An internet search was conducted using the search string (new technology clinical practice) AND (committee OR guide OR policy OR procedure) in the Google Advanced Search function.

Critical appraisal relevant to the study design was planned, however no research studies were identified and the expert guidance documents ascertained contained no methods to allow critical appraisal.

### Step 2: Develop a proposal for change

#### Best practice guide

The principles identified in the literature and local needs assessment were collated and tabulated into a Best Practice Guide. Program components were developed through stakeholder consultation and feedback.

#### Likelihood of success and sustainability

A checklist developed for previous CCE projects was used to assess the likelihood of success and sustainability of the proposed changes. The characteristics of success were derived from the work of Grol and Grimshaw; Grol, Wensing and Eccles; Greenhalgh et al. [9–11] and the sustainability factors were adapted from a capacity-building framework [12].

### Step 3: Implement the change

#### Barriers and enablers

Barriers and enablers to the proposed changes were identified by decision-makers and administrators in group and individual discussions, and by applicants in individual discussions, feedback forms at the end of all documents and email invitations to provide input. The project team used the checklist for success and sustainability and the classification of barriers and enablers by Grol and Wensing [13] as prompts to identify additional factors. Barriers and enablers in the context of organisational decision-making were also sought from the literature.

### Step 4: Evaluate the extent and results of the change

#### Evaluation

A formal evaluation plan was developed. Full details including the evaluation questions for each component, indicators, methods, sources and timing of data collection, and reporting schedule are available in the toolkit [Additional file 1].

Current practice was mapped against the identified principles in the Best Practice Guide to provide baseline data. Planned evaluations were undertaken at 12 and 24 months [Additional file 1].

#### Ongoing quality improvement

Following the initial evaluation period, the TCPC and Secretariat continued to collect, analyse and act on feedback as a quality improvement activity.

### Ethics

The Monash Health Human Research and Ethics Committee approved this project as a Quality Assurance activity (Application Number 09195Q).

## Results

### Step 1: Identify the need for change

#### Needs assessment

Twenty-five needs were identified (Table 1). These confirmed the limitations of the existing system and provided opportunities and methods for improvement. Decision-makers noted issues related to meetings held at short notice affecting their ability to attend and adequately appraise materials provided, lack of resources to administer the process and insufficient information on which to base decisions. Applicants reported difficulty accessing information about the process and frustration at being asked to submit applications to multiple committees. Inadequate governance and reporting structures and a need for mechanisms to deal with change of use of TCPs were also noted.

**Table 1:** Needs assessment

Needs	Evidence of need
Identified at initial consultation	
Appropriate and representative views in decision-making	Limited availability of committee members to attend meetings at short notice
Sufficient preparation time for committee members to review applications	Applications provided to committee members 24–48 h before meeting
Increased awareness of requirement for authorisation of new TCPs	New TCPs introduced in the organisation without application or authorisation
More easily accessible application materials	Applicants expressed difficulty accessing application materials
Explicit criteria for decision-making	Lack of documentation for how and why decisions were made
Increased transparency in decision-making process	Lack of documentation of actual decisions
Mechanism to appeal decisions	Applicants are unaware of recourse when they are unhappy with decision
Resources to monitor newly introduced TCPs	Technology/Clinical Practice Committee (TCPC) run by Ethics Committee Secretariat without any additional resources
Reporting of outcomes following introduction of new TCPs	No reporting structure or requirements
Resources to develop, maintain, evaluate and improve rigorous systems and processes	TCPC run by Ethics Committee Secretariat without any additional resources
Electronic communications to reduce inefficiency and inconsistency	All correspondence in hard copy
Identified during program development	
Appropriate categories of information about new TCP provided to decision-makers	Existing application form did not address all principles in Victorian Department of Health guidance
Appropriate detail in information about new TCP provided to decision-makers	Existing application form allowed applicants to determine level of detail provided
Issues of access and equity are considered	Not in previous Monash Health application form or Department of Health guidance
Opportunities for disinvestment of current practice following introduction of new TCP are identified	Not in previous Monash Health application form or Department of Health guidance
Standardised recommendations and conditions to capture and implement decisions	Not in previous Monash Health application form or Department of Health guidance
Increased understanding, skills and resources in evidence based practice	Applications contained inappropriate information to establish evidence of effectiveness
Availability of expertise in assessing costs and health service resource utilisation	Applications contained limited information about costs and resource use
Process to assess when new TCP can be considered ‘standard’ practice, monitoring can be ceased and special patient information is no longer required.	New TCPs are introduced in a ‘probationary’ model. Outcomes are collected and reported and patients are informed that the TCP is new to the organisation and is being monitored.
Process to assess ‘change in use’ of current TCP to identify any potential risks for the patient, clinician and organisation as a result of the change	Current use of TCPs may change to address a new indication or different patient population, if there has been modification to the equipment or technique, or if there are new operators or practitioners.
Process to assess organisational issues (eg capacity, credentialing, funding) for research applications	HREC application process did not address these issues adequately
Process for approval in urgent or emergency situations is in place	Not in previous Monash Health application form or Department of Health guidance
Communication, collaboration and streamlining of processes between the Therapeutics, Technology/Clinical Practice, Human Research Ethics and Clinical Ethics Committees	Applicants submitting to one committee are often asked to submit to a second and sometimes third committee. This results in considerable delays in decision-making and requires additional documentation of the same information on different forms
Patient information sheets are of high quality and consistent with Monash Health patient information format	Brochures submitted by applicants do not meet recognised standards of patient information, do not cover and are not consistent with Monash Health format
Data collection is accurate and produced in a format that can be collated with others for monitoring and reporting	Many clinicians have no knowledge, skills or experience in data collection

#### Literature review

Five relevant publications from national and state bodies were identified [1, 14–17]. The four government agencies and a professional association with expertise in HTA were assessed as appropriate sources for this type of information and the documents were considered to be expert guidance.

The publications identified standards, rules, criteria or principles that they recommended for TCP programs within hospitals or health care organisations. For the purposes of this paper, these are referred to collectively as ‘principles’ for good practice in introduction of TCPs.

Twenty-seven principles were extracted. There was considerable variation in content between the documents with only six principles common to all five publications (Additional file 2).

#### Need for change

The 25 local needs were reframed as principles; four of these had also been identified in the 27 from the literature [1, 14–17], making a total of 48 principles after removal of duplication.

There was a discrepancy in findings between the two sources. Monash Health staff identified five principles related to the need for adequate resources to deliver the program and support applicants in finding and using evidence from research and local data, preparing patient information and collecting and reporting outcomes. These were not identified in the literature.

Monash Health met only 14 out of 48 principles, establishing a clear need for improvement. The 34 unmet principles for good practice indicated the areas to be addressed (Additional file 2).

### Step 2: Develop a proposal for change

#### Best practice guide

‘Best practice’ was defined as implementation of all the principles. Monash Health sought to establish the new program based on this guide to best practice (Additional file 2).

#### Program components

Principles in the Best Practice Guide were discussed with the stakeholders and drafted into the seven components that would form the new TCP Program: Governance, Decision-Making, Application Process, Monitoring and Reporting, Resources, Administration, and Evaluation and Quality Improvement. The aim of transparent, accountable and evidence-based decision-making was made explicit in each of the components. Details of how the principles within each component were operationalised are outlined in the toolkit and copies of all documents and resources are provided [Additional file 1].

#### Program logic

**Fig. 2:**
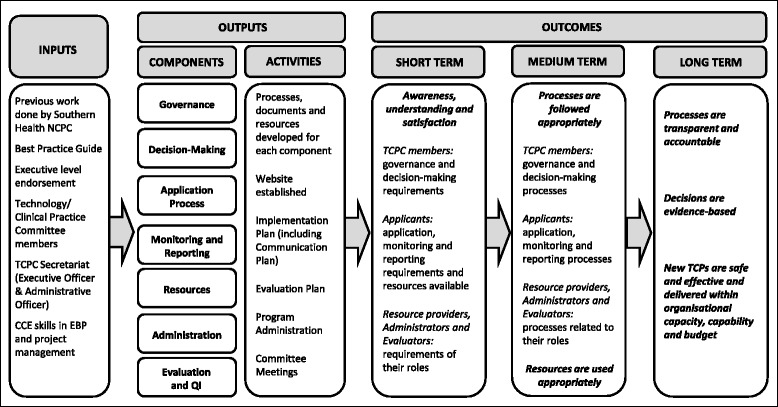
Program logic model for Technology/Clinical Practice Program

A detailed program logic model was constructed incorporating the key factors that required improvement, deliverables identified from the Best Practice Guide, intended outcomes and indicators. A summarised version is presented in Fig. 2.

#### Likelihood of success and sustainability

The proposed program components and logic model were found to meet all the requirements for sustainability; however assessment of likelihood of success identified several potential barriers and enablers (Table 2).

**Table 2:** Assessment of success and sustainability

**Success**	**Met** ^**a**^
A proposal is more likely to be successful if it meets the following criteria	
Based on sound evidence or expert consensus	✓
▪ There is no clear evidence or recognised experts in the area of organisational decision-making for introduction of new TCPs	
▪ However there is general consensus between the guidance documents and the local needs analysis, and no area of disagreement	
Presented by credible organisation	✓DM, ?App
▪ National and state governments and a national professional body are seen as credible in this context by the decision-makers	
▪ It is not known if all applicants will consider them credible as clinicians often see bureaucracy as intrusive and unnecessary	
Able to be tested and adapted	✓✓
▪ A formal pilot will be implemented and evaluated during the state health department funding round for new TCPs	
▪ The whole program will be implemented in ‘pilot mode’, ongoing feedback will be sought and encouraged for the first 2 years	
▪ The project team will adapt the systems, processes and resources based on the stakeholder feedback	
Relative advantage is evident	✓✓DM, ?App
▪ The decision-makers value the benefits in improvements to transparency, accountability and use of evidence	
▪ It is not known if all applicants will consider the changes to be an advantage over the previous system	
Low complexity	╳
▪ The process for introduction of new TCPs is complex and requires time, skills and expertise from the applicants	
▪ The project team have made the application form as user-friendly as possible but it is still detailed and complicated	
Compatible with status quo	✓
▪ There are significant changes for both decision-makers and applicants	
▪ However the changes make the new process very similar to Human Research and Ethics Committee applications	
Attractive and accessible format	╳, ✓
▪ The application form has been made as user-friendly as possible but is unlikely to be considered attractive	
▪ Accessibility has been improved by creating a website as a single point of access for all information, documents and resources	
**Sustainability**	
A proposal is more likely to be sustainable if it has appropriate and adequate provision in each category	
Structure	✓✓
▪ The Technology/Clinical Practice Committee (TCPC) is an appropriate body to manage this process	
▪ Reporting to the Executive Management Team demonstrates that the process has a high priority within the organisation	
▪ The roles of Chair, Executive Officer and Administrative Officer address all aspects of managing the process	
▪ There is appropriate representation on the TCPC	
Skills	✓✓
▪ Members of the TCPC have skills in clinical practice, management, health service operations and finance, ethical and legal issues and evidence-based decision-making	
▪ The Executive Officer and Administrative Officer have skills in managing and administering complex processes	
▪ Staff with skills in finding, appraising and synthesising evidence; coding; analysing health service data; credentialing; business and finance; and infrastructure and equipment needs are available to assist applicants	
Resources	✓✓
▪ Website holds all information centrally	
▪ Expertise is provided as noted above	
▪ Online guidance to completing the evidence components of the application is provided	
▪ Templates are provided to assist applicants and to ensure processes and documents are consistent and of high quality	
▪ Adequate funding has been provided for the TCPC Secretariat to manage all seven components of the TCP Program	
Commitment	✓✓
▪ The health service has demonstrated commitment by making this process an organisational priority	
▪ The Executive Director of Medical Services and Quality and Executive Director of Nursing and Midwifery are both on the TCPC	
▪ All Program/Division Medical Directors, Executive Director of Nursing and General Manager of Allied Health are supportive	
Leadership	✓✓
▪ Members of the previous committee demonstrated leadership in striving to improve the process	
▪ The TCPC is seeking to be a leader in introduction of TCPs by establishing a transparent, accountable, evidence-based process	
▪ The Centre for Clinical Effectiveness is a leader in enabling evidence-based decision-making	

*DM* Decision-makers, *App* Applicants

^**a**^ ✓✓ = Criterion met completely, ✓ = Criterion met partially, ╳ = Criterion not met, ? = Not known

### Step 3: Implement the change

#### Barriers and enablers

Due to the iterative nature of the change process, barriers and enablers were identified prior to, during and subsequent to the implementation phase through the action research reflection and the evaluation activities.

Some factors were not explicitly recorded during the project but were acknowledged implicitly when strategies were developed and implemented to address them. They have been included for completeness and to assist others in replicating this program. Forty-five barriers and ten enablers are reported (Additional file 3).

Barriers were identified in each category and were applicable to decision-makers, administrators and applicants. Key themes are summarised below.

- Economic and political context (*n* = 3): effect of state and national activities related to TCPs
- Organisational context (*n* = 10): lack of time and resources, lack of awareness of current decision-making structures
- Social context (*n* = 5): lack of influence, effect of perceptions and power relationships
- Patient (*n* = 4): need for adequate consumer representation in decision-making, limitations in quality of patient information brochures
- Individual professional (*n* = 17): lack of awareness, lack of knowledge and skills, poor compliance
- Innovation (*n* = 6): complexity, time requirement, perceived lack of advantage

Enablers were related to the organisational commitment to the new program. The project was a high priority and the pursuit of excellence was made explicit. Funding and resources were provided and the Board, Executive, Senior Managers and Clinical Directors were all supportive.

#### Implementation strategies

Implementation strategies were developed to overcome or minimise barriers and build on enablers. Individual strategies are detailed against their corresponding barrier or enabler in Additional file 3. As additional barriers were identified during the course of the project strategies were developed and implemented to deal with them.

The strategies fall into four main groups: changes to the structure of the TCP program, changes to the processes within it, provision of resources and support, and activities to communicate and disseminate information.

The first three groups could also be summarised as ‘make it mandatory, transparent and explicit’, ‘make it as easy as possible to do the right thing and hard to do the wrong thing’ and ‘provide as much help as the organisation can sustain’. These points applied equally to activities of decision-makers, administrators and applicants.

Changes to the structure and processes were implemented, resources were developed and the communication and dissemination strategies carried out. These have all been integrated into the toolkit so that it reflects best practice not only from the literature but also from the extensive learning from this project [Additional file 1].

#### Pilot

The components were piloted during the Department of Health annual funding round for high cost TCPs. Input from decision-makers, administrators and applicants was obtained through invitations to provide phone or email feedback, feedback forms appended to all documents and a meeting held specifically for this purpose. Revisions to the documents and processes were made based on this feedback.

#### Program introduction

The program was implemented in full.

In addition to the specific pilot outlined above all documents were subsequently implemented in ‘pilot’ mode with a feedback section for notes at the end of the documents and an invitation to contact the Secretariat personally with any additional feedback. Further refinements were based on this input and stakeholders were informed. This communicated to applicants that the processes were not rigid, that their feedback was welcome, that it would be acted upon, and that it resulted in improvements.

### Step 4: Evaluate the extent and results of the change

#### Evaluation

Detailed evaluation reports at 12 and 24 months were published on the TCPC website [Additional file 1].

At the commencement of the project, Monash Health initially met only 14 of the 48 principles. At the end of the evaluation period this was repeated and found that all 48 principles had been met (Additional file 2).

When opportunities for improvement were identified by the formal evaluation activities or through the ongoing feedback and action research processes, modifications were implemented to address them. As a result, the program not only achieved all the baseline principles but identified and implemented 51 additional principles across all seven components that were not in the original Best Practice Guide (Additional file 2).

In addition to the formal evaluation questions, other unexpected outcomes indicated the success of the program.

- Recommendations from the Department of Health to other Victorian health services to use Monash Health methods and resources
- Requests from several Victorian and interstate health services for permission to use Monash Health documents
- Request from another state government to provide training in Monash Health methods for their state-wide decision-making body
- Attainment of a national award: Australian Council of Healthcare Standards National Quality Improvement Award for Non-Clinical Service Delivery
- Nomination for a state award: Victorian Public Healthcare Award for Doing It Better: providing sustainable, well managed and efficient health services
- One specific element of the new program, the Joint Committee process, had a successful outcome that enabled an international breakthrough (Table 3: Case study) [18, 19]

**Table 3:** Case study: the Joint Committee process

One particularly significant innovation was the introduction of joint committee meetings for complex applications. Applicants submitting to the TCPC, Human Research Ethics, Clinical Ethics or Therapeutics Committees were often asked to submit to a second and sometimes third committee, depending on the nature, complexity and implications of their application. To reduce the duplication of paperwork, delays in decision-making and wasted time attending multiple meetings a collaborative process involving joint meetings and streamlined documentation was established. Details are outlined in the Toolkit [Additional file 1].
The four committees held their first joint meeting to discuss Baby Z, a neonate with molybdenum cofactor deficiency, a rare metabolic disorder with no effective treatment that leads to death in early infancy. With the permission of Baby Z’s parents, the treating doctors sought authorisation to use a therapy that was effective in mice but had not been tested in humans.
Members of each committee researched in their respective areas (eg scientific literature, legislation and regulations, preparation of therapeutic agents, etc.), provided documentation and contributed relevant expertise in the discussion. A report of the process and compilation of the documentation was undertaken by the Monash Health legal team. This information was used by the Victorian Office of the Public Advocate and the Family Court in their decision to allow the treatment. Baby Z survived [18].
Clearly the scientists and clinicians deserve the credit for identifying and refining this ground-breaking treatment and diagnosing and treating Baby Z. However the decision to use an experimental treatment is a burden that should not be left to the treating clinicians and the family. The rigour of this transparent, accountable and evidence-based decision-making process utilising the specialist knowledge of relevant experts gave those involved confidence that this was the right thing to do. This information was subsequently accepted by other decision-making bodies around the world to expedite rapid treatment of the next few infants diagnosed with the same rare condition. Babies are now routinely treated at birth [19].

The overall conclusion was that Monash Health had met its objectives of achieving a transparent, accountable and evidence-based program for introduction of new TCPs and was consistent with world best practice.

#### Ongoing quality improvement

Although the formal evaluations found that all elements of the program had been successfully implemented, as the outcomes and other implications of newly introduced TCPs were observed over time some shortcomings in the program became apparent (Table 4).

**Table 4:** Opportunities for improvement identified in evaluation (Needs assessment for program revision)

Needs	Evidence of need
Governance	
TCPC members should be of sufficient levels of seniority, credibility and influence to make and implement appropriate and acceptable decisions	Feedback suggested that all Medical Program/Division Directors and General Manager of Allied Health should be on the committee to own and drive decisions within their programs
Expertise on infrastructure and equipment needs, contracts, maintenance, etc. should be available in the TCPC process	Applicants are corresponding with manufacturers/suppliers directly but do not have knowledge of contract negotiation, maintenance requirements, etc.
Decision-making	
Evidence provided should be based on a rigorous systematic review of the research literature	Applicants are not following guidance to undertake systematic reviews properly and not using templates in application form correctly. CCE frequently identifies existing systematic reviews or other evidence that has not been included by applicant.
Application process	
Independent experts should identify the best available evidence from the research literature	▪ Applicant feedback is that they do not have the time, knowledge and skills to do this properly ▪ Applicants do not seek help from experts as required/recommended ▪ Information provided is incomplete, inadequate and/or incorrect ▪ Lack of objectivity results in overestimates of outcomes, underestimates of costs
Independent experts should identify issues relating to resources (financial, space, equipment, staff)	
Expression of Interest form should replace current application form	▪ Applicant feedback is that form is not user friendly ▪ Project team observation is that form is not used correctly
Business Case template should compare new TCP and current practice ‘head-to-head’	Current process uses different methods to assess costs and resource utilisation for new and current
Monitoring and reporting	
Ethics approval as a Quality Assurance activity is obtained prior to data collection	Audits of patient information should be covered by ethics approval
Resources	
Data collection tool and Report proforma for diagnostic tests is available	Current Data collection tool and Report proforma are based on Department of Health requirements and only apply to therapeutic interventions. They do not support reporting of diagnostic tests.
Sufficient staffing levels are provided for expert and independent input to application process	Relevant staff have full workloads and cannot add this unless other work is re-directed

Some of the opportunities for improvement were minor, such as changes to the membership of the TCPC. However after the first 14 application cycles it was clear that some of the original processes were not sufficient to achieve the desired level of rigour in decision-making. Two main issues were identified.

Firstly, the process was ‘applicant-driven’. Applicants were required to provide systematic reviews; as a first step they were to search for existing reviews, if none were available then they were to conduct their own. Lack of knowledge and skills in evidence synthesis had been identified as barriers and strategies to address these included a step-by-step guide to finding, appraising and synthesising research literature [20], templates in the application form to summarise the evidence appropriately and provision of advice from an expert systematic reviewer. A similar approach was advocated for collection and synthesis of local data with prompts for what was required and support from experts in coding, data analysis and finance provided. However applicants did not always follow the instructions in the online guide, did not use the evidence summary tables correctly, in some cases not at all, and many did not follow instructions to consult the expert staff, or consulted but chose to report the information provided selectively. Feedback from applicants themselves and observations of the TCPC and project team confirmed that the resources did not overcome the barriers. An additional challenge is the subjectivity inherent in an ‘applicant-driven’ system where the evidence to inform the decision is provided by those proposing the change.

Secondly, the application form based on the Department of Health requirements did not compare the new TCP with current practice on a ‘head-to-head’ basis. The costs and service utilisation data were gathered and reported in different ways which precluded definitive conclusions based on direct comparisons.

As a result of these two factors, the information provided often had omissions or errors and a tendency to overestimate positive outcomes and underestimate costs.

### Program revision

All stakeholder groups were in agreement that there were problems in providing accurate information for decision-making. The TCPC became aware of models of health service policy committees in Canada and New Zealand that did not rely on applicants to provide information but utilised independent experts within the organisation to investigate the evidence from research and local data and develop a business case for new TCPs. These models demonstrated improved decision-making [Personal communication: Caroline McAleese, Auckland District Health Board] and resulted in considerable cost saving to the organisation [21]. Based on these findings, the TCPC decided to revise the Monash Health program.

### Step 1 (revision): Identify the need for change

#### Needs assessment

The opportunities for improvement identified in Step 4 (Table 4) became the needs assessment for Step 1 of the revision process (Fig. 1). Ten new needs were reported. These reflected the ongoing problems with inadequate and inaccurate information to underpin decisions and the resources required to enable this.

#### Literature review

The previous review process was repeated. The search was augmented with review of reference lists of included publications and website searches of relevant agencies known to the project team.

Sixteen relevant publications from government agencies, professional bodies and health services in Australia, New Zealand, United Kingdom and Canada were identified [1, 14–17, 22–32]. These were considered to be expert guidance from appropriately qualified organisations, no research studies were found.

One hundred and nine principles for a TCP Program were extracted. The additional 82 principles reflect the increased number of publications available and the greater level of detail they recommend.

All 16 publications recommended that a TCPC is established, evidence of safety and effectiveness is robust and reliable, appropriate clinical and physical infrastructure and credentialed and trained staff are in place to support the introduction of new TCPs.

Twenty-six principles were cited by at least two thirds (10 or more) of the publications. These focused on overarching issues relating to governance, use of evidence in decision-making, and application and monitoring processes.

Fifty-eight principles were cited by less than one third (5 or less) of the guidance documents. These fell into two groups: those that specify more detail in the governance, decision-making and application processes and those that address aspects of reporting, administration, provision of resources and support, and evaluation of the program. Only five publications referred to consumer representation and only one suggested reporting outcomes to local consumer health councils or networks [14]. Only one source recommended repeat assessment of a newly introduced TCP at the end of a predefined period to determine whether it could be considered standard practice and monitoring could be ceased [31].

#### Need for change

The 10 local needs were reframed as principles; seven of which were duplicated in the literature. When added to the 109 published principles these brought the total to 112.

The initial literature review did not identify any recommendations for provision of resources, although Monash Health staff had considered this important. The principles related to resource provision from the first needs analysis were now included in the literature, however none of the documents noted principles identified by Monash Health staff for adequate allocation of staff with appropriate skills to manage and support the decision-making process or the need for evaluation and improvement of the systems and processes for introducing new TCPs.

Monash Health met 89 out of 112 principles. Most of the unmet principles related to the need for objective assessments undertaken by independent experts and direct ‘head to head’ comparisons of the TCPs under consideration.

### Step 2 (revision): Develop a proposal for change

#### Best practice guide

The Best Practice Guide was revised to include the additional principles and categorise them into the seven program components. In addition to those above, Monash Health staff had identified and implemented many other principles during the implementation phase, 11 of which were not found in the literature. The recommendation for an Expression of Interest to replace the current application process was not included as it was considered to be a local solution rather than a universal principle for best practice. The final total was 122 principles for good practice (Additional file 2).

#### Amendments to program components

The unmet principles were considered and amendments to the program were proposed. Membership of the TCPC was changed to increase the seniority, credibility and influence of the committee to make and implement appropriate and acceptable decisions. The other amendments focused on improving the quality of information provided to decision-makers, predominantly through the application process.

#### Revised application process

**Fig. 3:**
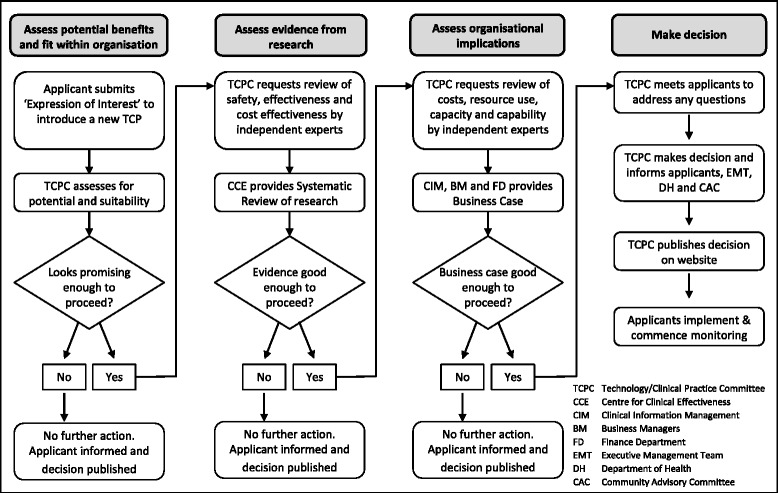
Revised application process for introduction of new TCP

In the new model the previous lack of objectivity, time and skills is addressed by providing resources so that independent experts can undertake the work. To minimise unnecessary resource use, and in contrast to the Canadian and New Zealand models, the information is requested in stages in the Monash Health program, each stage predicated on a positive decision at the stage before (Fig. 3).

Applicants submit an Expression of Interest in a much briefer document than the previous application form which greatly reduces their time commitment [Additional file 1]. The TCPC assesses whether the potential benefits of the new TCP and its fit within the organisation’s goals and priorities is enough to warrant using additional resources to explore it further. If so, the TCPC commissions a Systematic Review of the research evidence by the Centre for Clinical Effectiveness. If there is sufficient evidence of increased safety, effectiveness and/or cost-effectiveness to proceed the TCPC commissions a Business Case. The new Business Case process will address the inadequacies of the previous application form by providing direct comparisons of costs and health service utilisation. This assesses organisational capacity, capability, costs and resource implications and is undertaken by staff with expertise in these areas.

Considering the problems inherent in an ‘applicant-driven’ model and the successes of the international models using independent experts, Monash Health anticipates that use of organisational resources to provide better information to underpin decisions will be cost-effective.

This process is currently being piloted and refined.

## Discussion

### Limitations

A systematic approach was used to ascertain evidence to underpin the new TCP program; however no research was identified for organisational decision-making and clinical governance. Although the documents available were from credible organisations and considered to be ‘expert advice’, no quality appraisal could be undertaken to validate the recommendations. This is not likely to have a significant impact on the process or outcomes of this initiative as the recommendations reflect good practice principles that have been well established elsewhere eg transparent, evidence-based, consumer participation, etc.

### What worked?

The aims of transparency and accountability in decision-making were achieved. The process and requirements are explicit, decisions are published on the internet, outcomes are recorded and activities are reported internally and externally.

Almost all components of the TCP program were implemented effectively. A range of factors are likely to be responsible for this success.

- Use of an evidence-based approach to change guided by information from the literature, local experts and consumers
- Multidisciplinary stakeholder involvement, including consumers, in development, implementation and evaluation of the project and representation in the systems and processes of the ongoing program
- Assessment of barriers and enablers, characteristics of success and factors for sustainability followed by tailoring of strategies to maximise the benefits and minimise the problems identified
- Implementation in a long-term ‘piloting’ mode that captured and acted upon user feedback for continuous improvement
- Credibility of a program underpinned by international best practice
- Provision of sufficient resources to undertake the project and deliver the program
- Commitment, support and leadership from the Board, Executive, Senior Management and Clinical Directors
- Skills of the CCE team in Evidence-Based Practice, knowledge brokerage and implementation of change

The aim of sustainability was also met, at least for the foreseeable future while Monash Health ensures the relevant skills and resources are available to provide high-quality information for decision-making.

It is not uncommon in health services for new initiatives to fail if contextual factors are changed. At the end of the establishment phase the CCE project team handed over to the Medical Governance Office as this was thought to be more suitable and sustainable for ongoing administration of the committee. A second handover to the Director of Medical Services was undertaken and then CCE took over the role again at the beginning of the revision phase. All three handovers went smoothly. The systems, processes, documents and resources proved to be readily transferrable and the program ran seamlessly throughout the moves between departments.

Monash Health demonstrated effective leadership in this area. The high standards achieved by this initiative were acknowledged through a national award and multiple requests to assist decision-makers in other contexts with translation to their settings. Many health services have yet to address the issues related to introduction of new TCPs and it is still common for others to rely on processes that are not evidence-based, transparent or accountable [33].

### What didn’t work?

Minor problems were amended as they arose; these are captured in the needs assessments and barrier analyses and the strategies to address them are summarised in the toolkit. The main areas of concern related to non-compliance with the application requirements which resulted in incorrect or inadequate information being provided for decision-making. This is being addressed in the revision phase.

### Implications for policy and practice

The Australian government has called for reform of post-market surveillance of health technologies to strengthen patient safety and value for money for taxpayers and an international policy forum has proposed that a minimum dataset be developed to focus monitoring activities [34, 35]. This project has demonstrated that it is possible to assess newly introduced TCPs to determine whether practice should continue unchanged, be modified or withdrawn based on locally-collected data. If collection methods are standardised, these data could be pooled at state, national and international level to provide detailed post-market information.

Introduction of mechanisms for prioritisation would improve the program [36]. The current process aims to ensure that a proposed new TCP is safe, effective and can be delivered within organisational capacity and capability. There is no systematic consideration of available alternatives or whether a proposed TCP, even if safe and effective, should be introduced at all. Resources could be saved or redirected to something that has greater impact, is more consistent with organisational priorities or has other benefits.

The structure of the program could be improved by introducing an eighth component for ‘Implementation’. The current Application and Monitoring components include principles that focus on the safety of implementation of a new TCP. Having a specific component would not only capture these but could also include principles that focus on, and highlight the importance of, an evidence-based approach to the implementation process.

The current system is reactive; it responds to individual applications which are driven by non-systematic factors such as clinician’s interests and exposure to promotion of new TCPs. This could be improved through a systematic proactive approach where the organisation seeks out information on new TCPs that are already proven to be safer, more effective or most cost-effective than current practice and considers their fit with organisational objectives and the opportunity costs and risks incurred if they are introduced or not.

There is considerable waste of resources when each health service replicates the information gathering steps to make the same decisions. Sharing of information could reduce this duplication. Monash Health publishes its Decision Summaries on the internet but provision of a central website to house this information at a state or national level might encourage similar publications by others and facilitate access and utilisation for decision-making.

The initial program model was sustainable but proved inadequate to address the aim of robust EBDM. The revised model is likely to meet the aim but, due to the resources required to deliver the evidence, may not be sustainable. Local decisions need to consider local factors however a systematic review of the literature should not be duplicated in each health service. Methods to encourage and facilitate publication of systematic reviews conducted for local decision-making could be explored.

### Implications for research

The lack of research into decision-making processes for introduction of new technologies at the local level and the limitations of ‘knowledge purveyors’ in this context have been noted [33, 37]. This project highlights issues with the quality of information provided by applicants. Lack of knowledge and skills in evidence synthesis were identified as barriers at the outset and are consistent with the findings of others [38]. Resources and tools were developed and expert advice was provided however these initiatives were insufficient to enable applicants to provide trustworthy information. An education program in systematic review methods was not feasible as the potential target audience was too big, any staff member could submit an application. There are systematic reviews on effectiveness of interventions to increase use of research in decision-making [39], education programs for Evidence Based Practice [40] and critical appraisal [41], printed education materials for practice change [42], electronic retrieval of information by health professionals [20] and tailored interventions to overcome barriers [43] but we were unable to find anything on the effectiveness of resources to guide or support clinicians and managers to undertake systematic reviews and health technology assessments for local decision-making. Further research in this area would support efforts to increase the quality of information provided for evidence-based decisions.

Although it is recommended in the Best Practice Guide (Additional file 2) and the potential benefit is acknowledged, most health services do not employ a health economist [44]. However it might be possible for health service staff to incorporate health economic principles in their decisions through application of algorithms or other resources. Research in development and evaluation of tools, templates and guidance materials would facilitate use of health economic methods in local decision-making in the absence of a health economist.

## Conclusion

The Technology/Clinical Practice Program was established using an evidence-based approach to development, implementation and evaluation. The program components were based on a review of the literature, consultation with experts and stakeholders, assessment of characteristics of successful change models and factors for sustainability, identification of barriers and enablers to introduction of best practice and experience from implementation and evaluation in a large health service network.

A toolkit containing details of the processes and resources for implementation is provided to facilitate replication or local adaptation by those wishing to establish a similar program. The components are likely to be generalisable to most health care organisations and, with the exception of the systematic review process, would be achievable with minimal additional resources.

Expertise for effective HTA is not available in most health services, but even if it were, duplication of systematic reviews for the same TCP would be a waste of very limited resources. Some duplication is required for assessment of local factors such as capacity, capability and access but methods to share information that is applicable to all need to be explored.

Further research is required into EBDM for resource allocation at local level.

## Additional files

Additional file 1:
**Harris C, Garrubba M and Allen K.** Introduction of new health technologies and clinical practices: Toolkit for a transparent, accountable, evidence-based program for hospitals and health care organisations. 2014. Technology/Clinical Practice Program. Monash Health, Melbourne, Australia. (PDF 2131 kb) Additional file 2:
**Best Practice Guide.** (PDF 423 kb) Additional file 3:
**Barriers and enablers and strategies to address them.** (PDF 424 kb)

## Abbreviations

CCE: Centre for Clinical Effectiveness
EBDM: Evidence based decision making
HTA: Health technology assessment
MSAC: Medical Services Advisory Committee
PBAC: Pharmaceutical Benefits Advisory Committee
TCP: Technology or clinical practice
TCPC: Technology/Clinical Practice Committee
