# Supplementary material for: Development, implementation and evaluation of an evidence-based program for introduction of new health technologies and clinical practices in a local healthcare setting
Source: BMC Health Serv Res. 2015 Dec 28;15:575. doi: 10.1186/s12913-015-1178-4 (PMC4692064; doi:10.1186/s12913-015-1178-4)
Supplement: Additional file 1: — Harris C, Garrubba M and Allen K. Introduction of new health technologies and clinical practices: Toolkit for a transparent, accountable, evidence-based program for hospitals and health care organisations. 2014. Technology/Clinical Practice Program. Monash Health, Melbourne, Australia. (PDF 2131 kb) [file 12913_2015_1178_MOESM1_ESM.pdf]

Centre for Clinical Effectiveness
Clinicians, Consumers, Evidence

Introduction of new health technologies and clinical practices (TCPs)

Toolkit for a transparent, accountable, evidence-based program for hospitals and health care organisations

2014

Harris C, Garrubba M and Allen K

MonashHealth

Copyright

© This publication is the copyright of Monash Health. Other than for the purposes and subject to the conditions prescribed under the Copyright Act 1968 as amended, no part of this publication may, in any form or by any means (electric, mechanical, microcopying, photocopying, recording or otherwise), be reproduced, stored in a retrieval system or transmitted without prior written permission. Inquiries should be addressed to the Centre for Clinical Effectiveness.

Contact

Centre for Clinical Effectiveness

Monash Health

Phone: +61 3 9594 7581

Fax: +61 3 9594 7554

Email: cce@monashhealth.org

Web: www.monashhealth.org/cce

1. ABOUT THIS TOOLKIT 4
2. ESTABLISHMENT OF THE MONASH HEALTH TECHNOLOGY/CLINICAL PRACTICE PROGRAM 5
3. GOVERNANCE 6
4. DECISION-MAKING 8
5. APPLICATION PROCESS 11
6. MONITORING AND REPORTING 15
7. RESOURCES 17
8. ADMINISTRATION 19
9. EVALUATION AND QUALITY IMPROVEMENT 21
APPENDIX 1. FRAMEWORK FOR EVIDENCE-BASED CHANGE 22
APPENDIX 2: TCPP: TIMELINE FOR DEVELOPMENT OF ORGANISATIONAL APPROACH, SYSTEMS AND PROCESSES 23
APPENDIX 3. BARRIERS AND ENABLERS AND STRATEGIES TO ADDRESS THEM 25
APPENDIX 4. INTRODUCTORY CORRESPONDENCE 29
APPENDIX 5. EVALUATION PLAN 32
APPENDIX 6. POLICY DOCUMENT 34
APPENDIX 7. TECHNOLOGY/CLINICAL PRACTICE COMMITTEE TERMS OF REFERENCE 38
APPENDIX 8. JOINT COMMITTEES TERMS OF REFERENCE 40
APPENDIX 9. DECISION SUMMARY 41
APPENDIX 10: CERTIFICATE OF APPROVAL 44
APPENDIX 11. INTRODUCTION OF A NEW TCP APPLICATION FORM 45
APPENDIX 12. REVISED APPLICATION PROCESS FOR NEW TCPS 61
APPENDIX 13. INTRODUCTION OF A NEW TCP EXPRESSION OF INTEREST FORM 62
APPENDIX 14: REQUEST FOR APPLICATION 65
APPENDIX 15: TWO YEAR REVIEW APPLICATION FORM 66
APPENDIX 16. CHANGE OF USE APPLICATION FORM 68
APPENDIX 17. HREC TCPC APPLICATION FORM 70
APPENDIX 18. FEEDBACK FORM 74
APPENDIX 19. NOTIFICATION OF CONDITIONS 75
APPENDIX 20. LETTER EXPLAINING COMPLIANCE WITH QUALITY ASSURANCE 76
APPENDIX 21. TCP QUALITY ASSURANCE APPLICATION 77
APPENDIX 22. TCP QUALITY ASSURANCE SUPPLEMENT LETTER 79
APPENDIX 23. DATA COLLECTION SPREADSHEET 80
APPENDIX 24. PROGRESS REPORT TEMPLATE 82
APPENDIX 25. CORRESPONDENCE REGARDING REPORTING COMPLIANCE 84
APPENDIX 26: PATIENT INFORMATION BROCHURE 85
APPENDIX 27: AGENDA TEMPLATE 87
APPENDIX 28: MINUTES TEMPLATE 88
APPENDIX 29: REGISTER OF APPLICATIONS 89
APPENDIX 30: REPORTING DATABASE 90
APPENDIX 31: EVALUATION REPORT 2008 91
APPENDIX 32: EVALUATION REPORT 2009 108

1. ABOUT THIS TOOLKIT

CONTEXT

Monash Health (previously Southern Health) is the largest health service network in the state of Victoria providing primary, secondary, tertiary and quaternary services across more than 40 sites including five acute hospitals, subacute and rehabilitation services, mental health and community health services, and residential aged care.

The Centre for Clinical Effectiveness (CCE) is an Evidence Based Practice Hospital Support Unit[1] within Monash Health providing expertise in evidence synthesis, implementation and evaluation. Its role is to enable clinicians, managers and policy makers to use the best available evidence to improve healthcare decision-making.

A new 'technology or clinical practice' (TCP) is a therapeutic intervention or diagnostic procedure that is considered by a reasonable body of clinical opinion to be significantly different from existing practice.[2] Therapeutic interventions include prostheses; implantable devices; high cost pharmaceuticals; medical, surgical or other clinical procedures.

Many health services have a robust application process to ensure that new TCPs are safe, effective and cost-effective. At Monash Health this process is overseen by the Technology/Clinical Practice Committee (TCPC) and managed by CCE. Details can be found at http://www.monashhealth.org/page/Health_Professionals/TCPC/.

This program won the Australian Council of Healthcare Standards Quality Improvement Award for Non-Clinical Service Delivery and was nominated for a Victorian Public Healthcare Award. Monash Health TCPC processes and resources have been implemented by other health services and state health departments. This toolkit has been developed to facilitate sharing of the knowledge and resources acquired in development and refinement of the program.

The Technology/Clinical Practice Program (TCPP) has seven components: Governance, Decision-Making, Application Process, Monitoring and Reporting, Resources, Administration, and Evaluation and Quality Improvement. Each component has multiple elements.

- Many of the elements are straightforward and will be self-evident to those using this toolkit. They are included for completeness and so that others wishing to establish a TCPP do not have to reinvent the wheel.
- Some elements may seem to be straightforward but were not initially self-evident to the project team in the development phase. They are included so that others can avoid the same mistakes.
- Other elements reflect major changes in thinking and practice. They are included to stimulate discussion and change in organisations with established practices in these areas.

PURPOSE

This toolkit aims to help users establish or update their own TCPP by providing

- the best available evidence from the international literature
- samples of resources that can be used in their current format or amended to meet local requirements

AUDIENCE

The intended audience for this toolkit is health service clinicians, managers and policy makers establishing a new TCPP within their organisation or those wishing to improve their current systems and processes. The format and content of the toolkit assumes that users may not have any expertise in establishing programs for the introduction of new technologies or clinical practices.

HOW TO USE THIS TOOLKIT

The chapters in this toolkit are based on the seven program components. Resources are provided in appendices.

Links to resources, documents, templates, etc are shown on the right hand side of the page with ❖

---

[1] Robinson JS, Turnbull DA: Changing healthcare organisations to change clinical performance. MJA 2004, 180(6 Suppl):S61-62

[2] Department of Human Services: Guidance for Victorian Public Health Services to establish Technology/Clinical Practice Committees. 2006, Melbourne Australia.

2. ESTABLISHMENT OF THE MONASH HEALTH TECHNOLOGY/CLINICAL PRACTICE PROGRAM

DEVELOPMENT

The Monash Health Technology/Clinical Practice Program (TCPP) was developed, implemented and evaluated using a rigorous and systematic evidence-based approach which is described elsewhere.[3] This ensured that evidence from research and local data, experience of health service staff and consumer perspectives were incorporated at each of the four steps: identifying the need for change, developing a proposal, implementation and evaluation.

Framework for Evidence Based Change (Appendix 1) ❖

BEST PRACTICE GUIDE

This toolkit is based on evidence from the international literature and the experience of a large Australian health care network. Principles for best practice in introduction of new TCPs were identified from a literature review, local needs analysis with input from decision-makers, administrators and applicants, and feedback during implementation and evaluation of the program at Monash Health. These were collated to form a 'Best Practice Guide'.[3]

The principles from the Best Practice Guide for each component of the program are presented at the beginning of the relevant chapter, followed by details of the Monash Health program and links to available resources.

PROJECT MANAGEMENT

A project timeline was developed based on the four key steps of the evidence-based change process. This was refined when objectives for the change proposal and scope of the project were defined. Staff time allocated to the project was quite limited so the timeline allowed for development and piloting of the new program over 12 months and implementation and evaluation in the second 12 month period.

The timeline is provided as an illustration of the time commitment required and activities involved in establishing a TCPP. The extensive development phase could be reduced by adapting materials provided in this toolkit.

Project Timeline (Appendix 2) ❖

Barriers and enablers to the new program were identified from all stakeholder groups and the research literature. Implementation strategies were developed to overcome or minimise barriers and build on enablers.

Barriers, enablers and implementation strategies (Appendix 3) ❖

The Communication Plan included a range of correspondence and dissemination strategies. To introduce the new TCPC process, personalised letters were sent to Department Heads and Unit Managers, a brief was sent to the Executive Management Team and the Monash Health Board, and a generic letter was sent to the 'All Managers' and 'Senior Medical Staff' email lists. Specific elements of the TCPC process such as reporting requirements, evaluation process, Change of Use and Two year review were sent to Department Heads and Unit Managers.

Introductory Correspondence (Appendix 4) ❖

A formal Evaluation Plan was developed and included evaluation questions for each component; indicators; methods, sources and timing of data collection; and the reporting schedule.

Evaluation Plan (Appendix 5) ❖

---

[3] Harris C, Turner T, Wilkinson F. SEAchange: Guide to a pragmatic evidence-based approach to Sustainable, Effective and Appropriate change in health services. 2015. Available from: http://arrow.monash.edu.au/hdl/1959.1/1225377. Accessed: November 2015

3. GOVERNANCE

Best Practice Guide

- Definition of new technologies and clinical practices (TCPs) is provided
- Organisational policy on 'Introduction of new TCPs' is available
- Responsibility for management, administration and review of policy on 'Introduction of new TCPs' is stated
- Organisational policy states that new TCPs cannot be introduced without approval
- Compliance with organisational policy on 'Introduction of new TCPs' is mandatory
- Advice on whether TCP falls within the scope of 'Introduction of new TCPs' policy is provided
- A Technology/Clinical Practice Committee (TCPC) is established
- TCPC members have sufficient levels of seniority, credibility and influence to make and implement appropriate and acceptable decisions
- There is a range of clinical disciplines represented on the TCPC
- There is a consumer representative on the TCPC
- There is expertise in Evidence Based Practice, Corporate Operations, Finance, Infrastructure and Equipment needs, Ethics and Legal issues on the TCPC
- Additional members can be co-opted to the TCPC for expertise, independence, etc as required
- External/independent expertise is available for advice to the decision-making committee
- The TCPC operates within a reporting structure to ensure corporate and clinical governance
- TCPC meetings are held at regular intervals
- Meeting dates are scheduled in advance and published
- A clear process for appeal is in place
- Any conflicts of interests are disclosed
- Manufacturers, vendors and suppliers are not permitted to submit a TCP application
- Risk management procedures are in place
- Review of complex applications is facilitated by communication with other relevant committees (eg Human Research Ethics, Clinical Ethics, etc)
- Sufficient staffing levels are provided to administer the Technology/Clinical Practice Program
- Sufficient staffing levels are provided for expert and independent input to application process

Monash Health Program

Definition

New health technologies and clinical practices (TCPs) are defined as therapeutic interventions (including prostheses, implantable devices; high cost pharmaceuticals; medical, surgical or other clinical procedures) or diagnostic procedures that are considered by a reasonable body of clinical opinion to be significantly different from existing clinical practice.

Policy

- An organisational policy regarding introduction of new TCPs is in place.
- All new TCPs must be approved prior to introduction
- The policy is mandatory
- The policy is underpinned by a protocol

'Monash Health will ensure that any new health technologies or clinical practices which are introduced are supported by evidence of appropriateness, safety, clinical effectiveness and are financially sustainable.'

'This policy applies to any technology or clinical practice that is proposed to be used for the first time at Monash Health including situations where new devices are provided by manufacturers without charge. It covers change of use of a currently approved technology or clinical practice. It also applies to research projects which involve a technology or clinical practice which is new to Monash Health.'

Policy and Protocol (Appendix 6) ❖

TCPC Terms of Reference

The Terms of Reference for the Committee are documented explicitly and include Definitions, Membership, Roles and Responsibilities, Reporting processes, Quorum requirements, Business rules, Meeting schedule, Appeals process and Quality assurance activities.

TCPC Terms of Reference (Appendix 7) ❖

Scope

The TCPC addresses applications for new TCPs that are proven to be safe and effective to be introduced for all eligible patients within the health service.

New TCPs that are not proven to be safe and effective are addressed by other committees, with input from the TCPC as required.

- New TCPs that are not proven to be safe and effective may be introduced within research projects. The appropriate application process must be undertaken with the Human Research Ethics Committee (HREC).
- New TCPs that are not proven to be safe and effective may be considered for individual patients in extenuating circumstances who give informed consent (eg terminal illness, failure of other treatment, etc). The appropriate application process must be undertaken with the Clinical Ethics Committee (CEC).

If, in case of a genuine emergency, approval is required for immediate use of a new clinical practice, including a new or new use of a device, 'one use only' approval may be given by either the Chair of the TCPC or the Chief Medical Officer.

Conflict of Interest

- Declaration of Conflict of Interest is required from
  - Applicants via a section in the application form
  - Decision-makers via a standing item on the meeting agenda
- Manufacturers, vendors and suppliers are not permitted to submit a TCP application

Joint committee meetings for complex applications

Some applications require authorisation from more than one committee due to the nature, complexity and implications of the new TCP eg Therapeutics, Technology/Clinical Practice, Human Research Ethics and Clinical Ethics Committees. Applicants submitting to any one of these committees were often asked to submit to a second and sometimes third committee.

Monash Health introduced joint committee meetings and streamlined documentation for complex applications to reduce the delays in decision-making, duplication of applications, and wasted time attending multiple meetings. When two committees are involved, one is identified as the primary committee and invites the Chair, Executive Officer and another representative of the secondary committee to attend their routine meeting. When several committees are involved, the Chair, Executive Officer and another representative from each committee attends a specifically convened meeting.

A simplified TCPC application was also developed for HREC applicants implementing a new TCP in their research project.

Joint Committees Terms of Reference (Appendix 8) ❖

Sufficient staffing levels to support TCPP

- Secretariat consisting of Executive Officer and Administrative Officer. These two roles could be done by the same person or be divided among more people depending on local needs and priorities.
- Expert staff to provide information for decision-making including evidence from research literature, coding, credentialing and scope of practice, bed utilisation, current and proposed financial impact, business case, and infrastructure and equipment needs.

4. DECISION-MAKING

BEST PRACTICE GUIDE

- There are established principles for assessment of applications to introduce a new TCP
- The new TCP has been evaluated or used elsewhere
- Evidence concerning a new TCP is robust and reliable
- Safety of a new TCP is established
- High level evidence is required if the application is based on a case for increased effectiveness (eg Systematic Review, RCT)
- Any available evidence of cost-effectiveness of a new TCP is provided
- Health economics approach is included eg considering opportunity costs, indirect and direct costs and benefits, etc
- Issues of access and equity are considered
- Ethics procedures are in place to protect patients, clinicians and the community
- Legislative requirements are met
- Regulatory approval is required
- Standards of practice set by professional associations are met
- Patient information and informed consent procedures are established
- Recommendations for introduction have clearly noted conditions eg audit, clinical trial, operational restrictions
- Decisions of the committee are published to ensure transparency and accountability
- Newly introduced TCPs are reassessed at the end of a predetermined monitoring period to reclassify as 'standard care'
- Approval required for 'change of use' of current TCP (eg new indication/population/practitioners or modification to equipment/technique)
- Approval required, in addition to Human Research Ethics Committee authorisation, for introduction of a new TCP in a research project
- A clear process for handling urgent introductions of new TCPs to minimise patient harm is in place

MONASH HEALTH PROGRAM

Overview

- Decisions are evidence-driven, requiring high level evidence from research and good quality local data
- Decisions are consistent, based on standardised sets of criteria for making decisions, recommendations for documenting decisions and conditions for implementing decisions
- Decisions are transparent and accountable through publication of Decision Summaries which detail the information underpinning decisions and the outcomes of the decision-making process
- Decisions are required in five settings: introduction of new TCPs, reclassification of newly introduced TCPs as standard practice after a two year monitoring period, change of use of TCPs in current practice, introduction of a new TCP in a research project, and use of a new TCP in an emergency situation
- Patient information brochure is provided for the new TCP to enable patients to give informed consent

Introduction of new TCP

This process is for introduction of a TCP that has not previously been undertaken within the organisation.

Standardised criteria for making decisions

- Increased safety, clinical effectiveness and/or cost effectiveness are demonstrated by valid and reliable evidence. Minimum requirements are
  - Safety: Appropriate cohort of sufficient size
  - Clinical effectiveness of therapeutic interventions: At least one randomised controlled trial
  - Clinical effectiveness of diagnostic tests: measures of diagnostic accuracy (sensitivity, specificity, positive and negative predictive value) from appropriate studies
  - Cost effectiveness: Published studies or good quality analysis of local data
- Cost, affordability and source of funding are appropriate
- Organisational capacity, capability, training and credentialing are adequate

- Access, equity and ethics are addressed
- Legislation, regulations and standards are met
- Patient information brochure for the new TCP is sufficient to enable informed consent

Standardised recommendations for documenting decisions

- Recommended: Approved with no further need for assessment
- Restricted Recommendation: Audit (6 monthly reports of routine data for 2 years, immediate reports of adverse events)
- Restricted Recommendation: Clinical Trial
- Restricted Recommendation: Operational Restrictions (eg subject to successful external funding application)
- Not Recommended

Standardised conditions for implementing decisions

- Head of Department/Unit is required to notify the Secretariat of TCPC in the event of:
  - any change in protocol, the reason for the change and an indication of any ethical implications
  - adverse events related to the TCP and steps to deal with them
  - any unforeseen events
- If an adverse event occurs the Head of Department/Unit must immediately notify the Australian Therapeutic Goods Administration (TGA) in addition to the TCPC.
- Data are to be collected on all patients receiving the new TCP and reports provided to TCPC. The TCPC will provide details of data required by the state health department.
- Applicants are required to complete a Quality Assurance application for clinical audit and forward to Monash Health HREC prior to commencement of TCP.
- Reporting is required at six monthly intervals (Jan-Jun and Jul-Dec) for two years post-introduction. Reports to be forwarded to TCPC Secretariat. TCPC to forward reports to the state health department.
- At the conclusion of the two year monitoring period the application will be reviewed by the TCPC to determine if it should be considered standard practice.

Special conditions for implementing decisions

- As required, for example Introduction of <new TCP> is contingent on <specified training requirements, successful funding application, etc>.

Two year review for reclassification of newly introduced TCP as standard practice

This process is to determine whether a recently introduced TCP can be reclassified as standard practice or if it requires further monitoring and reporting.

Standardised criteria for making decisions

Changes to conflict of interest status, changes to use of TCP, new evidence in published literature, comparison of local outcomes with published data, comparison of actual versus anticipated local outcomes of TCP, assessment of ongoing costs and resource use, changes to department/unit procedures, changes to staff training and credentialing requirements, amendment of patient information, any internal reviews eg Clinical Risk Panel.

Standardised recommendations for documenting decisions

- Approved as standard practice
- Approved with conditions for continued monitoring (to be specified)
- Not approved for continued use

Change of use of existing TCP

This process is to inform the TCPC of any changes in use of current TCPs and identify potential risks to the patient, clinician and/or organisation as a result. Change of use can include a new indication for use with the current patient group, new patient group, modification of equipment, new operators or practitioners, or other change.

Standardised criteria for making decisions

Conflict of interest, potential harm to patients, establishment costs, increased resource use/ongoing costs, impact on other clinical disciplines or services, requirement of new code, change to department/unit procedure list, staff training

and credentialing, staff practice change, change to patient access, ethical issues, legislative or regulatory requirements, radiation source, amendment of patient information materials, risk to patients/staff/organisation.

Standardised recommendations for documenting decisions

- Approved
- Approved with conditions (to be specified)
- Not approved

HREC application entailing a procedure or clinical practice new to the organisation

This process is to inform HREC and TCPC of any financial, operational and/or credentialing requirements arising from the use of a new TCP in the context of a research trial and to identify potential risks to the patient, clinician and/or organisation.

Standardised criteria for making decisions

- Conflict of interest
- Impact on patients: potential harm, comparison with current practice, arrangements for patients readmitted after undergoing TCP, effect on hospital demand management, additional considerations
- Financial and operational implications: establishment costs, increased resource use or ongoing costs, impact on length of stay and funding mechanism if this is expected to increase, number of patients being treated and funding mechanism if this is expected to increase
- Impact on organisation: impact on other clinical disciplines or services, TGA approval details if TCP is prosthesis, device or drug, legislative or regulatory requirements, radiation source, risk to staff or organisation
- Credentialing and scope of practice requirements

Standardised recommendations for documenting decisions

- Approved
- Approved with conditions (to be specified)
- Not approved

Use of new TCP in urgent or emergency situations

This process is for use of a new TCP or new use of a TCP in current practice in an urgent or emergency situation to minimise patient harm when there is insufficient time to follow the standard application process.

Standardised criteria for making decisions

- Benefits outweigh risks
- Patient provides informed consent where possible

Standardised recommendations for documenting decisions

- Approved
- Not approved

Decision Summaries

- Documents summarising the findings for each decision criteria, the committee recommendations and any conditions that were applied
- Different formats for Introduction of new TCP, Reclassification of new TCP as standard care and Change of use
- Provided to applicants to confirm the process undertaken, decisions made and action required
- Published on the TCPC internet site for transparency and accountability

Decision Summary (Appendix 9) ❖

Certificate of Approval (Appendix 10) ❖

5. APPLICATION PROCESS

BEST PRACTICE GUIDE

- Application submission deadlines allow sufficient time for adequate review by committee members
- Application forms for introduction of new TCPs are provided
- Application guidelines are available
- Application forms are not accepted if they are incomplete or if there is insufficient detail for decision-making
- Application forms are completed and submitted electronically
- Completed applications are forwarded to the Chair of the TCPC or other nominated delegate
- A register of applications and approved TCPs is maintained
- Applications require endorsement from Departmental Head and Program/Division Director
- Applicants, Department Heads and Program/Division Directors respond to queries raised by TCPC (at meeting or by correspondence)
- Application forms contain questions on all decision-making criteria
- Application forms meet the requirements of regional, state and/or national applications for introduction or funding of new TCPs
- Clinical need for TCP is addressed
- Evidence provided is based on a systematic review of the research literature
- Details of any assessment of the TCP by national health policy agency are provided (eg Australian Medical Services Advisory Committee)
- Objective and suitable expertise is used to identify the best available evidence from the research literature
- Objective and suitable expertise is used for issues relating to resources (financial, space, equipment, staff)
- Appropriate clinical and physical infrastructure/facilities exist to support the introduction of new TCPs
- Clinical and financial effects of each TCP are considered at all levels and in all departments
- The existing financial costs for current practice are estimated
- The projected financial costs for proposed TCP are estimated
- Appropriate, credentialed and trained staff are in place for the introduction of new TCPs
- Appropriate training is provided to all staff so that each TCP is performed and all equipment is handled safely
- Applicant completes Workforce Impact Statement considering current/future shortages, education and training, industrial issues, etc
- Evidence-based practice informs conditions and logistics for introduction
- Description of clinical governance arrangements and processes that oversee implementation of new TCP is provided
- A detailed implementation plan and timeframe for introducing new TCPs in a health service is provided
- Opportunities for disinvestment of current practices following introduction of new TCP are identified
- An evaluation protocol for the new TCP is provided (including all relevant indicators and defined time points)
- Relevant existing policies/procedures in the organisation are considered when introducing new TCPs
- Alternatives to the new TCP are listed and compared
- Contact details of external referees with experience in the new TCP are provided
- Applicant discusses 'Impact of Not Proceeding' considering patient safety, government policy, financial implications, service delivery, etc
- Include manufacturer, vendor, supplier information for purposes of describing new TCP, legal/contractual issues, etc
- Applicants are informed in writing of the outcome and recommendations of the committee
- Successful applicants are informed in writing of the conditions of implementation and reporting requirements
- Information about the TCP is disseminated and advice provided
- Procurement staff are aware of need for TCPC approval before purchases are made
- Application forms are provided for two year review, change of use of existing TCP and use of new TCP in research
- Issue of credentialing in emergency situations is addressed

MONASH HEALTH PROGRAM

Overview

- Application forms provide information for decision-making
- All forms are available on the TCPC webpage

- Instructions on how to complete the forms, who to contact for assistance and how to find, appraise, summarise and present research evidence are included within the application forms and on the website
- Applications and other documents must be submitted electronically to ensure legibility, enable electronic dissemination and reduce paper and filing requirements
- Applications must be submitted two weeks before the meeting. This allows one week for the Secretariat to ensure the application is appropriate and complete and one week for the TCPC members to read.
- All sections must be complete and in sufficient detail. Documents will be returned to applicant if inadequate.
- Applications are registered in a database and confirmation of receipt is provided to applicants

Introduction of new TCP

Requirements

- All new therapeutic interventions and diagnostic procedures must be approved by TCPC before introduction
- Staff in the Procurement Office are aware that purchase requisitions for new TCPs must have TCPC approval
- Manufacturers, vendors and suppliers are not permitted to submit a TCP application
- Applications must have endorsement from Head of Department and Program/Division Director
- The Applicant, Department Head and Program/Division Director are required to attend the TCPC meeting to respond to any matters raised so that a fair and informed assessment of the application is made.
- Applicants are informed of the outcome and receive a copy of the Decision Summary
- Successful applicants are informed of reporting requirements, dates for reporting and any special conditions

Application form

- Applications for introduction of new TCPs require very detailed information with supporting documentation to minimise risk to patients, clinicians and the organisation
- An application form based on guidance from the state health department was developed at the beginning of the project. Over time, it became clear that this form did not provide adequate information for decision-making. Applicants did not have the time and skills to find, appraise and synthesise information from the research literature and local data sources; they also had a vested interest in getting approval and often over-estimated benefits and under-estimated costs; and the application form did not provide a 'head-to-head' comparison of costs and health service utilisation between the new TCP and current practice. Although Monash Health no longer uses this form it meets the requirements of the state health department application for government funding for high cost TCPs and may be useful in this context.

Introduction of new TCP Application Form (Appendix 11) ❖

- A new model was proposed to improve the information provided for decision-making by utilising independent experts within the health service to summarise the evidence from research and local data and develop a business case for new TCPs. To minimise unnecessary resource use, the information is requested in stages, each stage dependant on a positive decision at the stage before.

Revised application process (Appendix 12) ❖

- Applicants submit an Expression of Interest in a much briefer document than the previous application form which greatly reduces their time commitment. The TCPC assesses whether the potential benefits of the new TCP and its fit within the organisation's goals and priorities is enough to warrant using additional resources to explore it further. If so, the TCPC commissions a Systematic Review of the research evidence by the Centre for Clinical Effectiveness. If there is sufficient evidence of increased safety, effectiveness and/or cost-effectiveness to proceed the TCPC commissions a Business Case to be undertaken by staff with expertise in these areas.

Introduction of new TCP Expression of Interest Form (Appendix 13) ❖

Additional inclusions

- Names of two referees from other Victorian, interstate or overseas health services with experience in the proposed TCP for external assessment
- Details of approval of the new TCP by the Australian Therapeutic Goods Administration (TGA); new TCPs will not be implemented without TGA approval

- Details of any reviews or recommendations by the Australian Medical Services Advisory Committee (MSAC). If there is Australian government policy regarding use of the proposed TCP, the TCPC does not require a full application, the existing policy will be followed.
- Declaration of potential Conflict of Interest for the Applicant and their colleagues
- Patient information brochure about the new TCP to enable informed consent
- Prompts to consider opportunities for disinvestment ie ceasing or restricting TCPs in current practice when new TCPs are introduced
- Templates for critical appraisals of included studies and tables for evidence summaries are provided in the Application Form

Request for Application

- Sometimes TCPs are introduced into the health service without following the correct process.
- If this is drawn to the attention of the TCPC a request for application is issued

Request for Application (Appendix 14) ❖

Two year review for reclassification of newly introduced TCP as standard practice

Requirements

- All applicants who introduced a new TCP are required to provide information for the two year review
- Applicants can request review earlier than two years by providing evidence to support the earlier date
- Applications must have endorsement of Program/Division Director, Executive Director and Business Manager

Application form

- In contrast with the Application form for introduction of a new TCP which requires detailed information with supporting documentation, the format of the two year review form is deliberately brief to minimise the applicant's workload
- A summary of the data collected for six monthly reports is included (number of patients, number of procedures, successful outcomes, deaths, adverse events)
- The main format is tick box 'Yes/No' responses to questions addressing the decision criteria
- The question format is consistent so that 'No' is the preferred response eg 'Has the TCP been used in any other way than that described in the original application?' If the TCP has been used as described in the original application the answer will be 'No'
- Further details are required if the response is 'Yes'
- TCPC may request additional information or require the applicant to attend a TCPC meeting if a decision cannot be made based on the information provided

Two year review Application Form (Appendix 15) ❖

Change of use of TCP in current practice

Requirements

- Use of TCPs in current practice may change due to new indication within the current patient group, new patient group, modification of equipment, new operators or practitioners, or other changes
- Before changing the use of a TCP in current practice, practitioners must inform the TCPC Chair or Executive Officer who will determine whether a Change of use application is required.
- Applications must have endorsement from Head of Department/Unit and Program/Division Director

Application form

- Like the Two year review form, the format is deliberately brief to minimise the applicant's workload
- Tick box 'Yes/No' responses are used to address the decision criteria
- The question format is consistent so that 'No' is the preferred response eg 'Will the change of use impact on other clinical disciplines or services?' If it will not impact on other disciplines or services, no further information is required
- Further details are required if the response is 'Yes'

- TCPC may request additional information or require the applicant to attend a TCPC meeting if a decision cannot be made based on the information provided

Change of use Application Form (Appendix 16) ❖

HREC application for research entailing a TCP new to the organisation

Requirements

- Researchers introducing a new TCP to the organisation in their clinical trials must complete this application in addition to the HREC forms
- Applications must have endorsement from Head of Department/Unit, Program/Division Director, Executive Director and Program/Division Director of any other affected departments/units
- Approval of a TCP as part of a research trial does not indicate support for introduction outside a research framework. Use of the TCP cannot be continued following the conclusion of a research project. A separate application must be made to the TCPC for introduction of the new TCP into clinical practice.

Application form

- Because it relates to introduction of a new TCP, this form requires detailed information
- Many questions have been taken directly from the Introduction of new TCP Application Form

HREC New TCP Application Form (Appendix 17) ❖

Use of new TCP in urgent or emergency situations

Requirements

- The patient must be at risk of harm if further intervention is not delivered
- All appropriate approved interventions have already been implemented
- There is insufficient time to go through the standard application process
- If approval is given, it is for 'once only' use

Application process

- Applicants seeking approval for immediate use of a new TCP or new use of a TCP in current practice must contact the Chair of the TCPC or the Chief Medical Officer

Feedback

- Each application form has a feedback page at the end
- Applicants are invited to comment on Content, Wording and Format of the document and Assistance provided in the process
- The questions 'What worked well and why?' 'What didn't work well and why?' 'How can we improve it?' are used in each section
- The example provided is the most detailed, shorter versions of this form were also used

Feedback Form (Appendix 18) ❖

Notification of compliance with reporting conditions

- Applicants are required to notify the TCPC that all relevant conditions have been met prior to implementation of approved TCP
- If this is not received by the due date, usually one month after approval, two reminders are sent by the Secretariat at monthly intervals
- If no response is received by the end of the third month, a letter is sent from the TCPC Chair notifying the applicant that permission to introduce the new TCP has been withdrawn

Notification of conditions (Appendix 19) ❖

6. MONITORING AND REPORTING

Best Practice Guide

- Processes and requirements for monitoring and reviewing existing TCP are determined
- Specified outcomes for each approved TCP are monitored and reviewed
- Applicants are required to notify TCPC that all specified conditions have been met prior to implementation of a new TCP
- Applicants are required to report specified outcomes to TCPC at agreed defined interval (eg six monthly for two years)
- Any adverse event occurring with a new TCP is notified to the TCPC
- Any adverse event occurring with a new TCP is notified to the relevant authority (if regulated eg Therapeutic Goods Administration)
- Ethics approval as a Quality Assurance activity is obtained prior to data collection
- Outcomes are collated in a database/register
- If the TCP carries risk of adverse events, criteria for reviewing outcomes are established prior to procedures being performed
- Regular reports are submitted to the state health department detailing applications, approvals, monitoring of new TCPs
- Regular reports (at an agreed/ defined interval) are submitted to the health service executive
- Local consumer health councils and networks will be informed of applications and of their outcomes
- Prompts are sent to applicants prior to the due date for reporting of outcomes
- Reminders are sent if outcomes reports are not received by the due date
- Permission to practice using the new TCP is withdrawn if outcomes reports are not received after a specified number of reminders

Monash Health Program

Overview

- A register of all applications and approved TCPs is maintained by the Secretariat
- Applicants are required to monitor outcomes, including adverse events, following introduction of a new TCP
- If the TCP carries known risk of specific adverse events, criteria for reviewing outcomes are established prior to procedures being performed

Ethics approval

- HREC approval of audit as a Quality Assurance activity is required prior to data collection
- The TCPC Secretariat facilitates this process by completing generic responses to questions that apply to all TCPs in the HREC Quality Assurance application. The applicant completes the details specific to the new TCP and submits the form.

Letter explaining compliance with Quality Assurance (Appendix 20) ❖

TCP Quality Assurance Application (Appendix 21) ❖

TCP Quality Assurance supplement letter (Appendix 22) ❖

Data collection

- Data to be collected includes number of patients referred, patients treated, procedures undertaken, successful outcomes, deaths and adverse events
- Outcome measures are collated in a generic spreadsheet provided by the TCPC Secretariat or in a data collection tool the applicant is already using.
  - Some clinicians have little experience in data collection and find the generic tool very useful. Presentation in a consistent format also facilitates collation of the data for TCPC summaries.
  - Other applicants have their own audit tools or may be collecting standardised datasets for national or international registries. They are not asked to duplicate this information in the generic tool but can submit the data in their own format.

- Data summaries are required at six monthly intervals for two years. The six monthly terms are fixed for more efficient administration (Jan-Jun and Jul-Dec).

Data Collection Spreadsheet (Appendix 23) ❖

Reporting

- Adverse events are to be reported immediately to both the TGA and the TCPC
- A Six Monthly Progress Report template is provided for consistency of reporting of each TCP
- Six Monthly Progress Reports are sent to the Secretariat. These are collated and reviewed by the TCPC
- The Six Monthly Reports plus a summary of TCPC activity detailing applications received, new TCPs approved, change of use applications and reclassifications of new TCPs as standard practice is provided to the state health department and the health service Executive Management Team every six months and to the health service Consumer Advisory Committee every 12 months

Progress Report Template (Appendix 24) ❖

Reminders

- Successful applicants are informed of their reporting requirements
- A prompt is sent by the Secretariat in May and November, one month before the end of each six monthly reporting period (Jan-Jun, Jul-Dec)
- If the progress report is not received by the due date, two reminders are sent by the Secretariat at monthly intervals
- If no response is received by the end of the third month, a letter is sent from the TCPC Chair notifying the applicant that permission to introduce the new TCP has been withdrawn

Correspondence regarding reporting compliance (Appendix 25) ❖

7. RESOURCES

BEST PRACTICE GUIDE

- Website housing documents and resources is developed and maintained
- Expertise in coding, data analysis, evidence review, finance, credentialing, contract negotiation and equipment maintenance requirements is provided
- Applicants are directed to guidance on finding the evidence of effectiveness of TCPs to support the application
- List of organisations that can provide evidence for the effectiveness of TCPs and in some cases service configuration is provided
- Assessment guidelines are provided ie how evidence submitted will be assessed
- Templates for appraising, summarising and presenting the evidence are provided
- Template for Patient information brochure is provided
- Templates for data collection tools and reporting proformas are provided (for therapeutic interventions and diagnostic tests)
- Formal Risk Assessment Tool is provided
- Business case template is provided
- Business case guidelines are available
- Life-cycle costing template is provided
- Guidance on approach for conducting economic evaluation is available

MONASH HEALTH PROGRAM

Website

- A website provides easy access to all TCPC information in one place
- As an internet site it is accessible to other health services and external interested parties
- It contains
  - Frequently asked questions
  - Information about the processes and supporting documents (eg Terms of Reference, Protocol, Meeting dates)
  - Application documents
  - Links to resources (eg people with expertise, guidance documents, templates)
  - Decision Summaries

TCPC website

Expertise and Support

- Expertise within the organisation is provided to improve the quality of information available for decision-making
- Expertise and support is provided in the following areas
  - Evidence from research literature: Centre for Clinical Effectiveness
  - Coding: Health Information Services
  - Current bed utilisation and costings: Clinical Information Management
  - Credentialing and scope of practice: Medical Workforce Unit
  - Proposed financial impact and business case: Finance Department
  - Infrastructure and equipment needs: Health Technology Services
- Names of liaison staff in the relevant departments and their contact details are provided on the website and within application documents

Online guidance

- To assist clinicians in completing the application form, CCE developed an online guide to finding, appraising and summarising the best available evidence relating to the new TCP
- It is a step-by-step toolkit that follows the questions in the application form

Finding the Evidence

Templates

- Templates are provided to assist applicants and to ensure processes and documents are consistent and of high quality
  - Patient Information brochure is mandatory
  - Patient outcome spreadsheet is optional if applicant already has a data collection tool (Appendix 23)
  - Progress Report is mandatory (Appendix 24)

Patient Information brochure template (Appendix 26) ❖

- Templates are also provided to assist administration of the TCPC

Agenda template (Appendix 27) ❖

Minutes template (Appendix 28) ❖

8. ADMINISTRATION

Best Practice Guide

- Staff with appropriate expertise and sufficient time are designated to manage the TCP program (eg Executive Officer, Administrator, etc)
- Systems, processes and resources are developed, implemented, maintained, evaluated and improved
- Processes are facilitated through checklists, timelines, diarising of due dates, electronic reminders, etc

Monash Health Program

Overview

- Administration involves development, implementation, maintenance, evaluation and improvement of all elements within the components of the Technology/Clinical Practice Program
- Administration is undertaken by the Executive Officer, Administrative Officer and the TCPC Chair

Secretariat

- Executive Officer and Administrative Officer
- The Executive Officer role has been undertaken by the Director of CCE (during the establishment phase) and the Medical Administration Registrar
- The Administrative Officer role has been undertaken by a CCE Project Officer (during the establishment phase) and an Executive Assistant
- Both roles have been undertaken simultaneously by the Medical Governance Officer and a CCE Consultant in Clinical Effectiveness
- The total amount of time spent by the Secretariat is difficult to define as it has varied over the lifetime of the TCPC
  - During the establishment and early implementation phase the time was approximately 1-2 hours/week for the Executive Officer and 1-2 days/week for the Administrative Officer
  - When other staff members took these positions the balance between the roles changed eg Executive Officer spent more time and the Administrative Officer less time
  - The total amount of time is heavily dependent on the number of applications to process

Activities

- Maintenance of systems, processes and resources
  - Website
  - Document management system including templates for agendas, minutes, reports, routine emails
  - Register of applications
  - Reporting database
  - Checklist of actions and timelines
  - Electronic calendar for due date reminder systems

Register of Applications (Appendix 29) ❖

Reporting database (Appendix 30) ❖

- Processing of applications
  - Providing information and assistance to address applicant's questions
  - Issuing confirmation of receipt
  - Checking for quality and completeness
  - Forwarding to TCPC members
- Correspondence with applicants, TCPC members, others
- Meeting management
  - Preparation: drafting agendas, sending invitations to applicants and co-opted guests, disseminating meeting papers

- Conduct: taking minutes, providing all relevant documentation to Chair, managing timetable of applicant arrival
- Actions: completing Decision Summaries, circulating Minutes and Decision Summaries after confirmation by Chair, providing successful applicants with information regarding introduction of the new TCP

- Compilation and circulation of reports
- Collation of responses from feedback pages on application forms and other documents
- Door signs with instructions for invited guests

9. EVALUATION AND QUALITY IMPROVEMENT

BEST PRACTICE GUIDE

- A framework and plan for evaluation of the TCP program is developed and implemented
- Data collection methods are established
- Application forms have feedback page to capture comments from users
- Feedback is sought from applicants on Content, Wording and Format of Application forms, Assistance provided and Resources available
- Feedback is sought from decision-makers and administrators on systems and processes
- Evaluation findings are published
- Improvements to systems, processes, documents and resources are implemented based on evaluation findings

MONASH HEALTH PROGRAM

Overview

- An Evaluation Framework and Plan was developed (Appendix 5)
- Feedback forms are attached to all documents and stakeholders are invited to provide feedback to Secretariat
- Modification or reinforcement of the program is based on the outcome of the periodic formal evaluations and the ongoing informal feedback
- The evaluation findings are published

Evaluation Report 2008 (Appendix 31) ❖

Evaluation Report 2009 (Appendix 32) ❖

APPENDIX 1. FRAMEWORK FOR EVIDENCE-BASED CHANGE

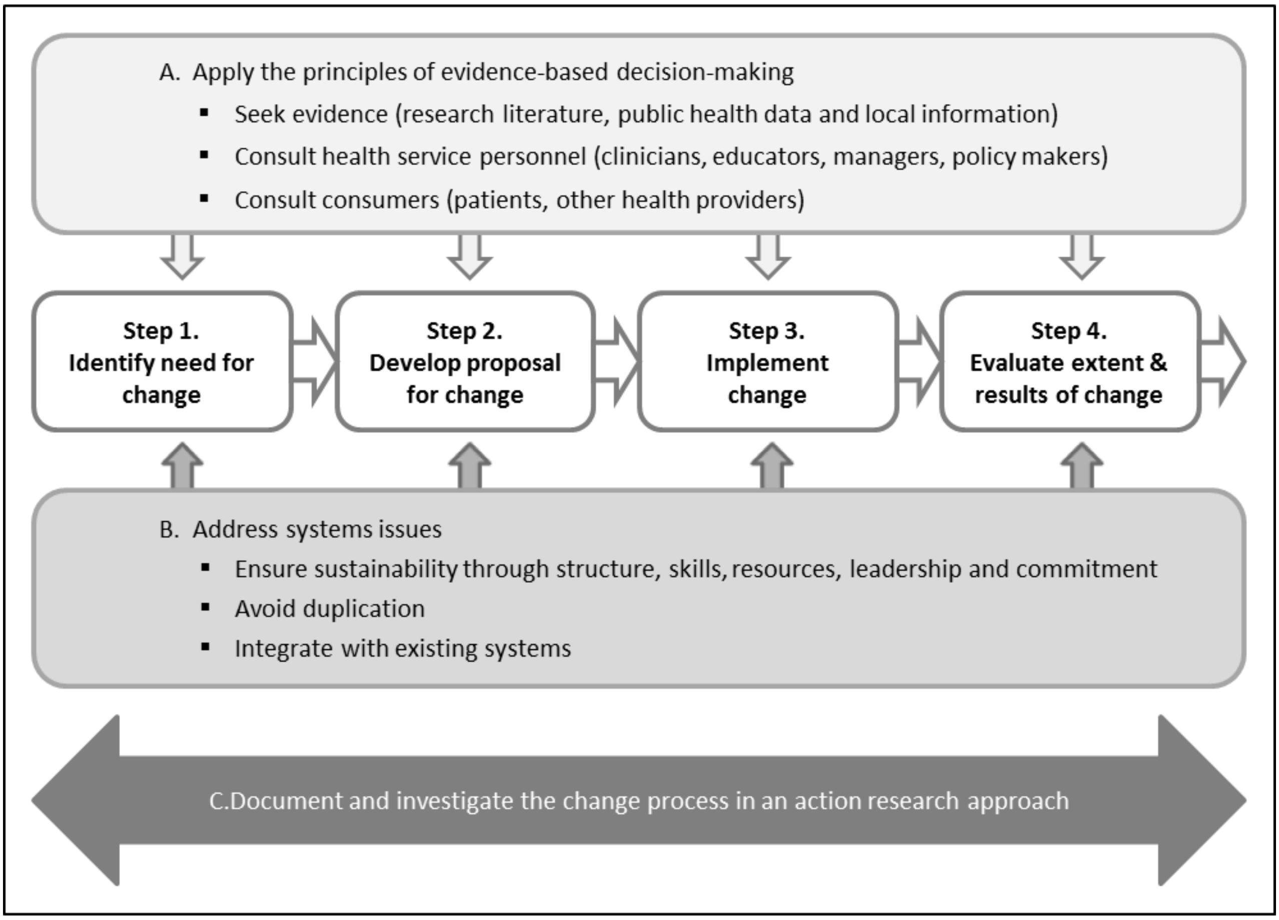

APPENDIX 2: TCPP: TIMELINE FOR DEVELOPMENT OF ORGANISATIONAL APPROACH, SYSTEMS AND PROCESSES

Phase One	Year 1												Year 2											
Briefing, preparation																								
Step 1: Identify need for change																								
Develop aims and objectives for project																								
Collate feedback from Monash Health staff																								
Map and collate existing practice																								
Seek current best practice (national/international)																								
Step 2: Develop proposed change																								
Identify changes required																								
Develop Program Logic Model																								
Develop aims and objectives for change proposal (meeting all requirements of the state health department guidance)																								
Develop change proposal (address sustainability, avoid duplication, facilitate existing)																								
Consult Southern Health data services																								
Refine proposal and timelines																								
Develop program components																								
Meet organisational requirements (eg Documentation Committee, Protocol authorisation, Printing, etc)																								
Schedule TCPC meetings							x	x	x	x	x	x	x	x	x	x	x	x	x	x	x	x	x	x
Seek TCPC endorsement for final proposal																								
Pre-pilot test of proposal with TCPC																								
Step 3: Implement proposed change																								
Develop TCP Webpage																								
Pilot proposal with VPACT Applications																								
Refine proposal based on pilot feedback and consultation with Department of Health																								
Present Report of Pilot to TCPC																								
Develop final Implementation Plan																								
Deliver Communication Plan																								

Implement new processes																								
Step 4: Evaluate change																								
Evaluate VPACT pilot (feedback forms, interview CIM, meeting for feedback on VPACT process)																								
Develop final Evaluation Plan																								
Undertake process evaluation																								
Collect outcome data																								
Analyse data																								
Report to TCPC																								
Disseminate findings																								

APPENDIX 3. BARRIERS AND ENABLERS AND STRATEGIES TO ADDRESS THEM

BARRIERS	STRATEGIES
**Economic and political context (financial arrangements, regulations, policies)**	
The state Department of Health has an annual funding round for new high cost TCPs. Stakeholders may be frustrated, confused or waste time with duplication if the TCPC documents have different content and format to the Department of Health.	Make TCPC application form meet Department of Health requirements for funding of high cost TCPs
The TCPC will be guided by decisions of the Australian Medical Services Advisory Committee (MSAC). All stakeholders will waste time and be frustrated if applicants are unaware of national policy and complete the application process unnecessarily.	Add a step in the process that requires the applicant to check for MSAC reviews on the new TCP. If the TCP is recommended by MSAC the applicant does not need to provide detailed evidence from research. If the TCP is not recommended, the application should not be continued.
Lack of finances to buy technologies	Addressed by explicit criteria to assess cost and affordability and transparency of publishing decisions
**Organisational context (organisation of care processes, staff, capacities, resources, structures)**	
TCPC may not be held in sufficiently high regard for applicants to respect and abide by processes	Introduce mandatory policy that all new TCPs must go through new authorisation process
	Raise profile and influence of TCPC by upgrading committee relationship structure so that TCPC reports to the Executive Management Team
Decision-maker's lack of time to read extensive documentation prior to meeting due to busy workloads	Secretariat to provide all documents at least one week prior to meeting
	Secretariat to do all the 'work' of the committee (eg preparation, following up actions, etc)
Applicant's lack of time to complete application form for introduction of new TCP due to busy workloads	Make application form as user-friendly as possible eg use 'tick boxes' as alternatives to free text
	CCE staff provide help to find evidence eg assistance with searches
	TCPC Secretariat to provide assistance with document completion in the initial phase so that applicants can see what is required of them
Applicant's lack of time to complete application form for Change of use and Two year review due to busy workloads	Use 'tick box' format throughout
	Accept documented declaration by applicant of endorsement by Program Director, Executive Director and Business Manager without actual signatures required
Administrator's lack of time to manage the proposed processes of the seven new components due to no time allocation for TCPC processes	Allocate resources by diverting CCE staff time from other areas to TCPC. CCE Director as Executive Officer (1-2 hours/week) and a CCE Project Officer as Administrator (1-2 days/week).
Specialist resource staff (eg coders, data analysts) lack of time to provide adequate information for decision-making due to applicants leaving requests to the last minute	Introduce time limits eg Applicants must contact coders at least two days and data analysts at least two weeks before information is required
	Include instructions in the application form regarding deadlines for support services
Six-monthly reports to monitor new TCPs based on the date of introduction are inconvenient, confusing and create extra work due to multiple deadlines	Change all reporting periods to single format (Jan-Jun and Jul-Dec). Applicants may report for part of the first and last six-monthly period if they introduce a new TCP in that time frame
Lack of central source of information for TCPC processes	Create and promote a webpage to house all information, documents and resources
Purchases will continue to be made without appropriate authorisation	Inform Procurement Department of requirements and involve Procurement Director in program

Decision-makers cannot attend meetings due to other commitments	Set meeting dates in advance to maximise attendance and allow appropriate representation
	Encourage those unable to attend to provide feedback regarding agenda items at the time of apology
**Social context (opinion of colleagues, culture of the network, collaboration, leadership)**	
CCE Project team has no role in the process therefore limited influence	Make Project team responsible for the process. CCE staff become the TCPC Secretariat
Potential duplication of activities between the project team and TCPC administrators	
Decision-makers under pressure to approve applications, particularly if new TCP in use elsewhere eg overseas, in private hospitals	Addressed by program elements to achieve transparency, accountability and EB decisions eg Explicit criteria, published Decision Summaries, etc
	Seek support for these principles from Executive Management Team and health service Board
Applicants perceive that health service management priorities are about saving money	Promote decision-making principles, stress safety and effectiveness, better patient outcomes, etc
Power and budget struggles affecting perceptions and acceptance of decisions	Addressed by program elements to achieve transparency, accountability and EB decisions eg Explicit criteria, published Decision Summaries, etc
**Patient (knowledge, skills, attitude, compliance)**	
Lack of consumer input if single consumer representative unable to attend meeting	Increase to two consumer representatives
Evidence that having less than two consumers on committees is not best practice	
Applicants do not know how to write high quality patient information (usually too much, too technical and omits information the patient wants to know)	Include input from consumer representatives on draft patient information materials
	Develop template for patient information
Consumer representatives will incur costs to print out meeting papers	Send hardcopy of papers in Express Post at same time as electronic circulation
**Individual professional (awareness, knowledge, attitude, motivation to change, behavioural routines)**	
Applicant's lack of awareness of process and requirements	Introduce mandatory policy that all new TCPs must go through new authorisation process
	Develop protocol outlining steps in new processes
	Explain reasons for new processes in communication strategy
	Notify all staff via organisational newsletters
	Send bulletins to likely applicants via All Managers, Dept Head and Senior Medical Staff email lists
	Hold face-to-face meetings with Medical Program Directors
	Communicate with Managers of Operating Suites and Procedural facilities
	Inform Procurement Department of requirements and involve Procurement Director in program
	Create and promote a webpage to house all information, documents and resources
	Require that use of new TCP introduced without authorisation is ceased until process is complete

Applicant's lack of knowledge regarding what should be considered a 'new TCP' or 'Change of use' and when applications are required	Provide clear definitions for 'new TCP' and 'Change of use' and instructions for when applications are required
Applicant's lack of autonomy: unwillingness to submit control to application process or to wait until process complete before commencing	Same as lack of awareness (above)
Applicant's belief in benefit of TCP: use new TCP without authorisation to do what they think is best for their patients	Same as lack of awareness (above)
Applicants forget to apply	Same as lack of awareness (above)
Applicant's animosity towards 'red tape'	Same as lack of awareness (above)
	Remove any unnecessary 'red tape'
	TCPC Secretariat to be welcoming, respond to enquiries, provide information and assistance, etc
Applicants do not usually have the appropriate skills to provide the level of detail and quality of information required for decision-making	Provide assistance from relevant experts within the organization eg CCE (evidence), Health Information Services (coding), Clinical Information Management (health service utilisation data), Medical Support Unit (credentialing) and Finance Department (business case).
Applicants do not usually have the appropriate skills in systematic review methods and are often not familiar with the sources of high quality evidence	Develop step-by-step 'Guide to Finding the Evidence' that follows the sequence of questions on the application form.
Applicants continue to provide low level or non-research evidence, or do not use a systematic approach therefore do not provide the best available evidence)	Be explicit about requirement for high level evidence, appropriate evidence re safety, etc
	Provide tools to identify best available evidence and templates to document it
Applicant's frustration with lack of timeliness or relevance of research	Explain that high level high quality evidence is required to introduce change across the organisation
Applicant's frustration with poor quality of research	Explain that high level high quality evidence is required to introduce change across the organisation
Applicants do not monitor and/or report outcomes	Provide prompts one month before deadline
	Issue monthly reminders after deadline
	Withdraw permission to use TCP if no response to second reminder
Applicant's poor handwriting, application difficult to read	Require electronic submission of documents
Applicant's lack of experience in word processing (some senior medical staff had never created an electronic document before)	TCPC Secretariat to provide assistance with document completion in the initial phase so that applicants can see what is required of them
	TCPC Secretariat to help Applicant's Personal/Executive Assistants understand the requirements
Sections of document incomplete or inadequate detail provided	Provide alternatives with 'tick boxes' where appropriate
	TCPC Secretariat to provide assistance when the problem is due to lack of technical expertise
	Require application two weeks before meeting – one for Secretariat to check and one for TCPC to read
	Return document to applicant for completion
Many applicants do not know how to collect data, which data collection tools to use, etc therefore quality may be poor and collation very time-consuming	Create generic data collection tool

Some applicants are very experienced in collecting data and may even be collecting standardised data sets for national or international registries so do not want to duplicate data collection by using generic TCPC tool in addition to their own	Allow generic tool to be optional if applicants already have well developed audit methods
**Innovation (advantages in practice, feasibility, credibility, accessibility, attractiveness)**	
New processes may lack credibility as there is no clear evidence or recognised experts to determine process for introduction of new TCPs if applicants do not consider the national, state and professional bodies who produced the guidance to be credible organisations	Promote decision-making principles, stress safety and effectiveness, better patient outcomes, etc
	Explain role of local consultation in development process
	Explain role of ongoing feedback to allow local needs to influence program
New process is highly complex and requires time, skills and expertise	Same as lack of credibility (above)
	Make processes and documents as simple and user-friendly as possible
	Seek ongoing feedback and refine processes and documents based on feedback
Application form detailed, complicated and probably not attractive to applicants	Same as lack of credibility (above) and high complexity (above)
New process is significantly different from status quo	Same as lack of credibility (above) and high complexity (above)
Applicants may not consider the new program to have any advantages over status quo	Promote decision-making principles, stress safety and effectiveness, better patient outcomes, etc
Applicants have difficulty accessing documents	Create and promote a webpage to house all information, documents and resources
**ENABLERS**	**STRATEGIES**
New TCPC processes are a high priority for the organisation	Raise profile and influence of TCPC by upgrading committee relationship structure so that TCPC reports to the Executive Management Team
The organisation is committed to the new TCPC processes	Introduce mandatory policy that all new TCPs must go through new authorisation process
Funding has been provided to establish the new program	Use rigorous methods to develop, implement and evaluate the new program
CCE has high level skills in Evidence Based Practice	Make Project team responsible for the process. CCE staff become the TCPC Secretariat
The decision-makers and project team are willing to change the system based on feedback	Make feedback process known, act upon it, advertise that changes are based on feedback received
improving patient outcomes is known to be a motivator for clinical staff	Promote and explain how processes enhance safe and effective care
All clinical Program/Division Directors are supportive	Use Program/Division Directors to communicate within their programs/divisions
Chair of TCPC highly regarded by applicants and influential within the organisation	TCPC Chair to correspond (in person or in writing) when seeking influence for change or to communicate with applicants who are not following the process
TCPC decision-makers are committed to evidence-based decision-making	Build in rigorous and explicit methods of evidence based practice
Ethics approval processes are well established and accepted in the organisation	Cite ethics process as example of similar system that is both rigorous and familiar to stakeholders
	Addressed by program elements to streamline processes between committees

APPENDIX 4. INTRODUCTORY CORRESPONDENCE

<Date>

<Name>
<Position><Department>
Southern Health

Dear <Name>,

Re: Introduction of new Technologies/Clinical Practices (TCPs) at Southern Health

I would like to make you aware of changes to the processes regarding introduction of new TCPs.

The Southern Health 'New Clinical Procedures Committee' has been replaced by the 'Technology/Clinical Practice Committee' (TCPC) based on recent Department of Human Services (DHS) guidance. The Centre for Clinical Effectiveness (CCE) will undertake responsibilities as Secretariat to the TCPC and will manage the application procedures, monitoring and reporting for all new technologies/clinical practices at Southern Health.

New technologies/clinical practices are defined as therapeutic interventions (including prostheses, implantable devices; high cost pharmaceuticals; medical, surgical or other clinical procedures) or diagnostic procedures that are considered by a reasonable body of clinical opinion to be significantly different from existing clinical practice.

Approval **must** be obtained from the TCPC **before** commencing any new technologies/clinical practices on any Southern Health patient, private or public.

Applications are required for:

- Introduction of a new TCP that has not previously been performed at Southern Health
- Variations to existing procedures or treatments if they involve a new device or item of equipment, or if considered by a reasonable body of clinical opinion to be significantly different from existing practice

Applications are not required when:

- A clinician proposes to use a TCP that is already in practice at Southern Health but has not previously been undertaken by that individual

As part of the new administration process the TCPC will meet **once a month** to review applications. It is requested that applications for new TCPs be received two weeks prior to the meeting date.

Department/Unit Heads will be responsible for signing off on completed applications from their department/unit prior to submission to the TCPC. They are also required to attend the scheduled TCPC meeting for review of any applications from their department/unit along with the applicant and relevant Program Director.

All relevant documentation, resources and meeting dates and application deadlines are available online here.

This system is currently in a piloting phase and we welcome any suggestions for improvement related to the processes involved or the content and format of the documents.

If you have any further queries please contact
Dr Claire Harris, Director, Centre for Clinical Effectiveness (9594 7576 cce@med.monash.edu.au)

Yours sincerely,

A/Prof Richard King
Chair, Southern Health Technology/Clinical Practice Committee
Program Director, Medicine Program

<Date>

Dear Colleagues,

Re: 'Change of Use' applications for technologies and clinical practices at Southern Health

Changes to a technology or clinical practice in current use such as modification to equipment, increase in scope, new operators, etc are usually straightforward and have minimal impact. However it is important to assess whether the change of use brings with it any potential risks to patients, clinicians and the organisation. These risks may relate to injury, damage to reputation or financial or legal consequences.

The Southern Health Technology/Clinical Practice Committee (TCPC) has developed a screening tool which is designed to inform the Committee of any changes to technologies or clinical practices in current use and to identify any risks as a result of the change.

The TCPC have tried to make this process as easy as possible for clinicians and not overburden already busy people with additional work to complete applications. If there are no risks, the form should be quick to complete by just answering 'no' to each question. If there are potential risks, then it is important that a little bit of time is spent filling in some details for the TCPC to review. The form does not require a signature or any substantiating information as it relies on honest replies to minimise the work involved.

The 'Change of Use' form is included below and available from http://www.mihsr.monash.org/cce/doc/cou_applicationform.doc

The Committee has piloted the current 'Change of Use' application form with five applications in 2008. A summary of these applications is included below and available from http://www.mihsr.monash.org/cce/pdf/cou_summaryofdecisions2008.pdf.

This system is currently in a piloting phase and we welcome any suggestions for improvement related to the processes involved or the content and format of the document.

Please contact me if you would like any further information about this process
Yours sincerely,

Dr Claire Harris
Director, Centre for Clinical Effectiveness
Secretariat, Southern Health Technology/Clinical Practice Committee
Phone: 9594 7576
Email: claire.harris@med.monash.edu.au

<Date>

<Name>
<Position><Department>
Southern Health

Dear <Name>,

Re: Audit and reporting following introduction of new Technologies/Clinical Practices (TCPs)

As you know, there have been recent changes to the processes regarding introduction of new TCPs at Southern Health. The Southern Health 'New Clinical Procedures Committee' has been replaced by the 'Technology/Clinical Practice Committee' (TCPC) based on recent guidance from Department of Human Services (DHS). The Centre for Clinical Effectiveness (CCE) undertakes responsibilities as Secretariat to the TCPC and will manage the application process, monitoring and reporting for all new technologies/clinical practices at Southern Health.

On <Date> the Southern Health New Clinical Procedures Committee approved the application from your department for **<Application Number and Title>.** Approval was conditional upon audit and reporting of patient outcomes.

In order to meet reporting requirements set by DHS, CCE will be collecting data for all new TCPs implemented at Southern Health. We have developed proformas to make the process of capturing and reporting this information as easy as possible for successful applicants. A draft report template and an audit spreadsheet are attached. You may find these useful.

The report template is from DHS, however we think there is room for improvement in both content and format. We are implementing this process as a pilot, please feel free to suggest any changes. A feedback form is attached to the report template or you can contact CCE directly by phone or email

Similarly, any suggestions to improve the outcomes spreadsheet are welcome. If you already have an outcome auditing system in place please feel free to forward the required information to us in your current format.

Please complete data entry up to <Date>

We have allowed 8 weeks for your response.

Please email your response to CCE cce@med.monash.edu.au by **COB** <**insert appropriate date here>**.

If you require help with this process or have any further queries or feedback please contact

Ms Marie Garrubba
TCPC Administrator
9594 7553
cce@med.monash.edu.au

Yours sincerely,

Dr Claire Harris
Director, Centre for Clinical Effectiveness
Executive Officer, Southern Health Technology/Clinical Practice Committee

Cc: <Applicant>

APPENDIX 5. EVALUATION PLAN

Components	Key evaluation questions	Measures/Indicators	Data collection method and source	Timing of Collection	Timing of Reporting
Establishment of best practice	Does the Monash Health TCP Program match current best practice?	Current best practice – Evidence Mapping	Revise mapping exercise of State/National/International Sources	End of establishment phase: 3-5 year intervals	
Governance	Is the process transparent and accountable?	Publication of TOR, procedure protocols, application deadlines, meeting dates	Review of TCPC website, Monash Health intranet	Annually	Annually
		Attendance at meetings	Attendance list	Monthly	Annually
		Feedback from TCPC re processes	TCPC meetings – review minutes	Annually	Annually
		Achieving reporting requirements for EMT and DHS	Reports sent	Biannually	Biannually
		Appropriateness of reporting to EMT and DHS	Feedback from EMT and DHS	Biannually	Biannually
Applications New TCPs	Has an application process and documentation in accordance with DHS requirements been established and is it being utilised?<br>Are applicants happy with the process?	Number of applications received	Audit of TCP register	Monthly	Annually
		Number of applications completed correctly at first submission	Audit of TCP register	Monthly	Annually
		Applicant satisfaction with application process	Audit of application feedback forms	Monthly	Annually
		Number of VPACT applications approved by DHS	DHS feedback	Monthly	Annually
		Compliance with the Monash Health VPACT schedule	Audit of VPACT timetable	Annually	Annually
		Comparison with other health services<br>▪ Number of applications received<br>▪ Comparison of applications (same/different)<br>▪ Were the same decisions made	Collect this information from the group that DHS sets up	Annually	Annually
	Did we capture all TCPs introduced at Monash Health	Number of TCPs introduced at Monash Health that did not go through the TCPC process	Query Unit Managers and Operating Suite<br>Query Procurement and Diagnostic Services<br>Query high cost drug list from Pharmacy<br>Query presentations made at 'State of the Art' Lectures and Grand Rounds<br>Query Capital Expenditure process	Quarterly	Annually
Applications COU of existing TCPs	Has a change of use application process and documentation been established and is it being utilised?<br>Are applicants happy with the process?	Number of applications received	Audit of TCP register	Monthly	Annually
		Number of applications completed correctly at first submission	Audit of TCP register	Monthly	Annually
		Applicant satisfaction with COU application process	Audit of application feedback forms	Monthly	Annually

Components	Key evaluation questions	Measures/Indicators	Data collection method and source	Timing of Collection	Timing of Reporting
Decision-making<br>New TCPs	Have processes and documentation for decision-making been established and are they being utilised?	Appropriate representation at TCPC meetings to discuss applications	Audit of minutes for attendance by applicant/HOD/Program Director	Monthly	Annually
		Number of applications with decision summaries	Audit of application folders	Monthly	Annually
		Number of decision summaries published on website	Cross check applications with webpage	Monthly	Annually
		Number of applicants that complied with conditions of approval and were received by the due date	Audit of TCP register	Monthly	Annually
		Number of outcome letters for approval for provisional use sent	Audit of TCP register	Monthly	Annually
		Number of applicants that appealed the TCPC decision	Audit of TCP register	Monthly	Annually
Decision-making<br>Review of approved TCPs	Have processes and documentation for decision-making for reviewed TCPs been established and are they being utilised?	Number of applications with decision summaries	Audit of application folders	Monthly	Annually
		Number of decision summaries published on website	Cross check applications with webpage	Monthly	Annually
		Number of TCPs withdrawn after review	Audit of TCP register	Monthly	Annually
		Number of decisions made that were consistent with the evidence	Review of the evidence	Monthly	Annually
Monitoring and reporting for newly introduced TCPs	Have monitoring and reporting processes been established and are they being utilised?<br>Are applicants happy with the process?<br>Were patient outcomes as expected?	Number of reports<br>▪ Received<br>▪ Received by due date<br>▪ Received late	Audit of TCP register	Biannually	Annually
		Number of applicants who used TCPC spreadsheet	Audit of TCP register	Biannually	Annually
		Number of applicants who used their own outcome data collection tool	Audit of TCP register	Biannually	Annually
		Number of reporting templates completed correctly	Audit of TCP register	Biannually	Annually
		Applicant satisfaction with reporting processes	Audit of application feedback forms	Biannually	Annually
		Number of procedures performed<br>Number of referred versus treated<br>Number of expected versus actual<br>Number of deaths<br>Number of other adverse events	Comparison between original applications and progress report data	Biannually	Biannually
Resources	Has a support system and resource documents been developed and are these being utilised?	Number of applicants that utilised patient information template	Audit of application documents	Biannually	Annually
		Applicant satisfaction with quality and accessibility of resources	Audit of application feedback forms	Monthly	Annually
		Feedback from resource providers	Via formal meeting or email request	Biannually	Annually
		Number of requests for use of resources/expertise	Audit of requests	Annually	Annually

APPENDIX 6. POLICY DOCUMENT

Safe Introduction of a New Technology or Clinical Practice **Operational Policy**

MonashHealth

**Policy Statement**<br>Monash Health will ensure that any new health technologies or clinical practices which are introduced are supported by evidence of appropriateness, safety, clinical effectiveness and are financially sustainable.
**Who must comply with this policy?**<br>All clinical staff
**This policy applies to:**<br>This policy applies to all Monash Health staff who wish to introduce a new health technology or clinical practice to Monash Health.
**List of Monash Health Procedures that link to this Operational Policy (under development)**<br>• Introduction, monitoring of a new technology or clinical practice (attached)<br>• "Change of Use" of an existing technology or clinical practice (attached)<br>• Managing a new technology or clinical practice in the context of a research project (under development)<br>• Introduction of new technology or clinical practice under the Victorian Policy Advisory Committee on Technology funding program (under development)
**Evaluation, monitoring and reporting of compliance to this policy**<br>Adherence to this policy will be monitored and evaluated through:<br>Six monthly report to Monash Health Executive Management Team on the following indicators:<br>1. Number of "New Applications" approved<br>2. Number of "Change of Use" applications approved<br>3. Adverse events related to an approved new technology (if any)<br>4. Number of approved applications being monitored
**Keywords or tags**<br>TCP, TCPC, new intervention, new procedure, VPACT

Document management
**Policy supported:** Safe, effective, person-centred care
**Background:** Safe introduction of new technology or clinical practice
**Executive Sponsor:** Executive Director Medical Services and Quality
**Person Responsible:** Executive Officer Technology/Clinical Practice Committee
**Authorisation Date:** 19/04/2011
**Review Date:** 19/04/2014
**Version Number:** 1

**Who**	All Southern Health Clinicians
**Expected Outcomes**	All clinicians will be aware of the process for introducing a technology/clinical practice at Southern Health
**Precautions**	Approval **must** be obtained from the Technology/Clinical Practice Committee (TCPC) **before** commencing use of any new technologies or clinical practices on any Southern Health patient, private or public. It is imperative that approval by the TCPC be obtained before the new technology/clinical practice is carried out at Southern Health. There may be adverse legal implications for both the clinician and Southern Health if approval from the TCPC has not been obtained.<br>Any new interventions undergoing development and/or trial are to be considered as experimentation or research and must, in addition, be reviewed by the Southern Health Human Research Ethics Committee.
**Why**	Encourage Southern Health clinicians to engage with new technology/clinical practice which have the potential to improve the provision of healthcare, within a framework which protects the interests of patients, clinicians and the organisation.<br>To ensure that:<br>▪ the ramifications of each new technology/clinical practice are considered at all levels and in all departments<br>▪ appropriate training is provided to all staff so that each new practice is performed (and new equipment is handled) safely<br>▪ every patient is cared for safely and appropriately throughout an episode of care.
**Definition**	A new "Technology/Clinical Practice" is a therapeutic intervention (including prostheses; implantable devices; high cost pharmaceuticals; medical, surgical or other clinical procedures) or diagnostic procedure that is considered by a reasonable body of clinical opinion to be significantly different from existing clinical practice.<br>It includes a procedure that has not been performed at Southern Health, as well as any variation to an existing procedure or treatment where a new device or item of equipment is introduced, including situations where new devices are provided by manufacturers without charge.<br>A new "Technology/Clinical Practice" does **not** include the situation where a clinician proposes to use a technology/clinical practice that is already being undertaken within Southern Health but which has not been previously used by that clinician. (DHS, 2006)
**Role of TCPC**	The primary role of the TCPC is to oversee and support Southern Health in the safe and appropriate introduction of a technology/clinical practice that has not previously been undertaken.<br>**Aim**<br>To establish a process that will facilitate quality and safety and protect patients, clinicians, managers and the organisation in ensuring that:<br>▪ the clinical and financial effects of each TCP are considered at all levels and in all departments<br>▪ appropriate training is provided to all staff so that each TCP is performed and equipment is handled safely<br>▪ every patient is offered the opportunity to give valid consent to new procedures and is cared for safely and appropriately throughout an episode of care.<br>The TCPC reviews written submissions from clinicians within Southern Health who wish to introduce a new technology/clinical practice. Each review will consider the following criteria:<br>1. Safety: What are the main adverse events? Safety in relation to current practice?<br>2. Effectiveness: Volume of evidence, consistency, clinical impact, generalisability and applicability<br>3. Cost: How affordable is the new technology/clinical practice? Does the cost represent value for money?<br>4. Clinical Feasibility: Resource implications and credentialing and competency assurance undertaken<br>5. Access and Equity<br>6. Legal and Ethical Implications

	Once a new technology/clinical practice is approved, the secondary role of the TCPC is to monitor the performance of that procedure for 2 years or such other period as the TCPC may consider appropriate.<br>The Centre for Clinical Effectiveness (CCE) is the Secretariat for the Southern Health TCPC
**Who may apply to the TCPC?**	Applicants may be:<br>▪ Individual clinicians seeking to introduce a new technology/clinical practice.<br>▪ Heads of Departments/Units may refer a matter for the attention of the TCPC where a new technology/clinical practice is sought to be introduced by a staff member
**Written Submissions**	Any clinician who wishes to introduce a new technology/clinical practice is required to provide a written submission to the TCPC, which includes the following:<br>▪ Summary of information<br>▪ Conflict of interest statement<br>▪ Overview of technology/clinical practice<br>▪ Clinical need<br>▪ Evidence of safety, efficacy and clinical effectiveness<br>▪ Evidence of cost effectiveness<br>▪ Clinical feasibility<br>▪ Governance<br>▪ Estimated financial impact<br>▪ Implementation<br>▪ Patient Information
**Equipment**	Application forms and related resources and templates are available online http://www.mihsr.monash.org/cce/shtcp.html or from the Secretariat of the TCPC, as detailed below.
**Step 1**	Advice regarding appropriateness of the submission or assistance with completion of the application form can be sought from the Secretariat of the TCPC.<br>**Administrative matters**<br>Ms Marie Garrubba<br>TCPC Administrator<br>Phone: 9594 7553<br>Email: marie.garrubba@southernhealth.org.au<br>**Clinical matters**<br>Dr Claire Harris<br>Director, Centre for Clinical Effectiveness<br>Phone: 9594 7576<br>Email: claire.harris@southernhealth.org.au<br>**Note: All applications are to be completed and returned electronically to the Secretary of the TCPC.**
**Step 2**	The applicant consults with and obtains approval from their unit head before lodging the electronic submission.
**Step 3**	TCP applications must be submitted before the due date for the next scheduled meeting of the TCPC to enable them to be circulated with the agenda.<br>Where there is a genuine urgency attached to a request for approval, a short explanation should be attached to the electronic submission and brought to the attention of the Secretary.
**Step 4**	Applications will be registered and confirmation of receipt provided to applicants.
**Step 5**	Unless otherwise notified the applicant will be required to attend a brief meeting with the TCPC to respond to any matters raised by its members so that a fair and informed assessment of the application is made.
**Step 6**	The TCPC consults with the relevant Head(s) of Departments/Units and Program Director(s).
**Step 7**	The TCPC, as part of its review process, determines whether each person proposing to introduce the TCP is both competent and credentialed, and whether there is any conflict of interest.
**Step 8**	After consideration of all of the material presented, the TCPC makes a decision in respect to the application.
**Step 9**	Communication with applicants concerning changes in their application recommended by the committee will take place through the Secretary.
**Step 10**	The Chair of the Committee will advise the Southern Health Executive of applications and outcomes of the Committee's decision.
**Step 11**	Applicants will be informed of the outcome of the application. Successful applicants will be informed of reporting requirements and the dates for reporting.

**Step 12**	Unsuccessful applicants may appeal to the Southern Health Chief Executive.
**Step 13**	The relevant Head of Department/Unit will provide a Progress Report to the Committee at six monthly intervals (January – June and July – December) for all patients referred and treated. http://www.mihsr.monash.org/cce/doc/tcpc_progressreport.doc
**Step 14**	The applicant must notify the Secretariat in writing of any minor changes made, in light of actual experience, to the technology/clinical practice as approved by the TCPC. The Secretariat will advise applicants if a 'change of use' application form is required to be submitted.
**Step 15**	If an adverse event occurs with an approved TCP, the applicant or relevant Head of Department/Unit must immediately notify the TCPC and the Therapeutic Goods Administration.<br>The applicant must also complete a Southern Health incident report form.<br>Please see Southern Health protocol CP-QR01 'Incident reporting'.
**Step 16**	If, in case of a genuine emergency, approval is required for immediate use of a new clinical practice, including a new or new use of a device, one use only approval may be given by either the Chair of the TCPC or the Chief Medical Officer.
**Step 17**	Unless otherwise determined, proceedings of the Committee shall remain confidential.

References

- NSW Health 2003. *Model Policy for the Safe introduction of New Interventional Procedures into Clinical Practice*. Circular No.2003/84
- NACS/ASERNIP-S. *General Guidelines for Assessing Approving and Introducing New Procedures into a Hospital or Health Service.* Royal Australasian College of Surgeons, Melbourne.
- NHS. *National Institute for Clinical Excellence, Interventional Procedures at http://www.nice.org.au*
- DHS. 2006. *Guideline for Health services to establish Technology/Clinical Practice Committees*. Department of Human Services, Victoria.

SH Policy	Quality and Risk Management	ACHS	Leadership and Management
Reviewer	Director, Centre for Clinical Effectiveness	Last review date	March 2009
Authoriser	Chair, Technology/Clinical Practice Committee	Next review date	March 2011

This hard copy may not be the latest version of this document.
Please see the Southern Health Policy and Protocol intranet site for current policies, protocols and guidelines.

APPENDIX 7. TECHNOLOGY/CLINICAL PRACTICE COMMITTEE TERMS OF REFERENCE

Background

A health technology/clinical practice (TCP) is defined as a therapeutic intervention (including prostheses; implantable devices; high cost pharmaceuticals; medical, surgical or other clinical procedures) or diagnostic procedure.

Role

To facilitate quality and safety and protect patients, clinicians, managers and the organisation in ensuring that:

- Introduction of new technologies and clinical practices at Monash Health is supported by evidence of safety, clinical effectiveness and cost effectiveness.
- TCPs in current use are consistent with the best available evidence.
- The capital and operating costs of each new TCP are considered at all levels and in all departments.
- Technologies in current use for which there is evidence of harm, lack of effectiveness or lack of cost effectiveness are considered for disinvestment.
- Proposed human research projects submitted for scientific and ethical review to the Human Research Ethics Committee conform to relevant principles and standards of credentialing, safety, clinical effectiveness and cost effectiveness and to provide advice to the Human Research Ethics Committee as required from time to time on research projects.

Membership

The Executive Sponsor of the Technology/Clinical Practice Committee will be appointed by the Executive Management Team. Committee appointments will include:

**Permanent Members**	
Chair	Appointed by Executive Management Team
Executive Sponsor	Executive Director, Medical Services
Executive Officer	Representative, Centre for Clinical Effectiveness
Legal/Ethics	Director, Research Services
Evidence	Representative, Centre for Clinical Effectiveness
Operational/Financial	Manager, Operating Suite
Consumer Representatives	Two representatives by invitation
Program Directors or their alternates	• Director Medical Services<br>• Medicine Program (Chair)<br>• Surgery Program<br>• Critical Care Program<br>• Mental Health Program<br>• Specialty Program<br>• Women's & Children's Program<br>• General Medicine Program<br>• Emergency Care Program<br>• Representative of Executive Director Nursing and Midwifery or alternate<br>• General Manager, Allied Health or alternate
Representatives of related Committees	Chair, Therapeutics Committee<br>Chair, Product Evaluation Committee
**Non-Permanent Members**	
Heads of relevant departments	Pathology<br>Radiology<br>Pharmacy

Quorum

50% (9) permanent members with at least three clinicians must be present to meet quorum requirements. At least one consumer representative should also be present but if both are unable to attend they will be sent the documentation for opinion.

The Department Head submitting the application will attend with the applicant but will not be considered members of the committee for the purpose of establishing a quorum.

Responsibilities

- To assess consistency of current practice at Monash Health with the best available evidence.
- To assess applications to introduce a new TCP into Monash Health.
- To assess applications for change of use of current TCPs approved at Monash Health.
- To maintain a register of all applications and approved TCPs.
- To determine data to be collected and reporting intervals.
- To maintain a database of follow up data and adverse events of submitted and approved TCPs.
- To review approved TCPs two years after implementation (or earlier as required) to assess whether they can be reclassified as standard practice.
- To prepare reports for the Monash Health Executive Management Team (EMT).
- To prepare reports for the Victorian Department of Health.
- To review referred existing TCPs used within Monash Health.
- To determine processes for monitoring and reviewing existing TCPs.
- To ensure that the operation of the Committee accords with the relevant policies and guidelines and any other legislative requirements that come to our attention.
- To ascertain and disseminate evidence of new TCPs (eg horizon scanning).
- To ascertain and disseminate synthesised evidence on use of TCPs as it is published.
- To provide advice to the Monash Health Therapeutics Committee and the Human Research Ethics Committees as requested and hold joint meetings as required in respect of human research projects including assessment of any credentialing, safety, clinical effectiveness and cost effectiveness issues as appropriate.

Reporting

The Technology and Clinical Practice Committee should operate within a reporting structure to ensure corporate and clinical governance.

- The TCPC will report to the Monash Health EMT.
- Monthly reports will be provided to the EMT on technologies that are considered for disinvestment.
- Six monthly reports to be provided to the EMT and the Victorian Department of Health detailing applications submitted, approved procedures, reviews of existing TCPs and monitoring of introduced/referred TCPs.

Business Rules

- Meetings shall be held once per calendar month or as required.
- The Chair, or in the Chair’s absence, a member nominated by the Executive Sponsor shall preside as Chair at every meeting of the Committee.
- If within thirty (30) minutes from the time appointed for the meeting a quorum (See quorum requirements above) is not present the meeting shall be dissolved.
- No business shall be transacted at any meeting unless a quorum is present at the time when the meeting proceeds to business.
- The Committee, where possible, shall reach consensus on applications under consideration. At any meeting a resolution put to the meeting shall be decided by consensus.
- Where voting becomes necessary the decision of the majority shall be carried. In an equality of votes on a show of hands, the Chair shall have the casting vote in addition to the vote to which he/she may be otherwise entitled.
- Every permanent member of the committee present in person shall have one vote.
- Co-opted members will not have a vote. The Program Director supporting the new TCP will not have voting rights.
- Where a Committee member abstains or dissents, this fact may, at the discretion of the member, be recorded in the minutes.

Appeals Process

Appeals against the decisions of the Committee will be directed to the Chief Executive.

Quality within the Committee

A quality framework for the committee will ensure proactive effective management. Various data will be collected to define the level of effectiveness of the committee. This data will include:

- The number of meetings attended by each committee member/delegate in relation to the number of meetings held.
- The number of quorums in relation to the number of meetings held.
- The meeting of reporting requirements.

APPENDIX 8. JOINT COMMITTEES TERMS OF REFERENCE

BACKGROUND

Any combination of the Executives of the following committees will be required for the assembly of a Joint Committee Meeting dependent on the requirements of the applications.

Technology/Clinical Practice Committee (TCPC): assesses application to introduce new technologies and clinical practices (TCPs). A new TCP is defined as a therapeutic intervention (including prostheses; implantable devices; high cost pharmaceuticals; medical, surgical or other clinical procedures) or diagnostic procedure that is considered by a reasonable body of clinical opinion to be significantly different from existing clinical practice.

***Therapeutics Committee*:** deals with all medication policies and issues.

Clinical Ethics Committee (CEC): provides advice to clinicians on problems of a clinical ethical nature. The CEC supports decision making by clinicians within an ethical framework. The CEC is established to receive enquiries relating to clinical ethical problems rather than to clinical competence, professional discipline or resource allocation.

Human Research Ethics Committee (HREC): reviews research projects involving Monash Health patients, employees or resources. The HREC ensures that they are sufficiently informed on all aspects of a research proposal, including its scientific and statistical validity, before deciding whether a proposal is both acceptable on ethical grounds and conforms with the National Statement.

ROLE

To establish a process that will facilitate decision-making for applications made to the TCPC, Therapeutics Committee, CEC or HREC where a joint assessment is required.

The role of each represented Committee will be in accordance with their individual Terms of Reference.

MEMBERSHIP

Each participating Committee will send representatives, at a minimum this should be the Committee Executive or their delegates (ie Committee Chair, Executive Officer and another representative).

The Chair of the Joint Committee Meeting will be that of the lead Committee responsible for the application.

RESPONSIBILITIES

The lead Committee's Secretariat will be responsible for

- Providing a Chair and minute taker for the meeting
- Determining data to be collected and reporting intervals required
- Maintaining a database of follow up data and adverse events of submitted and approved applications
- Determining processes for monitoring and reviewing applications
- Ensuring that the operation of the Joint Committee accords with the relevant policies and guidelines and any other legislative requirements that come to our attention

REPORTING

Joint Committee Meetings should operate within a reporting structure to ensure corporate and clinical governance.

The Secretariat responsible for applications reviewed by Joint Committees will ensure that

- Meetings of Joint Committees will report to the Monash Health Executive Management Team
- Minutes of the Joint Committee Meeting will be circulated to members of all appropriate committees

MEETING FREQUENCY

- Meetings shall be convened as required
- If within thirty (30) minutes from the time appointed for the meeting the accepted quorums for each represented committee is not present the meeting shall be dissolved.
- No business shall be transacted at any meeting unless the quorums are present at the time when the meeting proceeds to business.
- The Committee, where possible, shall reach consensus on issues relating to applications under consideration. At any meeting a resolution put to the meeting shall be decided on a show of hands.
- Where voting becomes necessary the decision of the majority shall be carried. In an equality of votes on a show of hands, the Chair shall have the casting vote in addition to the vote to which he or she may be otherwise entitled.
- Every member present in person shall have one vote on a show of hands.
- Where a Committee member abstains or dissents, this fact may, at the discretion of the member, be recorded in the minutes.

APPENDIX 9. DECISION SUMMARY

Meeting Date			
Application #			
Title of TCP			
☐ New TCP	☐ Substitute/replacement for existing	☐ Extended use of existing	☐
Conflict of Interest declaration			
Applicant ☐ Yes ☐ No			
Committee ☐ Yes ☐ No			
SAFETY			
☐ Safer than current practice	☐ Equivalent to current practice	☐ Less safe than current practice	
EFFECTIVENESS			
High quality evidence?			
Consistent, clinically important benefit?			
Applicable to Monash Health?	Yes		
COST			
CLINICAL FEASIBILITY			
Resource implications			
Credentialing and competency assurance undertaken			
ISSUES RELATED TO ACCESS & EQUITY AND LEGAL & ETHICAL IMPLICATIONS			

Final decision by the Monash Health Technology/Clinical Practice Committee	
☐	**Recommended:** Approved with no further need for assessment.
☐	**Restricted Recommendation – Audit:** Approval subject to implementation under audit conditions. Conditions are specific to the technology.
☐	**Restricted Recommendation – Clinical Trial:** Endorsed, however approval subject to implementation in clinical trial with Monash Health Human Research and Ethics Committee approval.
☐	**Restricted Approval – Operational Restrictions:** Endorsed, however financial or operational restrictions apply.
☐	Not Recommended

General Conditions

a. The Head of Department/Unit is required to notify the Secretariat of TCPC in the event of:
- Any change in protocol and the reason for that change together with an indication of ethical implications
- Adverse effects of the TCP and steps to deal with them
- Any unforeseen events

b. Adverse Events
- If a significant adverse event occurs the Head of Department/Unit must immediately notify the TCPC.

c. Consent
- Compliance with the Monash Health Consent Policy is mandatory.

- Written consent must be obtained for any treatment, investigation or procedure that involves a new technology or clinical practice

d. Data Collection

- All approved TCPs must be audited and data collected reported to the Committee. A generic "Outcome Spreadsheet" is available for use if required by the applicants. However if applicants want to use their own audit tool that is acceptable.

e. Reporting

- Reporting required at six monthly intervals; January – June and July – December; for a two year period.
- Reports should be forwarded to TCPC Secretariat. TCPC will forward reports to the Department of Health. A "Progress Report " template is provided for this purpose

f. Quality Assurance

- Collection of audit data constitutes a Quality Assurance (QA) activity. In general QA activities do not usually require Human Research Ethics Committee (HREC) approval, however in order to meet the requirements of many journal editorial boards an HREC letter acknowledging that the activity is not research and is correctly identified as QA is often required. At Monash Health, the TCPC has obtained generic approval from Monash Health HREC for this QA activity for applications approved by the TCPC so long as the data collected is confined to that described within the "Progress Report" template and the "Outcome Spreadsheet". Applicants are advised to complete the QA supplement (attached) and forward it to the TCPC secretariat which will then forward it to the HREC. The HREC will register your application as a Quality Assurance activity and provide you with a certificate. If the data being collected by you is beyond the scope of the templates or if your responses to the questions contained in the QA Supplement are in the affirmative, then the project may warrant further review by the HREC.

g. Review

- At the conclusion of the two year period the original application will be reviewed by the TCPC to determine if it should be considered standard practice.

Special Conditions

-

DECISION SUMMARY COMPLETED EXAMPLE

**Meeting Date**	Friday 9 May 2008		
**Application #**	08007 (follow-on application previously 07003)		
**Title of TCP**	"Arctic Front" Cryo-balloon pulmonary vein insertion		
☐ New TCP	☒ Substitute/replacement for existing	☐ Extended use of existing	☐ Other
Cryo-ablation is presented as a safer alternative to the current practice of radiofrequency ablation in patients with paroxysmal atrial fibrillation. This procedure was given restricted approval by the TCPC in 2007 for use in training/demonstration procedures at Southern Health. The current application is to address cost and operational issues prior to approval for ongoing use.			

**CONFLICT OF INTEREST DECLARATION**		
**Applicant**	☒ Yes ☐ No	No conflict of interest
**Committee**	☒ Yes ☐ No	Individual committee members declared potential conflicts of interest in relation to previous interaction with the applicant. The committee decided that these interactions would not act as conflicts of interest in the decision-making process.

**SAFETY**		
☒ Safer than current practice	☐ Equivalent to current practice	☐ Less safe than current practice
The risk of complications of the procedure is reduced. A world-wide survey of catheter ablation reported at least one major complication in 6% of patients. In a multi-centre prospective case series of 346 patients no major complications were identified with cryo-balloon ablation. The procedure is shorter, reducing the length of time under general anaesthetic.		

**EFFECTIVENESS**	
High quality evidence?	No comparative studies are available
Consistent, clinically important benefit?	Prospective case series suggest equivalent effectiveness to current practice
Applicable to Southern Health?	Yes
There is no high quality evidence regarding the clinical effectiveness of this procedure, however from the information available it appears to have similar effect to current practice, with a considerable reduction in major complications	

**COST**
No information is available on cost-effectiveness or cost-benefit. Cost savings are anticipated due to reduction in complication rates, shorter theatre time, shorter length of stay and reduced requirement for 3D mapping. The expected number of patients referred per annum (six) can be covered within the current Cardiology budget.

**CLINICAL FEASIBILITY**	
Resource implications	Adequate resources are available to perform these procedures
Credentialing and competency assurance undertaken	Cryo-ablation is undertaken using procedural techniques that are in current practice at MMC and the medical and nursing staff involved have the required expertise. There is some learning required in relation to the sequence of events, timing and team work related to the new procedure. The MMC team have already noticed a reduction in time taken for the procedure as they become more familiar with it. Dr Jeffrey Alison will be the only doctor undertaking the procedure.
This procedure is feasible at Southern Health: existing staff have appropriate training and expertise, no additional resources will be required and it can be achieved within current budgets	

**ISSUES RELATED TO ACCESS & EQUITY AND LEGAL & ETHICAL IMPLICATIONS**
All expected referrals can be treated within current systems and funding arrangements. Patient information will be provided prior to obtaining informed consent.<br>Data to be collected on all patients and reports provided to TCPC at six monthly intervals.

**Final decision by the Southern Health Technology/Clinical Practice Committee**	
☐	**Recommended:** Approved with no further need for assessment.
☒	**Restricted Recommendation – Audit:** Approval subject to implementation under audit conditions. Conditions are specific to the technology.
☐	**Restricted Recommendation – Clinical Trial:** Endorsed, however approval subject to implementation in clinical trial with Southern Health Human Research and Ethics Committee approval.
☐	**Restricted Approval – Operational Restrictions:** Endorsed, however financial or operational restrictions apply.
☐	**Not Recommended**

Conditions

- Data collection tool (spreadsheet/database) to be forwarded to TCPC
- Data to be collected on all patients and reports provided to TCPC at six monthly intervals
- Adverse events to be reported immediately to TGA and TCPC

APPENDIX 10: CERTIFICATE OF APPROVAL

**Date**	
**Application #**	
**Title of TCP**	
**Applicant/s**	
**Dept/Unit**	
**Head of Dept/Unit**	
**TCPC Meeting Date**	
**This application has been approved for the period**	Ongoing use or Limitation specified

General Conditions

The Head of Department/Unit is required to notify the Secretary of the Technology/Clinical Practice Committee (TCPC) in the event of:

1. Any change in protocol and the reason for that change together with an indication of ethical implications
2. Adverse effects of TCP and steps taken to deal with them
3. Any unforeseen events

If an adverse event occurs the Head of Department/Unit must immediately notify the Therapeutic Goods Administration in addition to the TCPC.

The Head of Department/Unit is required to complete and forward a progress report to the TCPC every six months unless otherwise specified.

Special Conditions

- Data collection tool (spreadsheet/database) to be forwarded to TCPC
-

**Person/s credentialed to perform approved TCP**	

Approved TCP to be performed for the following indications only

-

**Due date of first progress report**	

Please quote Application Number and Title for all correspondence

APPENDIX 11. INTRODUCTION OF A NEW TCP APPLICATION FORM

INTRODUCTION OF A NEW TECHNOLOGY/CLINICAL PRACTICE (TCP)

APPLICATION FORM

How to complete this form

- Please answer every question
- To complete written answers, insert cursor in grey box and commence typing
- To select answer from available options, double click on the appropriate box and select 'checked'

Submissions

- All applications should be submitted electronically to TCPC@monashhealth.org
- For submission deadlines please see [Meeting Dates]()

SECTION 1: SUMMARY OF INFORMATION							
Title of Technology/Clinical Practice (TCP)							
Program		Department/Unit					
Principal clinical discipline/service (eg Cardiology, Neurosurgery)							
Reason for Application (check all that apply)							
☐ Safety		☐ Effectiveness		☐ Cost effectiveness			
Number of cases planned for proposed TCP							
CONTACT DETAILS							
Lead Contact Person							
Name				Title		Position	
Phone		Fax		Email			
**Referees Details** (Please specify two referees from other Victorian health services, interstate or overseas with experience in the proposed TCP for external assessment)							
Referee 1							
Name				Title		Position	
Phone		Fax		Email			
Referee 2							
Name				Title		Position	
Phone		Fax		Email			
APPLICANT'S SIGNATURES							
Name				Signature		Date	
Name				Signature		Date	
ENDORSEMENT BY HEAD OF DEPARTMENT/UNIT							
I support this application and agree to provide Progress Reports to the TCP Committee as required							
Name				Signature		Date	
ENDORSEMENT BY PROGRAM DIRECTOR							
Name				Signature		Date	

Please insert electronic signatures or print **<u>this page only</u>**, sign and fax to "For TCPC" on 9594 6030

SECTION 2: OVERVIEW OF TECHNOLOGY/CLINICAL PRACTICE (TCP)			
**1. Description of TCP** (Provide a brief plain language statement describing the proposed TCP)			
**2.** Classification of TCP (check appropriate box)			
☐ A new TCP		☐ Substitute or replacement for an existing TCP	
☐ Extended use of an existing TCP		☐ Other (specify)	
**3. Category of TCP** (check appropriate box)			
☐ Prosthesis	☐ Implantable device	☐ Diagnostic technique	
☐ Medical procedure	☐ Surgical procedure	☐ High cost pharmaceutical	
☐ Other (specify)			
**4.** Introducing the proposed TCP – collaboration with other health services			
Would the TCP be available to patients referred from other health services?		☐ YES ☐ NO	
**5. Clinical Setting** (Specify whether the proposed TCP is to be used in the following settings)			
☐ Inpatient ☐ Outpatient ☐ Mix of inpatient and outpatients		% inpatients	% outpatients
☐ Other (specify)			
**6. Use of proposed TCP elsewhere** (Describe here the use of the proposed TCP elsewhere, both nationally and internationally)			
**7. Coding** (Specify relevant DRG, ICD procedural/diagnostic codes and/or other coding classifications)<br>Please contact Susan Peel in Health Information Services (9594 1382) for correct coding information			
**8.** Additional information for High Cost Pharmaceuticals			
Is the submission for a High Cost Pharmaceutical?	☐ YES ☐ NO	If YES please provide the following	
Generic Name		Trade Name	
Dosage form		Dosage strength	
Pack/vial/bottle size		Normal dosage schedule	
Normal duration of treatment			
Restrictions recommended			
Specify line therapy (ie first line, second line, etc)			
**9.** Additional information for Radiation Safety			
Does this TCP have a radiation source?		☐ YES ☐ NO	
If Yes, does it comply with the Monash Health licensing agreement?<br>Please contact the Radiation Safety Officer (8541 6407) for radiation safety information		☐ YES ☐ NO	
**10.** Care Continuum / Pathway<br>The care continuum represents the patient journey through related episodes of care to treat a specific disease/clinical problem and incorporates the following:<br>▪ Care from primary through to quaternary providers<br>▪ Care from medical, allied health and nursing personnel<br>▪ Inpatient and non inpatient care<br>▪ Different types and quanta of care at different stages of the clinical problem<br>▪ Various treatment settings<br>Please detail the care continuum and pathway for the patients proposed to receive the TCP taking into account, but not limited to, the above			
SECTION 3: CLINICAL NEED			
**11.** Clinical indication/disease/condition			
**a.** Specify the clinical indication/disease/condition that the proposed TCP will treat			
**b.** Provide a brief description of the clinical indication/disease/condition and its clinical progression and prognosis			
**c.** Specify whether the indication/disease/condition is severe, progressive and expected to lead to premature death			

**d.**	Provide details concerning the incidence and prevalence of the clinical indication/disease/condition in Australia			
**12.**	Patient population(s)			
**a.**	What are the demographic characteristics of the patient population(s) with the clinical indication/disease/condition (eg age range, median and mean; gender; ethnicity; occupation; socio-economic status)?			
**b.**	What is/are the subgroup(s) of the patient population(s) that will benefit from the proposed TCP?			
**c.**	What factors are taken into account when considering patient selection for use of the proposed TCP?			
**d.**	Specify the number of adult patients who will receive the proposed TCP per annum			
**e.**	Specify the number of paediatric patients who will receive the proposed TCP per annum			
**f.**	If this number is expected to increase over time and/or have a cumulative component due to ongoing follow-up, please specify the predicted numbers of new and follow-up patients by year for 5 years			
**13.**	Comparison with existing approach(es) to clinical intervention			
**a.**	What existing and approved TCP(s) is/are used for this clinical indication/disease/condition?			
**b.**	Describe how the proposed TCP differs from those in current practice eg<br>▪ Significant clinical advantages over existing treatment<br>▪ No worse than existing treatment in terms of effectiveness/toxicity<br>▪ Less effective that the existing treatment, but has less toxicity			
**14.**	Opportunities for disinvestment			
**a.**	Identify and detail any anticipated disinvestment opportunities that implementing the new TCP will, or is likely to, have on existing clinical technology or practice.			
**b.**	How do you plan to measure this impact?			
**15.**	Health outcomes			
**a.**	What are the health outcomes that will be achieved by the proposed TCP?			
**b.**	How will these be measured?			
**c.**	Over what time frame?			
SECTION 4: EVIDENCE OF SAFETY, EFFICACY AND CLINICAL EFFECTIVENESS<br>The CCE ***'Finding the Evidence'*** guide will assist you in completion of this section.<br>If you require additional assistance contact CCE (9594 7553)				
**16.**	Regulatory approval			
**a.**	Provide documentary evidence of approval and approval date for the proposed TCP for use in Australia for the identified clinical indication(s) by the Therapeutic Goods Administration			
**b.**	If a High Cost Pharmaceutical, please provide the following information			
	▪ Has this been submitted to the Monash Health Therapeutics Committee<br>If Yes, please check appropriate box below	☐ YES	☐ NO	
	☐ Application in progress	☐ Application approved	☐ Application rejected	
	▪ Has manufacturer/distributor sought listing on the PBS (section 85 or section 100), Commonwealth Chemotherapy Pharmaceutical Access Program of Highly Specialised Drugs Program for the identified indication(s)?			
	☐ YES ☐ NO Date	If YES, please attach documentary evidence of PBAC recommendations		
	▪ List other indications for this drug that are funded by existing programs			
**c.**	The TCP you are proposing may have already been reviewed and a decision made regarding its use in Australia. MSAC Health Technology Assessments are available at www.msac.gov.au .<br>**Is there a current MSAC Review available for this TCP?**	☐ YES	☐ NO	
	If YES, please provide documentary evidence of the MSAC Recommendations			
	If NO, is there one currently under review?			
	☐ YES	Please contact Dr Claire Harris on 9594 7576 **before proceeding**	☐ NO	Move on to Q17
**17.**	Evidence of safety			
**a.**	Provide evidence of safety associated with the use of the TCP for the proposed indication(s)			

	(Please provide source/references)
**b.**	List nature and incidence of side effects, contra-indications, cautions, warnings and adverse effects with use of the TCP for the proposed indication(s) (Please provide source/references)
**c.**	What are the main differences in the indications, contra-indications, cautions, warnings and adverse effects between the proposed TCP and existing treatments? (Please provide source/references)
**18.**	Evidence of efficacy and clinical effectiveness<br>Evidence of efficacy and clinical effectiveness must exist for the proposed TCP. Please summarise the best available evidence, outlining key aspects for clinical effectiveness of the TCP for the defined clinical problem(s).<br>**Please note:** if the TCP is a diagnostic test, you must provide information about the comparative effectiveness against the current gold standard (ie provide information about sensitivity and specificity of the proposed test).<br>Please complete the Appendix and summarise the evidence in the tables below.
**19.**	Clinical guidance/clinical practice guidelines/other<br>Specify briefly whether Clinical Guidance, Clinical Practice Guidelines, WHO Classifications or other similar exist for the proposed TCP in the defined clinical problem.<br>Please complete the relevant section on guideline searches in the Appendix and summarise below.
**20.**	Health service assessment
**a.**	What assessment has occurred within Monash Health for the proposed TCP? You <u>must</u> provide details of any assessment and outcomes.
**b.**	Please provide details of health service/hospital ethical considerations regarding the proposed TCP.
SECTION 5: EVIDENCE OF COST EFFECTIVENESS	
**21.**	Evidence of cost effectiveness<br>Evidence of cost effectiveness should support submissions. Applicants may wish to report results of their own cost-effectiveness/cost-utility study (usually undertaken alongside a clinical trial) or report results for a model of costs and effectiveness based on data from the published literature.<br>Economic evaluations can be identified by searching The Cochrane Library. The CCE '***Finding the Evidence'*** guide will assist you with this.
**22.**	Health service assessment of cost effectiveness
**a.**	What assessment of cost effectiveness has occurred within Monash Health, and by whom or what committee or group, for the proposed TCP?
**b.**	Please provide documentary details and outcomes.

Evidence Summary 1 – use this table for evidence related to effectiveness of a therapy or the impact of a diagnostic test

Study	Level of evidence	Risk of bias	Direction of effect	Size of effect	Precision or Statistical significance	Similar patient population	Similar health systems
Smith 2007	I, II, III-1	Low/med/high	Favours intervention or favours control	Outcome description and point estimate	95% confidence intervals or p-value	Yes/No/Unclear	Yes/No/Unclear
Summary	High quality evidence?		Consistent, clinically important benefit?			Applicable to Monash Health?	
Yes/No/Unclear							
Comment							

Evidence Summary 2 – use this table for evidence related to the accuracy of a diagnostic test

Study	Level of evidence	Risk of bias	Accuracy of new test		Similar patient population	Similar health systems
			Sensitivity	Specificity		
Smith 2007	I, II, III-1	Low/med/high	%	%	Yes/No/Unclear	Yes/No/Unclear
Summary	High quality evidence?		Consistent, high accuracy sensitivity and specificity?		Applicable to Monash Health?	
Yes/No/Unclear						
Comment						

SECTION 6: CLINICAL FEASIBILITY<br>THIS SECTION TO BE COMPLETED IN CONSULTATION WITH CLINICAL INFORMATION MANAGEMENT<br>Contact Anthony Gust (9594 4017)						
**23.** Bed utilisation						
**a.** Specify whether use of the proposed TCP will require patients to be managed in						
☐	Intensive Care	☐	Multi day beds	☐	Same day beds	
**b.** What is the average length of stay per annum for patients receiving the proposed TCP?						
**c.** What bed numbers are required per annum?						
**d.** Will bed utilisation be increased or decreased, and by how much, with the proposed TCP?						
☐ Increased by			☐ Decreased by			
**e.** Will this occur within existing capacity?	☐ YES	☐ NO	If NO, detail the proposed solution			
**f.** How will introduction of the new TCP effect demand management and access to existing elective/emergency patients?						
**24.** Clinical personnel and expertise						
**a.** Please specify the type of clinical personnel required to implement the proposed TCP						
**b.** Detail the existing clinical personnel and expertise available to implement the proposed TCP						
**c.** Are additional clinical personnel and expertise required to implement the proposed TCP?	☐ YES	☐ NO	If YES, please specify			
**25.** Operator competency						
**a.** Specify what credentialing and competency assurance is needed to ensure safe implementation of the proposed TCP						
**b.** Has this been undertaken?	☐ YES	☐ NO	If NO, how and when will this occur?			
**26.** Associated service utilisation						
**a.** Specify all other services that will be utilised for the proposed TCP						
☐ Intensive Care	☐ Operating theatre	☐ Imaging				
☐ Pathology	☐ Outpatients	☐ Other (specify)				
**b.** Are these available within existing capacity?	☐ YES	☐ NO	If NO, why not?			
**c.** If additional services are required to implement the proposed TCP please specify what these are and how you propose to source them<br>Consideration of the following elements should inform the proposed costs for clinical and other services concerning Pre-admission assessment, Inpatient care and Post-discharge care (eg FTE and other associated costs)						

		Pre-admission assessment	Inpatient care	Post-discharge care and follow up
☐	Specialist Medical Practitioner			
☐	Allied Health by type			
☐	Pharmacy			
☐	Theatre (Surg, Anaesth, Other)			
☐	Intensive Care			
☐	Imaging			
☐	Pathology			
☐	Special consumables			
☐	Dietary supplements			
☐	Outpatient services			
☐	Organisational overheads			
☐	Other			
**27.** Future service impacts				

**a.** Are there emerging trends in this TCP that may have substantive future impacts on services?	☐ YES	☐ NO	If YES, please describe briefly

28 Infrastructure Needs

**a.** Is new equipment being used in the introduction of this TCP	☐ YES	☐ NO
**b.** If yes have you checked that the new equipment is compatible with existing infrastructure?	☐ YES	☐ NO

Please check any infrastructure compatibility issues with Director Health Technology Services (85416404)

SECTION 7: GOVERNANCE

29. Describe the clinical governance arrangements and processes overseeing the implementation of the TCP

(This submission must demonstrate, where appropriate, that existing appropriate governance structures have considered the proposed TCP eg ethics, research, Therapeutics Committee)

30. Patient information sheet

Patient information sheets are a requirement to inform potential recipients prior to being treated with the new TCP.

Monash Health has a 'Patient Information Sheet Template' that will help you to develop patient information

To access the template go to the "What resources are available to help with the technical aspect of the application process" after you click on the hyperlink above.

a. Is a patient information sheet attached?	☐ YES	☐ NO	If No, please explain why not

b. Have specific risks arising from the proposed TCP been considered and will patients be explicitly informed about these?

31. Monitoring and Evaluation

a. Specify how you will monitor the TCP once it is introduced into the clinical setting

Please comment on each of the following elements that might be considered as part of the monitoring process following the introduction of a TCP within the clinical setting

Learning curve operator(s)	
Credentialing	
Experience	
Quality Plan	
Stopping rule	
Other	

b. Specify an evaluation protocol for the TCP including performance indicators and defined time points

SECTION 8: ESTIMATED FINANCIAL IMPACT

SECTION 8.1 EXISTING COSTS FOR CURRENT PRACTICE
THIS SECTION TO BE COMPLETED IN CONSULTATION WITH CLINICAL INFORMATION MANAGEMENT
Contact Anthony Gust (9594 4017)

32. Existing costs for current practice

To enable CIM to identify the required information please document

- Any specific clinics/wards relevant to each setting (Pre-admission assessment, Inpatient care and Post-discharge care)
- Details of any of the following elements relevant to each setting (eg FTE and any other associated costs)

Specialist Medical Practitioner	Intensive Care	Dietary supplements
Allied Health by type	Imaging	Outpatient services
Pharmacy	Pathology	Organisational overheads
Theatre (Surg, Anaesth, Other)	Special Consumables	Other

Pre-admission assessment	Inpatient care	Post-discharge care and follow up

Provide details of existing costs for <u>current clinical practice</u> in this patient population. To be completed by CIM

33. Existing revenue for current practice

i. Provide details of revenue and its sources for the care continuum for <u>current clinical practice and treatment</u> in this patient population

Source	
WIES	To be completed by CIM
VACS medical	To be completed by CIM
VACS allied health	To be completed by CIM
Specified grants	
Alternative funding mechanisms eg Highly Specialised Drugs Sect 100	
Other	
SECTION 8.2 PROJECTED COSTS FOR PROPOSED TCP<br>THIS SECTION TO BE COMPLETED IN CONSULTATION WITH PROGRAM BUSINESS MANAGER<br>Assistance available from CIM (Anthony Gust 9594 4017) and SH Finance Dept (Basil Ireland 9594 2832)	
**34.** Projected costs for proposed TCP	
**i.** If the proposed TCP is a prosthesis, implantable device, high cost pharmaceutical or diagnostic test	
What is the unit cost?	What is the average number of units administered/used per case?
**ii.** Indicate if additional costs are required to implement the TCP not covered by usual revenue sources	
Staffing and salaries (specify each type and number of clinicians by session/hours/FTE as appropriate)	
Administration (staffing and salaries by FTE)	
Staff/salary overheads (provide breakdown)	
As appropriate for (i) Pre-admission assessment, (ii) Inpatient care and (iii) Post-discharge follow-up, for each clinical and other service specify how the costs are derived	

	Pre-admission assessment	Inpatient care	Post-discharge care and follow-up
Specialist Medical Practitioner			
Allied Health by type			
Pharmacy			
Theatre (Surg, Anaesth, Other)			
Intensive Care			
Imaging			
Pathology			
Special consumables			
Dietary supplements			
Outpatient services			
Organisational overheads			
Other			

**iii.** What are the inpatient and outpatients costs per case?
**iv.** What is the total cost of the proposed TCP per case?
**v.** Specify the source of costing data for each element
**35. Additional recurrent budget requirement** (If applicable detail and justify recurrent budget requirements per case)
**36. One-off establishment costs** (If applicable specify type, amount and reasons eg specialist equipment and training)
SECTION 9: IMPLEMENTATION OF THE TCP
**37.** Implementing the proposed technology/clinical practice<br>To inform this process, please provide details of your plan for implementing the TCP, including
**1.** Milestones
**2.** Timeframes
▪ Management of the implementation (especially if the TCP will be implemented across multiple sites)

SECTION 10: DECLARATION OF POTENTIAL CONFLICT OF INTEREST		
This Declaration is to ensure all potential conflicts of interest are addressed in a rigorous and transparent way that accords with the requirements of the *National Health and Medical Research Council Act 1992*.		
Title of Technology/Clinical Practice (TCP)		
I hereby declare that: (check whichever is applicable)		
☐	I have no interests to declare which may relate to the proposed Technology/Clinical Practice.	
☐	I have listed below all interests which I have that may relate to the proposed Technology/Clinical Practice.	
Please provide an explanation of the implications of any conflict of interest and why this application should be accepted regardless		
Category		Explanation
☐	Paid positions including invited lectures and membership of advisory panels, working groups etc for which honoraria or considerations in kind were received	
☐	Shares and other commercial dealings	
☐	Financial or other sponsorship of research	
☐	Significant subsidies, whether partial or complete, for any travel, accommodation or entertainment	
☐	Gifts of any kind (greater than $50 in value)	
☐	Any other relevant activity	
Please check **both** of the statements below to acknowledge and accept the requirements of the Monash Health TCPC		
☐	I acknowledge that I am required to disclose the nature of my interests for the proposed Technology/Clinical Practice at the time of the meeting of the Monash Health TCPC. If a matter is to be decided before I am able to disclose my interest at the meeting, then I am obliged to disclose the nature of those interests as soon as possible.	
☐	I accept that if I acquire an interest that could conflict with the proposed Technology/Clinical Practice during the course of its implementation, I will disclose that by correspondence with the Chair of the Monash Health TCPC as soon as possible after the relevant facts have come to my knowledge.	

APPLICANT'S SIGNATURE					
Name		Signature		Date	
Name		Signature		Date	

Please insert electronic signatures or print **<u>this page only</u>**, sign and fax to CCE on 9594 7554

SECTION 11: APPENDIX
Please complete the following tables for evidence on the proposed TCP. Use the CCE '[Finding the Evidence]' guide to understand how to complete each section. ***For further assistance please contact CCE (9594 7575)***
1. SEARCH

PICO	PICO Terms	Alternative Terms
Patient/Population		
Intervention/Indicator		
Comparison/Control		
Outcomes		

The Cochrane Library		
AND/OR	Search Terms	eg Title, Abstract or Keyword

Pubmed Clinical Queries – Search String
2. SEARCH RESULTS

Databases	Searched Y/N	No. of items returned	No. of relevant articles
Systematic Reviews/HTAs			
MSAC			
The Cochrane Library – Systematic Reviews			
PubMed Clinical Queries – Systematic Reviews			
Other HTA Websites			
Clinical Trials			
The Cochrane Library – Clinical Trials			
PubMed Clinical Queries – Clinical Trials			

Guideline websites	Searched Y/N	No. of relevant guidelines
National Health and Medical Research Council (NHMRC)		
National Institute for Health and Clinical Excellence UK (NICE)		
New Zealand Guideline Group (NZGG)		
Scottish Intercollegiate Guidelines Network (SIGN)		
Joanna Briggs Institute		
Guidelines International Network *(CCE will search for you)*		
Guidelines Advisory Committee		
National Guideline Clearinghouse US (NGC)		
TRIP Database		
Google		
Other		

3. CRITICAL APPRAISAL
The following templates will assist you in appraising the relevant articles you identified in the above tables. You will need to copy additional templates if you have more than one publication to appraise.

SYSTEMATIC REVIEW						
Reference						
CHARACTERISTICS						
Study Type	Population (total)	Setting	Patients	Intervention	Comparison	Outcomes
QUALITY						

Questions	Yes/No	Explanation
Did the authors declare any conflicts of interest?<br>(eg link to the manufacturer/received funding from parties with vested interests)		
Does the study have a focused research question?		
Does the study have specified inclusion/exclusion criteria?		
Does the study document a comprehensive search strategy?		
Were reviewers blind to authors, institutions and affiliations?		
Was the validity of included trials appraised?		
Was the homogeneity between included studies assessed?		
Does the study present a summary of the main results?		
Were the strengths and limitations of included studies discussed?		
Other Comments		

RESULTS	
Effectiveness	
Safety	
Conclusion	

RANDOMISED CONTROLLED TRIAL						
Reference						
CHARACTERISTICS						
Study Type	Population (total)	Setting	Patients	Intervention	Comparison	Outcomes
QUALITY						

Questions	Yes/No	Explanation
Did the authors declare any conflicts of interest?<br>(eg link to the manufacturer/received funding from parties with vested interests)		
Does the study have specified inclusion/exclusion criteria?		
Does the study have an adequate method of randomisation?		
Were groups similar at baseline?		
Was allocation to treatment group concealed?		
Were patients/investigators/assessors blind to treatments?		
Was there sufficient duration to follow-up?		
Was there a minimal portion of participants lost to follow up?		
Were outcomes assessed objectively and independently?		
Were all patients in their respective treatment groups analysed together, regardless of whether or not they completed or received treatment? (Intention-To-Treat analysis)		
Other Comments		
RESULTS		
Effectiveness		
Safety		
Conclusion		

COHORT STUDY						
Reference						
CHARACTERISTICS						
Study Type	Population (total)	Setting	Patients	Intervention	Comparison	Outcomes
QUALITY						
Questions			Yes/No	Explanation		
Did the authors declare any conflicts of interest?<br>(eg link to the manufacturer/received funding from parties with vested interests)						

Does the study have specified inclusion/exclusion criteria?		
Were groups similar at baseline?		
Were outcomes assessed blindly with respect to the exposure?		
Was there sufficient duration to follow-up?		
Was there a minimal portion of participants lost to follow up?		
Were outcomes assessed objectively and independently?		
Were all selected subjects included in the analysis of results?		
Other Comments		
RESULTS		
Effectiveness		
Safety		
Conclusion		

CASE-CONTROL						
Reference						
CHARACTERISTICS						
Study Type	Population (total)	Setting	Patients	Intervention	Comparison	Outcomes
QUALITY						

Questions	Yes/No	Explanation
Did the authors declare any conflicts of interest?<br>(eg link to the manufacturer/received funding from parties with vested interests)		
Does the study have specified inclusion/exclusion criteria?		
Does the study provide an explicit definition of cases?		
Were the control participants selected from the source population?		
Are the patient groups comparable with respect to confounders?		
Were outcomes assessed blindly with respect to disease status?		
Was there sufficient duration to follow-up?		
Were outcomes assessed objectively and independently?		

Were all selected subjects included in the analysis of results?		
Were cases and controls assessed in the same way?		
Other Comments		
RESULTS		
Effectiveness		
Safety		
Conclusion		

CASE SERIES						
Reference						
CHARACTERISTICS						
Study Type	Population (total)	Setting	Patients	Intervention	Comparison	Outcomes
QUALITY						
Questions	Yes/No	Explanation				
Did the authors declare any conflicts of interest?<br>(eg link to the manufacturer/received funding from parties with vested interests)						
Does the study have specified inclusion/exclusion criteria?						
Does the study provide an explicit description of study subjects?						
Was there sufficient duration to follow-up?						
Were outcomes assessed objectively and independently?						
Were all selected subjects included in the analysis of results?						
Other Comments						
RESULTS						
Effectiveness						
Safety						
Conclusion						

<table>
<tr><td colspan="7">DIAGNOSTIC TEST</td></tr>
<tr><td>Reference</td><td colspan="6"></td></tr>
<tr><td colspan="7">CHARACTERISTICS</td></tr>
<tr><td>Study Type</td><td>Population (total)</td><td>Setting</td><td>Patients</td><td>Intervention</td><td>Comparison</td><td>Outcomes</td></tr>
<tr><td></td><td></td><td></td><td></td><td></td><td></td><td></td></tr>
<tr><td colspan="7">QUALITY</td></tr>
<tr><td colspan="3">Questions</td><td>Yes/No</td><td colspan="3">Explanation</td></tr>
<tr><td colspan="3">Did the authors declare any conflicts of interest?
(eg link to the manufacturer/received funding from parties with vested interests)</td><td></td><td colspan="3"></td></tr>
<tr><td colspan="3">Does the study have specified inclusion/exclusion criteria?</td><td></td><td colspan="3"></td></tr>
<tr><td colspan="3">Is there an explicit description of study subjects?</td><td></td><td colspan="3"></td></tr>
<tr><td colspan="3">Is there an appropriate spectrum of consecutive patients who would normally be tested for the disorder of interest and whose disease status is not known?</td><td></td><td colspan="3"></td></tr>
<tr><td colspan="3">Was an appropriate ‘gold standard’ reference test used?</td><td></td><td colspan="3"></td></tr>
<tr><td colspan="3">Were all participants assessed with both study test and reference standard test?</td><td></td><td colspan="3"></td></tr>
<tr><td colspan="3">Was the assessment of test outcomes independent?</td><td></td><td colspan="3"></td></tr>
<tr><td colspan="3">Were assessors blind to the results of the other test?</td><td></td><td colspan="3"></td></tr>
<tr><td colspan="3">Was both sensitivity and specificity, or number of true positive, false positives, true negatives and false negatives reported?</td><td></td><td colspan="3"></td></tr>
<tr><td colspan="4">Other Comments</td><td colspan="3"></td></tr>
<tr><td colspan="7">RESULTS</td></tr>
<tr><td>Effectiveness</td><td colspan="6"></td></tr>
<tr><td>Safety</td><td colspan="6"></td></tr>
<tr><td>Conclusion</td><td colspan="6"></td></tr>
</table>

GUIDELINE						
TITLE						
AUTHOR						
PUBLISHER						
FUNDER						
LINK						
AIM						
CONTENTS						
QUALITY						
AGREE DOMAINS	SCORES			COMMENTS		
	Reviewer 1	Reviewer 2	%			
Scope and purpose	/12	/12				
Stakeholder involvement	/16	/16				
Rigour of development	/28	/28				
Clarity and presentation	/16	/16				
Applicability	/12	/12				
Editorial Independence	/8	/8				
RELEVENCE						
Source			Setting			
Developers			Target Audience			
SUMMARY						

APPENDIX 12. REVISED APPLICATION PROCESS FOR NEW TCPs

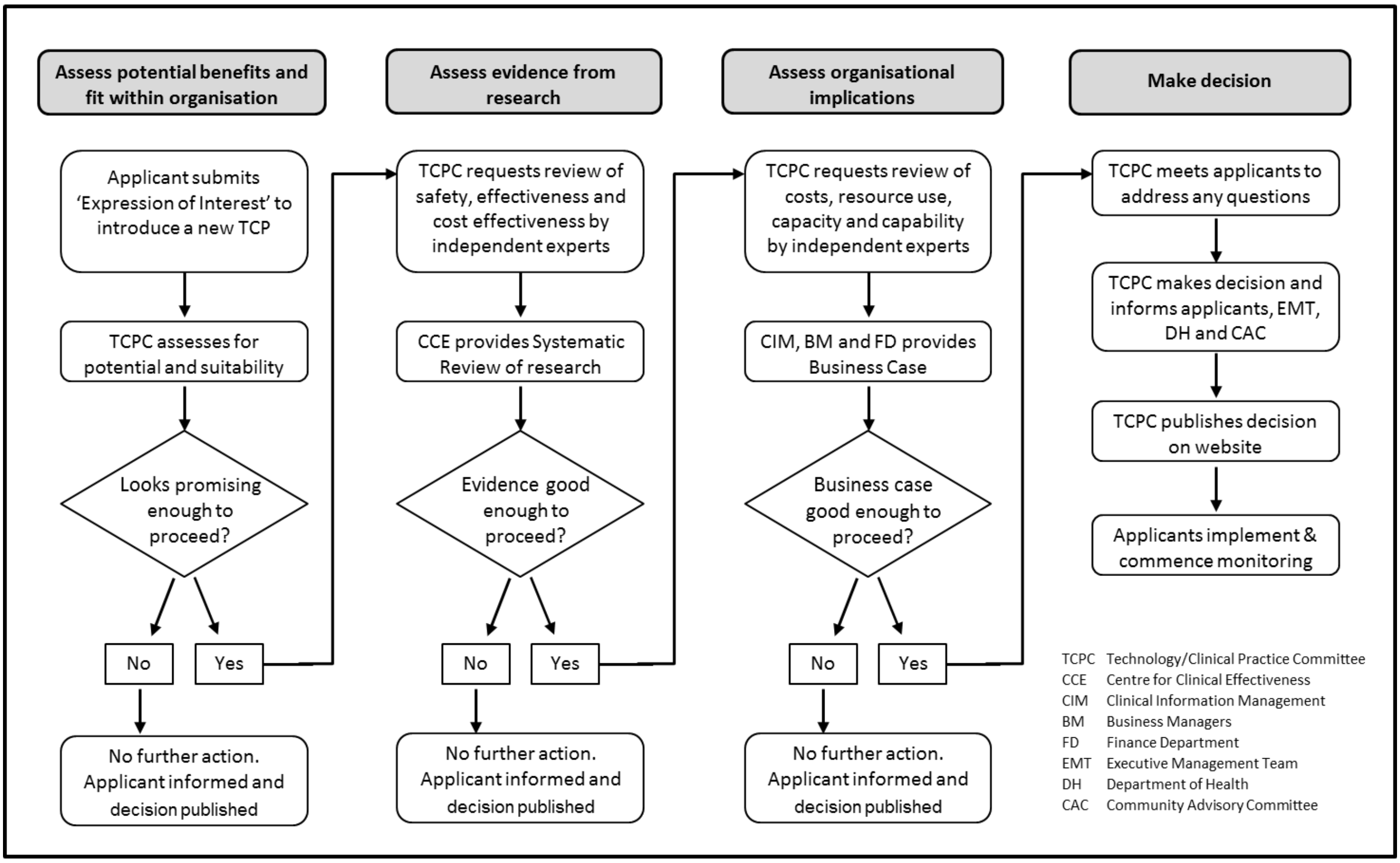

APPENDIX 13. INTRODUCTION OF A NEW TCP EXPRESSION OF INTEREST FORM

APPLICATION FORM

To reduce the burden on applicants and to ensure that the details provided for decision-making are accurate, Monash Health staff with appropriate expertise will be responsible for obtaining information regarding evidence, coding, costing, etc. The applicant will be invited to provide clinical input to inform each of these steps.

How to complete this form

- Please answer every question
- To complete written answers, insert cursor in grey box and commence typing
- To select answer from available options, double click on the appropriate box and select 'checked'

Submissions

- All applications should be submitted electronically to TCPC@monashhealth.org
- For submission deadlines please see Meeting Dates

SECTION 1: CONTACT DETAILS						
Title of technology or clinical practice (TCP)						
**Reason for application** (please select all that apply)						
☐ Safety	☐	Effectiveness	☐ Cost effectiveness			
Department/Unit/Discipline/Service						
Head of Department/Unit/Discipline/Service						
Program						
Program Director						
☐ I declare that the Head of Department/Unit/Discipline/Service has received and approved a copy of this completed application						Date
☐ I declare that the Program Director has received and approved a copy of this completed application						Date
Applicant						
Name			Title		Position	
Phone		Fax		Email		

SECTION 2: OVERVIEW					
**28. Description of TCP** (Provide a brief plain language statement describing the proposed TCP)					
**29. Classification of TCP** (please select appropriate category					
☐	A new TCP	☐	Substitute or replacement for existing TCP		
☐	Extended use of existing TCP	☐	Other (please specify)		
**30. Category of TCP** (please select appropriate category)					
☐	Diagnostic technique	☐	Prosthesis	☐	Nursing procedure/practice
☐	High cost pharmaceutical	☐	Allied Health procedure/practice	☐	Surgical procedure/practice
☐	Implantable device	☐	Medical procedure/practice	☐	Other (please specify)
**31.** Regulatory approval					
**a.** Monash Health requires approval from the Therapeutic Goods Administration (TGA) for introduction of new TCPs. Please provide one of the following					
☐	Australian Register of Therapeutic Goods (ARTG) certificate number				
☐	Special Access Scheme approval number				
☐	TGA has not approved this TCP → Please contact the TCPC Executive Officer on 9594 7575 or TCPC@monashhealth.org **before proceeding** with this application				
**b.** The proposed TCP may have already been reviewed by the Medical Services Advisory Committee (MSAC) and a decision made regarding its use in Australia. MSAC Health Technology Assessments available at www.msac.gov.au<br>Please note whether an MSAC review has been undertaken.					
☐	There is no MSAC review for this TCP				

☐	An MSAC review is currently underway → Please contact the TCPC Executive Officer on 9594 7575 or TCPC@monashhealth.org **before proceeding**
☐	An MSAC review for this TCP is available. Please note the MSAC recommendation(s) and ministerial decision

32. Use of proposed TCP elsewhere. Please describe the use of the proposed TCP elsewhere, both nationally and internationally.

SECTION 3: CLINICAL NEED

33. Clinical indication/disease/condition

a. Please describe the clinical progression and prognosis, incidence and prevalence in Australia, patient demographics (including subgroups, mix of adult and paediatric patients, etc).

b. Please describe the inpatient versus outpatient mix, expected annual number of patients, potential for patient numbers to increase over time.

34. Comparison with current Monash Health practice

Please describe current practice at Monash Health for these patients and outline the likely benefits and any potential risks or harms from changing to the proposed TCP.

35. Comparison with other alternatives

Please describe all other available alternatives and outline the reason the proposed TCP is preferred over these.

36. Patient health outcomes

Please describe the anticipated change in patient health outcomes, how they will be measured and in what timeframe these changes will occur.

37. Health service outcomes

Please describe the anticipated change in health service outcomes, how they will be measured and in what timeframe these changes will occur.

SECTION 4: EVIDENCE OF SAFETY, EFFICACY AND CLINICAL EFFECTIVENESS

The CCE ***'Finding the Evidence'*** guide will assist you in completion of this section.

38. Evidence of safety, efficacy and clinical effectiveness

For a new TCP to be introduced as standard practice at Monash Health applicants must establish that it is safer, more effective and/or more cost-effective than current practice based on good quality evidence. Suitable evidence would be:

- an appropriate cohort of sufficient size to demonstrate increased safety
- at least one randomised controlled trial to demonstrate effectiveness
- measures of diagnostic accuracy (sensitivity, specificity, PPV, NPV) for diagnostic tests
- either published information or good quality analysis of local data for cost-effectiveness

If these minimum levels of evidence are not available please state your rationale for accepting lower level studies as evidence.

The Monash Health TCPC encourages clinicians to investigate promising new procedures within a research framework to gather further evidence prior to application for introduction as standard practice.

a. Existing synthesised evidence

A rigorous synthesis of the available evidence (eg systematic review or evidence based guideline) is the best information to support an application to change practice. Please provide details of a search for systematic reviews (SRs) in the Cochrane Library (page 14 of the ***'Finding the Evidence'*** guide provides help if required).

Search terms used

SRs identified

If there is no synthesised evidence available, the TCPC may request that a systematic review is undertaken by the Centre for Clinical Effectiveness to inform the decision-making process.

b. Summary of evidence

Please summarise the available evidence of safety, effectiveness and cost effectiveness associated with the use of the TCP for the proposed indication(s). Include nature and incidence of side effects, contraindications, cautions, etc and how these differ from existing treatments. Please provide references.

SECTION 5: FINANCIAL COSTS and RESOURCE USE
**39.** Establishment costs
Please provide details of all establishment costs (eg equipment, capital works, etc) and how these costs will be met.
**40.** Ongoing costs
Please provide details of all ongoing costs (eg consumables, salaries, pharmacy, diagnostic imaging, pathology, organisational overheads, etc) and how these costs will be met.
**41.** Organisational capacity
Please provide details of changes to resource use and the outcomes of discussions with the areas affected (eg operating suite, intensive care, outpatients, allied health, etc).
**42.** Opportunities for disinvestment
Please provide details of any anticipated disinvestment opportunities that implementing the new TCP will, or is likely to, have on existing clinical technology or practice (ie activities that can be ceased or reduced as a result of the change). Please include how they will be measured and in what timeframe the changes will occur.

SECTION 6: DECLARATION OF POTENTIAL CONFLICT OF INTEREST		
This Declaration is to ensure all potential conflicts of interest are addressed in a rigorous and transparent way that accords with the requirements of the *National Health and Medical Research Council Act 1992*.		
Title of Technology/Clinical Practice (TCP)		
I hereby declare that: (check whichever is applicable)		
☐	Members of my Department/Unit have no interests to declare which may relate to the proposed TCP	
☐	Members of my Department/Unit have listed below all interests which they have that may relate to the proposed TCP	
Please explain the implications of any conflict of interest and why this application should be accepted regardless		
Category		Explanation
☐	Paid positions including invited lectures and membership of advisory panels, working groups etc for which honoraria or considerations in kind were received	
☐	Shares and other commercial dealings	
☐	Financial or other sponsorship of research	
☐	Significant subsidies, whether partial or complete, for any travel, accommodation or entertainment	
☐	Gifts of any kind (greater than $50 in value)	
☐	Any other relevant activity	
Please check **both** of the statements below to acknowledge and accept the requirements of the Monash Health TCPC		
☐	I acknowledge that members of my Department/Unit are required to disclose the nature of their interests for the proposed Technology/Clinical Practice at the time of the meeting of the Monash Health TCPC. If a matter is to be decided before they are able to disclose their interest at the meeting, then they are obliged to disclose the nature of those interests as soon as possible.	
☐	I accept that if members of my Department/Unit acquire an interest that could conflict with the proposed Technology/Clinical Practice during the course of its implementation, they will disclose that by correspondence with the Chair of the Monash Health TCPC as soon as possible after the relevant facts have come to their knowledge.	

APPLICANT'S SIGNATURE					
Name		Signature		Date	

Please insert electronic signature or print <u>**this section only**</u>, sign and fax to "For TCPC" on 9594 7554

FEEDBACK
This is a pilot being implemented by the Technology/Clinical Practice Committee and the Centre for Clinical Effectiveness.<br>We would appreciate any comments regarding what works, what doesn't work and how we can improve the process.

APPENDIX 14: REQUEST FOR APPLICATION

<Date>

<Name>
<Position><Department)>
Southern Health

Dear <Name>,

Re: Request to submit an application for <new TCP> to the Southern Health TCPC

The Southern Health Technology/Clinical Practice Committee (TCPC) is aware that the <Name of Department/Unit> is undertaking/has implemented <new TCP>. This has not been approved for use at Southern Health.

The TCPC requests that an application be submitted for <new TCP> within 2 months. Upcoming Application closing dates are <Dates>. All relevant documentation and instructions for completing an application are located at http://www.mihsr.monash.org/cce/shtcp.html.

All new technologies or clinical practices introduced at Southern Health must first be approved by the TCPC before any procedures can commence. We have provided you with 2 months to complete an application. If the committee does not receive the application by **<Date>** the Chief Medical Officer, <Name> will withdraw the right to perform the procedure.

If you have any questions regarding the process for application you can contact the TCPC Administrator:
Ms Marie Garrubba
9594 7553
cce@med.monash.edu.au

Yours sincerely,

Dr Richard King
Chair, Southern Health Technology/Clinical Practice Committee

APPENDIX 15: TWO YEAR REVIEW APPLICATION FORM

REVIEW OF NEW TECHNOLOGIES OR CLINICAL PRACTICES (TCPS) FOR RECLASSIFICATION AS STANDARD PRACTICE

This process is to determine whether a recently introduced TCP can now be classified as standard practice at Monash Health or if it requires further monitoring and reporting. The review will take place two years after introduction of the TCP or earlier by request from the relevant Department Head.

For submission deadlines and meeting dates please see [Meeting Dates]

If you need assistance to complete any of the review questions please contact:

Evidence of Effectiveness
Angela Melder (Centre for Clinical Effectiveness)
9594 7575 angela.melder@monashhealth.org

Coding
Susan Peel (Health Information Services)
9594 1382 susan.peel@monashhealth.org

Current Bed Utilisation and Financial Impact
Anthony Gust (Clinical Information Management)
9594 5155 anthony.gust@monashhealth.org

Credentialing and Scope of Practice
Richard Nasra (Medical Workforce Unit)
9594 2750 richard.nasra@monashhealth.org

**Background** (to be completed by TCPC)			
Title of TCP			
Program		Department/Unit	
Brief summary of TCP			
Reason for original application	☐ Safety	☐ Effectiveness	☐ Cost Effectiveness
Brief summary of supporting evidence			

Results of Monitoring and Reporting *(to be completed by Department Head)*							
Reporting period	Patients		Procedures Performed		Successful outcomes	Deaths	Adverse Events
	Referred	Treated	Expected	Actual			
Year 1							
Year 2							
Summary of Results	(eg details of successful outcomes, other outcomes, adverse events, etc)						
Name of clinician who undertook the procedures			Number of procedures undertaken				

**Review Form** (to be completed by Department Head)						
Sites TCP is in current use	☐ Clayton	☐ Moorabbin	☐ Dandenong	☐ Casey	☐ Kingston	☐ Other
If the TCP does not apply to all sites please explain why						
What is the volume (per annum) required for maintaining skills in this TCP?						

NO	YES	
☐	☐	1. **Are there any conflicts of interest to declare regarding the ongoing use of this TCP at Monash Health?** (This includes any financial or other benefits received from groups that have a vested interest eg manufacturers, distributors, etc) Please see the Monash Health Conflict of Interest Protocol.<br>If Yes, please provide details.
☐	☐	2. **Has the TCP been used in any way other than that described in the original application?** (eg different patient group, clinical indications, sites, practitioners credentialed, etc)<br>If Yes, please outline the differences and list reasons for the variance from the application.
☐	☐	3. Has any new data been published in the research literature since the introduction of this TCP?<br>If Yes, please provide references and a brief description of outcomes
☐	☐	4. Are the rates of successful outcomes and adverse events published in the literature different to data collected for Monash Health patients?<br>If Yes, please explain differences and provide a possible reason for this?
☐	☐	5. Has the TCP performed differently to the expectation outlined in the original application in relation to operational outcomes? (eg different length of stay, use of associated services, cost of staff or consumables, unforseen outcomes, etc)<br>If Yes, please outline the differences and list reasons for the variance from the application.

☐	☐	**6. Will there be an increase in resource use and/or ongoing costs if the TCP is introduced as standard practice?** (Consider staffing and salaries, administration, specialist medical practitioners, nursing, allied health, pharmacy, theatre, intensive care, imaging, pathology, special consumables, dietary supplements, outpatient services, organisational overheads.)<br>If Yes, please compare current and future costs with details of the relevant items listed above and how the costs will be met. If there is an increase in resource use and/or ongoing costs approval is required by the relevant Executive Director.
☐	☐	7. Will any change to the current department/unit procedures list be required to incorporate the TCP if it is introduced as standard practice? (ie for credentialing and scope of practice)<br>If Yes, has the appropriate Program Director been notified? ☐ Yes ☐ No
☐	☐	8. Do any additional staff require training and credentialing if the TCP is introduced as standard practice? (Consider if credentialing and competency assurance is required by staff to ensure safe implementation)<br>If Yes, please list those persons who will be credentialed and how/where they will be trained.
☐	☐	9. The current patient information materials will require amendment if the TCP is introduced as standard practice. Please attach the amended patient information brochure
☐	☐	10. Has the TCP gone through any internal reviews such as Clinical Review Panel<br>If Yes, please note the outcome/s of the review/s.
Additional Comments		

Name of appropriate Program Director	
Name of appropriate Executive Director (Acute, Continuing Care, Mental Health, Medical Services and Quality)	
Name of appropriate Business Manager	

☐	I declare that the Program Director has received and approved a copy of this completed review	Date	
☐	I declare that the Executive Director has received and approved a copy of this completed review	Date	
☐	I declare that the appropriate Business Manager has received and approved a copy of this completed review and is satisfied that the ongoing expenses related to use of this TCP can be met within current budgets	Date	

Name				Department	
Phone		Fax		Email	

Please complete the application form and submit electronically to: **TCPC@monashhealth.org**

Prompts for Technology Clinical Practice Committee
☐ Contact Monash Health Coding. Will this TCP require a new code if it is introduced as standard practice?
<Insert Coding response here>
☐ Contact DH for data to compare patient numbers, outcomes and adverse events with data presented above to other Victorian health services.
<Insert DHS response here>
Decision
☐ Approved as standard practice at Monash Health ☐ Approved with conditions for continued monitoring *(see below)* ☐ Not Approved for continued use at Monash Health
Conditions of Approval<br>▪ To be completed by TCPC<br>Approval is granted subject to any conditions for continued monitoring outlined above.<br>Progress Reports Due:<br><TCPC to insert dates when approved with conditions for continuous monitoring>

APPENDIX 16. CHANGE OF USE APPLICATION FORM

CHANGE OF USE OF TECHNOLOGY OR CLINICAL PRACTICE (TCP)

This screening tool is designed to inform the Monash Health Technology/Clinical Practice Committee (TCPC) of any changes of use to current technology or clinical practice and to identify potential risks for the patient, clinician and organisation as a result of the change. If risks of changing the use of the current TCP are considered high, the TCPC may request further information.

For submission deadlines and meeting dates please see Meeting Dates

If you need assistance to complete any of the review questions please contact:

Evidence of Effectiveness
Angela Melder (Centre for Clinical Effectiveness)
9594 7575 angela.melder@monashhealth.org

Coding
Susan Peel (Health Information Services)
9594 1382 susan.peel@monashhealth.org

Current Bed Utilisation and Financial Impact
Anthony Gust (Clinical Information Management)
9594 5155 anthony.gust@monashhealth.org

Credentialing and Scope of Practice
Richard Nasra (Medical Workforce Unit)
9594 2750 richard.nasra@monashhealth.org

<table>
<tr><td colspan="7">Application Form</td></tr>
<tr><td>Title of TCP</td><td colspan="6"></td></tr>
<tr><td>Program</td><td colspan="2"></td><td colspan="2">Department/Unit</td><td colspan="2"></td></tr>
<tr><td>Brief summary of change of use</td><td colspan="6"></td></tr>
<tr><td>☐ New indication for current patient group</td><td>☐ New patient group</td><td>☐ Modification of equipment/technique</td><td colspan="2">☐ New operators/ practitioners</td><td colspan="2">☐ Other</td></tr>
<tr><td>Reason for change of use</td><td>☐ Safety</td><td>☐ Effectiveness</td><td colspan="4">☐ Cost Effectiveness</td></tr>
<tr><td colspan="2">Brief summary of supporting evidence</td><td colspan="5"></td></tr>
<tr><td>Sites TCP is in current use</td><td>☐ Clayton</td><td>☐ Moorabbin</td><td>☐ Dandenong</td><td>☐ Casey</td><td>☐ Kingston</td><td>☐ Other</td></tr>
<tr><td>Sites where C of U</td><td>☐ Clayton</td><td>☐ Moorabbin</td><td>☐ Dandenong</td><td>☐ Casey</td><td>☐ Kingston</td><td>☐ Other</td></tr>
<tr><td colspan="7">If change does not apply to all sites please explain why</td></tr>
</table>

NO	YES	
☐	☐	1. Are there any conflicts of interest to declare that relate to this change of use? (This includes any benefits received from groups that have a vested interest in the change of use proposed eg paid positions including invited lectures and membership of advisory panels; working parties or other groups for which honoraria or considerations in kind were received; shares and other commercial dealings; financial or other sponsorship of research; significant subsidies, whether partial or complete, for any travel, accommodation or entertainment; gifts of any kind greater than $100 in value.) Please see the Monash Health Conflict of Interest Protocol.<br>If Yes, please provide details.
☐	☐	2. Is there any reason to suspect increased harm to patients with the change of use? (Consider side effects, contraindications and adverse events that might be different with the change of use)<br>If Yes, please list reasons for suspected increase in harm to patients.
☐	☐	3. Are there any establishment costs related to the change of use?<br>If Yes, please provide details of costs and how cost will be met. Costs to be approved by the relevant Executive Director.
☐	☐	4. Will there be an increase in resource use and/or ongoing costs with the change of use? (Consider staffing and salaries, administration, specialist medical practitioners, nursing, allied health, pharmacy, theatre, intensive care, imaging, pathology, special consumables, dietary supplements, outpatient services, organisational overheads.)<br>If Yes, please compare current and future costs with details of the relevant items listed above and how the costs will be met. If there is an increase in resource use and/or ongoing costs to approval is required by the relevant Executive Director.
☐	☐	5. Will the change of use impact on other clinical disciplines or services? (Consider items in question 4)<br>If Yes, please provide details of which clinical disciplines will be affected and how.

☐	☐	6. Will the change of use require a new code?<br>If Yes, please provide the new code.
☐	☐	7. Will any change to the current department/unit procedures list be required to incorporate the change of use? (ie for credentialing and scope of practice)<br>If Yes, please notify the appropriate Program Director
☐	☐	8. Do any staff require additional training and credentialing for the change of use? (Consider if credentialing and competency assurance is required by staff to ensure safe implementation)<br>If Yes, please list those persons who will be credentialed and how/where they will be trained.
☐	☐	9. Will staff be required to change their practice for the change of use? (Consider if staff will be required to change their practice, are there anticipated barriers associated with this, will staff require further education, will the change of use require a dissemination and implementation program?)<br>If Yes, please provide details of your plan for implementation across all relevant sites.
☐	☐	10. Will any relevant, previously eligible patients not have access to the change of use? (Consider whether the patient group currently accessing the TCP will still have access to it when the change is introduced)<br>If Yes, please provide details of which patients will not have access and why.
☐	☐	11. Are there any ethical issues to be considered with the change of use?<br>If Yes, please describe ethical issues to be considered.
☐	☐	12. Are there any legislative or regulatory requirements related to the change of use? (Consider TGA approval, Australian Standards, Professional body requirements, prescribing legislations, etc.)<br>If Yes, please describe legislative and regulatory requirement related to change of use.
☐	☐	13. Does this technology/clinical practice have a radiation source?<br>If Yes, please confirm that it complies with the Monash Health licensing agreement
☐	☐	14. Do the current patient information materials require amendment for the change of use? (Consider if specific risks arising from the proposed change of use have been included and patients explicitly informed.)<br>If Yes, please verify that amendments have been made.
☐	☐	15. Are there any additional risks to patients, staff or the organisation due to this change of use? (Consider injury, damage to reputation, financial and legal implications.)<br>If Yes, please describe potential risks to patients, staff or the organisation.

Additional Comments	
Name of Head of Department/Unit	
Name of appropriate Program Director	

☐	I declare that the Head of Department has received and approved a copy of this completed application	Date	
☐	I declare that the appropriate Program Director has approved any expenses or additional use of Monash Health resources that arise from the change of use of this TCP (as outlined in this application)	Date	

Applicant Name				Position	
Phone		Fax		Email	

Please complete the application form and submit electronically to: **TCPC@monashhealth.org**

Decision
☐ Approved ☐ Approved with conditions *(see below)* ☐ Not Approved
Conditions of Approval<br>▪ To be completed by TCPC<br>Approval is granted subject to any conditions outlined above. Implementation of the change should not commence until all conditions are met.<br>Please forward confirmation that the conditions have been met to TCPC@monashhealth.org by <insert date>.

APPENDIX 17. HREC TCPC APPLICATION FORM

HREC TCPC APPLICATION

HREC applications involving a procedure or clinical practice new to Monash Health

Please note that this form is in draft. We would appreciate any feedback you may have on it.

This tool is designed to inform the Human Research and Ethics Committee and the Technology/Clinical Practice Committee (TCPC) of any financial, operational and/or credentialing requirements arising from the use of a new clinical procedure or practice in the context of a research project and to identify potential risks for patients, clinicians and the organisation as a result. This information will be considered as part of your research application and additional conditions may be placed on the project if required after this analysis.

If you need assistance to complete any of the screening questions please contact:

Credentialling and Scope of Practice
Richard Nasra
Medical Workforce Unit
9594 7678
richard.nasra@monashhealth.org

Current Bed Utilisation and Financial Impact
Anthony Gust
Clinical Information Management
9594 5155
anthony.gust@monashhealth.org

Coding
Susan Peel
Health Information Services
9594 1382
susan.peel@monashhealth.org

How to complete this form

- Please answer every question
- To complete written answers, insert cursor in grey box and commence typing
- To select answer from available options, double click on the appropriate box and select 'checked'

Submissions

- All applications should be submitted electronically to TCPC@monashhealth.org
- For submission deadlines please see: Meeting Dates

Please note that approval of a procedure/practice as part of a research project does not indicate support for introduction of the procedure/practice outside a research context. Approval is contingent on a current HREC approval certificate for the duration of the research project. At the conclusion of the research project a new procedure/practice cannot be continued at Monash Health without a separate application made for introduction of a new TCP to the TCPC

Introductory information			
**1.** Lead Monash Health Clinician			
**Name:**	**Title:**	**Position:**	
**Phone:**	**Email:**		
**2.** Title of Research Project<br>2A. HREC Reference Number (if allocated):			
**3.** Brief summary of new procedure/clinical practice associated with the research application and the participant group:			
**4.** What is the clinical indication/disease/condition?			
**5.** Are there any potential conflicts of interest to declare that relate to this change of procedure or clinical practice? Please see the Monash Health Conflict of Interest Protocol.			

NO	YES	In relation to this procedure/practice, has the unit or will the unit obtain...?	If Yes, please provide details
☐	☐	Paid positions in the unit/department	
☐	☐	Invited attendance at lectures/conferences for which honoraria or considerations in kind were received	
☐	☐	Membership of advisory panels	
☐	☐	Working parties or other groups for which honoraria or considerations in kind were received	
☐	☐	Shares and other commercial dealings	
☐	☐	Financial or other sponsorship of research	
☐	☐	Significant subsidies, whether partial or complete, for any	

		travel, accommodation or entertainment	
☐	☐	Gifts of any kind greater than $100 in value	

Assessment of impact on participants

6. What are the anticipated risks to participants with the new procedure / clinical practice?

Consider side effects, adverse events etc

7. What approved procedure/ practice is currently used for this clinical indication/disease/condition?

8. Describe how the proposed procedure/practice differs from current practice.

9. What arrangements have been made regarding any readmission of participants who undergo the new procedure/ practice?

Consider who these participants will be admitted under, how risk associated with the new procedure/practice will be assessed and documented

10. Will the introduction of the new procedure/practice effect demand management? ☐ Yes ☐ No
If yes, please describe how and how this will be managed.

11. Are there any additional considerations/ issues relating to participants due to this change of clinical procedure/practice? ☐ Yes ☐ No

If Yes, please describe together with planned mitigation strategies; increased operating theatre sessions

Assessment of financial and operational implications

12. Are there any establishment costs related to the new procedure / clinical practice? ☐ Yes ☐ No

If Yes, please provide details of costs and how cost will be met. Any costs not covered by the Funding Organisation (eg NHMRC) or Commercial Sponsor

MUST be approved by the relevant Executive Director.

13. Will there be an increase in resource use and/or ongoing costs with the new procedure / clinical practice?

		Is there any increased utilisation of...? If Yes, please describe and compare current and future costs in right hand column	Please indicate where the increased utilisation will occur			Describe element and how costs will be funded
			Pre-admission	Inpatient care	Pre-admission	
NO	YES	Any change that has cost or resource implications must be approved by an Executive Director.				
☐	☐	Specialist Medical Practitioner				
☐	☐	Allied Health				
☐	☐	Nursing				
☐	☐	Pharmacy				
☐	☐	Theatre (sessions, other resources)				
☐	☐	Intensive Care				
☐	☐	Imaging				
☐	☐	Pathology				
☐	☐	Consumables not considered elsewhere				
☐	☐	Dietary supplements				
☐	☐	Out services / sessions				
☐	☐	Organisational overheads				
☐	☐	Other; please specify:				
☐	☐	Specialist Medical Practitioner				

14. Will the average length of stay for this clinical indication/disease/condition increase? ☐ Yes ☐ No

How is this being funded?

15. Will the number of participants being treated for this condition increase because we have a new procedure / practice? ☐ Yes ☐ No

How is this being funded?

Impact on broader organisation

16. Will the procedure / practice impact on other clinical disciplines or services? *(Consider items in question 4). If Yes, please describe:*

Which clinical disciplines will be affected? How?	
What consultation has occurred with these disciplines and any agreements reached about patient care	
How will costs attributed to the procedure but occurring within another unit be paid for?	

17. Is a prosthesis/device/drug being used? ☐ No ☐ Yes; please describe

Is the prosthesis/device/drug TGA approved? ☐ No ☐ Yes

18. Are there any legislative or regulatory requirements related to the change of clinical procedure/practice? If Yes, please describe. Consider Australian Standards, Professional body requirements, prescribing legislations, etc.

19. Does this procedure / practice have a radiation source? If Yes, please confirm that it complies with the Monash Health licensing agreement

☐ No

☐ Yes; complies with Monash Health licensing agreement? ☐ Yes ☐ No. Confirmed by:

20. Are there any additional risks to staff or the organisation due to this procedure/practice? ☐ Yes ☐ No (Consider injury, damage to reputation, financial and legal implications.).

If yes, please describe potential risks to participants, staff or the organisation.

21. How do the risks compare with the current Gold Standard/Best Practice?

22. Will there be any unanticipated consequences? (eg change in the position of the patient on the elective surgery waiting list due to being involved in this study) ☐ Yes ☐ No

If yes How will these be managed?

Credentialing and scope of practice

23. Do any staff require additional training and credentialing for procedure / practice to ensure safe implementation? Your Program Medical Director must approve any changes to unit staff credentials)

NO	**YES**	**Is credentialing and / or competency assessment required for...?**	If Yes, please describe: Who will be credentialed?	What training is required?	How will credentialing occur?	What body has established and/or will recognise the credentials?	What credentials will be added the unit Part B?
☐	☐	Medical Staff in the unit					
☐	☐	Medical staff in *other* units					
☐	☐	Nursing Staff in the unit					
☐	☐	Nursing staff in *other* units					
☐	☐	Technical Staff in the unit					
☐	☐	Technical staff in *other* units					
☐	☐	Medical Staff in the unit					

**24.** For each group that does require additional training or credentialing, has this additional training/competency assessment been undertaken?:<br>☐ YES; Date completed: ...../...../.....; Sign off of credentials completed by: Name: Role:<br>☐ NO; If NO, how and when will this occur?
**25.** Any additional Comments regarding credentialing or training for the procedure / practice?

APPLICANT'S SIGNATURE		
**Name:**	**Signature:**	**Date:**
ENDORSEMENT BY HEAD OF DEPARTMENT/UNIT		
**Name:**	**Signature:**	**Date:**
ENDORSEMENT BY PROGRAM DIRECTOR		
**Name:**	**Signature:**	**Date:**
ENDORSEMENT BY DIRECTOR OF NURSING		
**Name:**	**Signature:**	**Date:**
ENDORSEMENT BY EXECUTIVE DIRECTOR		
Name:	Signature:	Date:
ENDORSEMENT BY PROGRAM DIRECTOR OF ANY OTHER AFFECTED UNITS DESCRIBED ABOVE		
Name:	Signature:	Date:

Please complete the application form and submit electronically to: **TCPC@monashhealth.org**

TCPC use only	
Actions:	
☐ Referred to TCPC Executive	☐ Requires TCPC representation at HREC
☐ Referred to whole of TCPC Committee	☐ Requires joint sitting of HREC/TCPC
☐ Other action: please describe:	

Decision		
☐ Approved	☐ Approved with conditions *(see below)*	☐ Not Approved
**Conditions of Approval:** Approval is granted subject to any conditions outlined. Implementation of the change should not commence until all conditions are met.		
Any other comments:		

**Name:**	**Date:**
**TCPC role:**	Signature:

APPENDIX 18. FEEDBACK FORM

MONASH HEALTH EVALUATION
Monash Health is piloting this application process. In order to make it as user-friendly as possible we welcome your input. Please provide feedback on any/all of the items below.
**Content and wording**<br>The questions in this application are taken from the DH 'Guidance for Victorian Public Health Services to Establish Technology/Clinical Practice Committees'. These are the minimum recommended by DH for technology/clinical practice applications. Monash Health has added questions related to conflict of interest. ***Monash Health has no control over the DH recommended questions.*** However, if you have feedback regarding the content or wording of the application we will communicate your views to DH.
What worked well? Why?
What didn't work well? Why?
Should anything else be included?
Should anything be excluded?
How could DHS improve the content or wording?
**Format**<br>The questions have been re-formatted to integrate instructions, requirements and information contained in appendices into the body of the document to assist applicants. Please provide relevant feedback regarding the document format, ease of use, etc.
What worked well? Why?
What didn't work well? Why?
How could we improve the format?
**Resources**<br>Was the 'Searching for the Evidence' resource guide helpful? Please let us know what you think.
What worked well? Why?
What didn't work well? Why?
How could we improve it?
Should we develop similar resources for other aspects of the application? If so, what?
Should we develop similar resources to help you in any other aspects of your work? If so, what?
**Assistance**<br>Was the assistance provided by HIS, CIM, CCE and Finance helpful? Please let us know what you think.
What worked well? Why?
What didn't work well? Why?
How could we improve it?
Should we develop similar resources to help you in any other aspects of your work? If so, what?
Other comments

APPENDIX 19. NOTIFICATION OF CONDITIONS

<Date>

<Name>
<Head of Department/Unit Manager>
<Department/Unit>

Dear <Name>,

Re: <TCPC Application Number and Title>

I write to advise that the application for **<new TCP>** at Southern Health was approved by the Technology/Clinical Practice Committee (TCPC) on **<Date>**.

Standard approvals for new TCPs are based on restricted recommendations requiring audit of patient outcomes. The decision to recommend **<new TCP>** is based on the following conditions:

General Conditions

a. The Head of Department/Unit is required to notify the Secretariat of TCPC in the event of:

- Any change in protocol and the reason for that change together with an indication of ethical implications
- Adverse effects of the TCP and steps to deal with them
- Any unforeseen events

b. Adverse Events

- If an adverse event occurs the Head of Department/Unit must immediately notify the TGA in addition to the TCPC.

c. Compliance with Quality Assurance *(must be completed prior to commencement of the TCP at Southern Health*)

- Applicants are required to complete either the Quality Assurance supplement letter or a new Quality Assurance application (whichever is applicable) and forward to Southern Health HREC

d. Data Collection

- Data to be collected in all patients receiving the new TCP and reports provided to TCPC. The TCPC will provide details of data required by DHS.

e. Reporting

- Reporting required at six monthly intervals (January – June and July – December) for a two year period.
- Reports to be forwarded to TCPC Secretariat. TCPC to forward reports to DHS.

f. Review

- At the conclusion of the two year period the original application will be reviewed by the TCPC to determine if it should be considered standard practice.

Special Conditions

Please note that some conditions must be met prior to commencement of the procedure **<Date>**. Completion of these should be notified to the TCPC Executive Officer via email Richard.nasra@southernhealth.org.au.

Progress Reporting Dates

Due date of first progress report **<Time period>**	**<End of Feb or Aug>**
Due date of second progress report **<Time period>**	**<End of Feb or Aug>**
Due date of third progress report **<Time period>**	**<End of Feb or Aug>**
Due date of fourth progress report **<Time period>**	**<End of Feb or Aug>**

Please see attached Decision Summary for further information.

Yours sincerely,

Dr Cate Kelly

Chair, Southern Health Technology/Clinical Practice Committee

APPENDIX 20. LETTER EXPLAINING COMPLIANCE WITH QUALITY ASSURANCE

<Date>

<Name>
<Position><Department>
Southern Health

Dear <Name>,

Re: Quality Assurance application for clinical audit following implementation of <new TCP>

As you know, the Department of Human Services (DHS) requires regular reporting of patient outcomes following introduction of new technologies and clinical practices. This clinical audit requires approval by the Southern Health Human Research Ethics Committee (HREC) as a Quality Assurance activity.

The Technology/Clinical Practice Committee (TCPC) has arranged generic approval for the items in the DHS Progress Report; however the HREC requires notification of individual clinical audits. A letter proforma has been prepared to facilitate this process for you.

Please read the attached Quality Assurance application. If you are happy that your audit complies with the description outlined please forward the attached letter to HREC. If you plan to collect any additional information (ie in addition to the items on the DHS Progress Report), details of the supplementary audit must also be provided.

Publication of any patient outcome data related to **<new TCP>** is not covered by the generic HREC application for Quality Assurance submitted by the TCPC. You must inform the HREC of your individual audit by forwarding the attached letter or submitting a separate Quality Assurance application.

Please contact me if you would like any further information about this process.

Yours sincerely,

Marie Garrubba
Administrator, Southern Health Technology/Clinical Practice Committee
Phone: 9594 7553
Email: marie.garrubba@med.monash.edu.au

APPENDIX 21. TCP QUALITY ASSURANCE APPLICATION

Project title: Southern Health Technology/Clinical Practice Committee monitoring and reporting requirements for introduction of new technologies and clinical practices

Principal Investigator: Dr Claire Harris
Director
Centre For Clinical Effectiveness
Southern Health

Phone number: 9594 7576

Email address: Claire.Harris@med.monash.edu.au

Postal Address: Locked Bag 29, Clayton, 3168

In accordance with the National Health and Medical Research Council publication titled, '*When does quality assurance in health care require independent ethical review*?' (20 February 2003) this document is intended for review by the Executive Officer of the HREC and Medical Administrator to determine whether this project requires formal review by a Human Research Ethics (HREC) Committee.

Summary of project

The primary role of the Southern Health Technology/Clinical Practice Committee (TCPC) is to oversee and support clinicians in the safe and appropriate introduction of a technology or clinical practice that has not previously been undertaken within the organisation. Once a new technology or clinical practice is approved, the secondary role of the TCPC is to monitor the patient outcomes of that procedure for a two year period.

Based on DHS guidance, the TCPC has developed a progress reporting template and patient outcome spreadsheet for clinicians implementing new TCPs to record the required information. Progress reports are then forwarded to DHS every six months for the reporting periods January – June and July – December.

To fulfil monitoring and reporting requirements for DHS, the TCPC require approval for collation of patient outcome data for the progress report and spreadsheet. This information will contribute to the review of the new technology or clinical practice, by the TCPC, to determine whether it can be classified as current routine practice and thus no longer require monitoring.

NHMRC Questions to be considered:

Consent

1) Is the consent from participants *inadequate* or is the activity *inconsistent* with National Privacy Principle 2.1(a)?

Participants may include patients, carers, health care providers and the institution involved.
The participants in the project will include managers, clinicians and the organisation (Southern Health). Participants are informed of the audit process upon application. Receipt of appropriate reporting and audit information will be considered implied consent.

All activities will be consistent with National Privacy Principle 2.1(a).

Risks and Burdens

2) Does the proposed quality assurance activity pose any risks for patients beyond those of their routine care?

Risks include not only physical risks, but also psychological, spiritual and social harm or distress, e.g. stigmatisation or discrimination.

The proposal poses no physical risks or any potential for psychological harm to patients beyond those of their routine care. This quality assurance project will not require any patient involvement.

3) Does the proposed quality assurance activity impose a burden on patients beyond that experienced in their routine care?

Burdens may include intrusiveness, discomfort, inconvenience or embarrassment, e.g. persistent phone calls, additional hospital visits or lengthy questionnaires.

The proposal imposes no burdens on patients beyond that experienced in their routine care. The quality assurance project will not involve the participation of patients in surveys, interviews or similar data collection activities.

Privacy and Confidentiality

4) Is the proposed quality assurance activity to be conducted by a person who does not normally have access to the patient's records for clinical care or a directly related secondary purpose?

Review of medical records unavoidably risks the privacy of individuals. However, authorised audit of records is an extremely valuable quality assurance activity. Provided the individual reviewing the records is bound by legislation or a professional code of ethics, this question can be answered in the negative.

There may be some auditing of patient data by Southern Health clinicians who are bound by the code of ethics. Data submitted to the TCPC is in a de-identified form. The TCPC will not collect any information that identifies individual patients.

5) Does proposed quality assurance activity risk breaching the confidentiality of any individual's personal information, beyond that experienced in the provision of routine care?

A quality assurance activity that requires a letter, fax or email to a patient that includes sensitive health information could lead to a breach of confidentiality if the communication is read by someone other than the proposed recipient.

The proposal does not risk breaching the confidentiality of any individual's personal information, beyond that experienced in the provision of routine care.

Overlap with Research

6) Does the proposed quality assurance activity involve any clinically significant alteration to the routine clinical care provided to the patients?

Application and evaluation of a new technology not previously used in the hospital may need further consideration.

The proposed quality assurance project will not involve any alteration to the routine clinical care provided to the patients.

7) Does the proposal involve randomisation or the use of a control group or a placebo?

Proposals involving comparison with published or prior treatment results with other groups are acceptable.

The proposal does not involve randomisation or the use of a control group or a placebo.

8) Does the proposed quality assurance activity seek to gather information about the patient beyond that collected in the routine clinical care?

Information may include observations, blood samples, additional investigations etc. Genetic studies in particular may provide information about families and relatives as well as the individual patient, and must be referred to an HREC.

The proposal does not seek to gather information beyond that collected in the routine clinical care.

Broader Implications

9) Does the proposed quality assurance activity potentially infringe the rights, privacy or professional reputation of carers, health care providers or institutions?

These issues should be considered by management and may have legal implications. Consideration may need to be given to the relevant State or Territory legislation with respect to legal privilege for a quality assurance body.

The proposal will not infringe the rights, privacy or professional reputation of carers, health care providers or institutions. Progress reports and patient outcome data related to the newly approved technology or clinical practice will be collated by Southern Health clinicians. This will be submitted to the TCPC in de-identified format. The TCPC will not have access to any information that will identify individual patients. Any results that are published or presented will be in summary form only and any critical issues will be presented in a general manner not linked to specific patients or clinicians. Questions required by the clinical audit of information are included in Appendix A: Progress Report.

*** Will the proposal generate data that are likely to lead to publication in peer-reviewed or professional journals?**

Many journals require review and acceptance as ethical by an HREC before accepting articles for publication. If it is intended that the results of the quality assurance study will be published, it is wise to obtain prospective HREC approval.

The TCPC is unlikely to publish clinical audit information related to individual technologies and clinical practices. Clinicians may wish to publish data for specific TCPs and will submit Quality Assurance applications for this purpose.

The information collected by the TCPC will be aggregated into a report and submitted to DHS at six monthly intervals.

APPENDIX 22. TCP QUALITY ASSURANCE SUPPLEMENT LETTER

<Date>

<Name>
Director
Research Services
Research Directorate
Southern Health

Dear <Name>,

Re: Supplement to Quality Assurance application #09195Q: Clinical audit following introduction of <new TCP>

<New TCP> has been approved by the Southern Health Technology/Clinical Practice Committee (TCPC). As part of the reporting requirements to DHS and the Southern Health Executive Management Team, a clinical audit will be undertaken for two years following implementation.

I have read the previously approved TCPC Quality Assurance application **<Number>** and can confirm that the data to be audited is consistent with the generic information provided. No additional information will be collected.

I would like to submit this letter as a supplement to the generic document provided by TCPC.

Please let me know if you require any additional information.

Yours sincerely

<Applicant Name>
<Applicant Title>
<Phone: >
<Email: >

APPENDIX 23. DATA COLLECTION SPREADSHEET

INSTRUCTION FOR RECORDING PATIENT OUTCOMES

This excel file was developed to make the process of capturing and reporting information as easy as possible for applicants approved to introduce a new technology or clinical practice to Monash Health.

It was drafted based on a minimum generic data set outlined by the Department of Human Services (DHS).

You will find included two spreadsheets for collection of information for:

1. Treated Patients
2. Referred but Untreated Patients

Please be aware that DHS requires all newly introduced technologies/clinical practices to report on referred patients who did not receive treatment.

We would ask that you complete the attached spreadsheets for all patients treated and untreated and forward along with your progress report to the TCPC Executive Officer electronically (TCPC@monashhealth.org) at 6 monthly intervals (**August** for the January to June reporting Period and **February** for the July to December reporting period).

If you currently have an outcome auditing system in place that you prefer to use please feel free to forward us the required information in that format.

As we are currently piloting this process we would be happy to receive any feedback you may have (TCPC@monashhealth.org)

Untreated Patients

Title of TCP				
Reporting Period				
UNTREATED REFERRED PATIENTS REPORTING	Patient 1	Patient 2	Insert as needed	Total
UR				
Indication (customise as appropriate)				
Reasons for patients not being treated				
Inappropriate referral (Yes=1 No=0)				
Declined treatment (Yes=1 No=0)				
Treated elsewhere (Yes=1 No=0)				
Too sick for treatment - related to condition for referral (Yes=1 No=0)				
Too sick for treatment - related to other factors (Yes=1 No=0)				
Death - related to condition for referral (Yes=1 No=0)				
Death - related to other factors (Yes=1 No=0)				
Still awaiting treatment (Yes=1 No=0)				
Other (please specify)				

Treated Patients

Title of TCP				
Reporting Period				
TREATED PATIENTS OUTCOME REPORTING	Patient 1	Patient 2	Insert as needed	Total
UR				
Clinician/s who performed procedure				
Number of procedures performed				
Date of 1st procedure				
Date of 2nd procedure				
(insert as appropriate)				
Indication (customise as appropriate)				
Site (Clayton, Casey, Dandenong, Moorabbin, Other)				
Result of Procedure				
Uncomplicated treatment - Failure post-treatment (Yes=1 No=0)				
Uncomplicated treatment - Successful completion (Yes=1 No=0)				
Complicated treatment - Failure post-treatment (Yes=1 No=0)				
Complicated treatment - Successful completion (Yes=1 No=0)				
Death post treatment (Yes=1 No=0)				
Adverse outcomes				
Nosocomial infection during the reporting period? (Y=1 No=0)				
If Yes, provide details				
Other adverse outcomes during the reporting period? (Yes=1 No=0)				
If Yes, provide details				
Unplanned readmission to intensive care (Yes=1 No=0)				
If Yes, provide details				
Unplanned readmission post discharge (Yes=1 No=0)				
If Yes, provide details				
If Yes, to any of the above adverse events was the TGA informed? (Yes=1 No=0)				
If Yes, to any of the above adverse events was the TCPC informed? (Yes=1 No=0)				
Other outcome measures specific to procedure				
(please customise as appropriate)				

APPENDIX 24. PROGRESS REPORT TEMPLATE

PROGRESS REPORT

Reporting requirements

Progress Reports for new Technologies/Clinical Practices (TCPs) are to be completed by the relevant Head of Department/Unit and forwarded to the Monash Health Technology/Clinical Practice Committee (TCPC) at 6 monthly intervals (**August** for the January to June reporting period and **February** for the July to December reporting period).

The Progress Report will be reviewed by the Chair of the TCPC and submitted to the Monash Health Executive Management Team.

The information below is required by the Victorian Department of Health (DH). Reports on all new technologies and clinical practices implemented at Monash Health will be forwarded to DH by the TCPC in March and September.

Completing this form

- To enter information, click once on the grey rectangle and begin typing.
- To fill in check boxes, click once on the appropriate square.

Enquiries and submission of the report can be directed to the Executive Officer on 9594 7575 or TCPC@monashhealth.org

A. OVERVIEW			
Title of TCP			
Application #		Reporting Period	
**1.** Has the TCP been introduced?			
☐ YES - commencement date		☐ NO - reason	
**2.** Is it continuing?			
☐ YES		☐ NO - reason	
**3.** Number of procedures performed in the current reporting period.			
**4.** Total number of patients that have had the procedure.			
**5.** Number of patients referred but still awaiting procedure.			
**6.** Number of deaths if any during the waiting period.			
Please give details:			
**7.** Patient classification (eg. inpatient/outpatient/other).			
B. OUTCOMES			
**8.** If this is a Victorian Policy Advisory Committee on Technology (VPACT) funded technology or clinical practice, have the quarterly reports to VPACT been provided?			☐ YES ☐ NO ☐ N/A
**9.** If this is a new technology not funded by VPACT has six monthly audit data been provided to the Monash Health TCPC? (You may use the outcome spread sheet provided or your own audit template)			☐ YES ☐ NO ☐ N/A
**10.** Outcomes for patients treated during the reporting period (please provide numbers).			
Uncomplicated treatment – successful completion			
Uncomplicated treatment – failure post-treatment			
Complicated treatment – successful completion			
Complicated treatment – failure post-treatment			
Death post-treatment			
**11.** Please provide details of complications/failed treatment outcomes.<br>Complications include known risks of the TCP.			

C. ADVERSE OUTCOMES	
**12.** Have there been any adverse outcomes or significant problems in the current reporting period?<br>Adverse events are unexpected outcomes. In particular any Incident Severity Rating Category 1 (Severe) or Category 2 (Major) events should be reported here.	
Nosocomial infection	
Unplanned readmission to intensive care	
Unplanned readmission post discharge	
Other adverse outcome	
**13.** Please provide details of reasons for adverse outcomes.	
**14.** Have the patients or carers of patients raised any issues?	

D. ONGOING USE		
**15.** Has there been a change in the application/use of the TCP?<br>If YES, please provide details	☐ YES	☐ NO
**16.** What is anticipated for future application/use of the TCP? For example, do you anticipate a change of indication, rollout to additional Monash Health sites etc.		
**17.** Estimated number of procedures to be performed in the next reporting period.		
**18.** How has this technology made a difference? Include patient well-being and quality of life, hospital stay, satisfaction of the primary carer etc (append up to 600 words), quality of life, employability, cost-effectiveness etc.		
**19.** Have any publications, conference presentations or other presentations occurred during this period?<br>☐ YES ☐ NO<br>If YES, please provide details (append additional pages if necessary)		
**20.** Has there been any progress toward organising a public launch of the technology?<br>☐ YES ☐ NO<br>If YES, please provide details		

Completed by		Date	
Dept/Unit Head		Date	

FEEDBACK
We would appreciate any comments regarding this form and how we can improve this reporting process.

APPENDIX 25. CORRESPONDENCE REGARDING REPORTING COMPLIANCE

Dear <Name>,

Re: Progress Report template and patient outcome audit spreadsheet

I would like to draw your attention to the reporting requirements that are conditions of approval for your application **<Application Number and title>.**

DHS require 6 monthly reporting on a minimum generic data set plus information regarding specific outcomes related to your application.

We have developed templates to make the process of capturing and reporting information as easy as possible for you. Please find attached a report proforma and outcome spreadsheet.

We have drafted the spreadsheet based on information in your application but realise that this may not be technically correct. Please feel free to adapt to reflect the correct outcome measures. If you currently have an outcome auditing system in place you are free to forward us the required information in that format.

Your first progress report will be due on <Day, Date, Year>. (Allow 1 month from date approval letter was sent)

Please forward completed copies of the outcome spreadsheet or alternatively your own audit tool and the report proforma to cce@med.monash.edu.au by the above date.

Yours sincerely,

Ms Marie Garrubba

TCPC Administrator 9594 7553 cce@med.monash.edu.au

Dear <Name>,

Re: Reporting requirements for <Application Number & Name>

This is a courteous reminder to inform you that the progress report is due for submission to the TCPC by **<Date>.**

The **Progress Report Template** and **Patient Outcomes Data Spreadsheet** should be completed for all patients referred and treated up to the end of **<appropriate period>**.

Could you please forward the required information to me by the due date to ensure that the TCPC is able to fulfil its reporting requirements to the Department of Health.

Please feel free to contact me if you have any questions.

Kind Regards

Ms Marie Garrubba

TCPC Administrator 9594 7553 cce@med.monash.edu.au

Dear <Name>,

Re: Progress Reporting for <Application Number & Name>

The Southern Health Technology/Clinical Practice Committee (TCPC) is yet to receive progress reporting and patient outcome data for **<Application Number & Name>** from **<Date of reporting period>.**

As you will be aware your application for **<Application Number & Name>** was approved on the conditions that progress reporting be completed and forwarded to the TCPC six monthly for a period of two years.

Can I please request that you forward the **<Date of reporting period>** Progress Report and patient outcome data to Marie Garrubba (marie.garrubba@southernhealth.org.au) by **<Date>.**

Yours sincerely,

A/Prof Richard King

Chair, Southern Health Technology/Clinical Practice Committee

Program Director, Medicine Program

APPENDIX 26: PATIENT INFORMATION BROCHURE

MonashHealth

Patient ID Label

This patient information is for a new **<technology, clinical practice, device>** that has been approved for introduction at Monash Health.

Your **<doctor/surgeon/other>** has recommended **<procedure>**. However, it is your decision whether to go ahead with the procedure.

This document gives you information about the reasons for the procedure, and about the benefits and risks of the procedure, so that you can make an informed decision.

What is < procedure>?

< insert text>

What causes <indication >?

< *If applicable* insert text>

What are the benefits of <procedure>?

- Describe the short and long term benefits to the patient

< insert text>

Are there any alternatives to <procedure>?

- Describe any appropriate alternative procedures or treatments that may be of benefit to the patient
- Describe standard treatment and its effectiveness
- Indicate how this new technology/clinical procedure differs from standard treatment
- Include disadvantages from withholding standard treatment
- If there is no alternative treatment clearly state this

< insert text>

What will happen if I decide not to have the procedure?

< insert text>

What does the procedure involve?

- State the nature of the procedure
- Indicate the length of time needed for the procedure
- In the case of medical devices/technologies, information should be provided about the mechanisms in place to track patients for the lifetime of the device, to detect any relevant adverse events and enable remedial action if a significant defect is detected
- It is advisable to include a statement noting that continual review and monitoring will take place, regarding the efficiency and safety of the procedure, and this will enable early detection of any problems patients may suffer

< insert text>

What should I do about my medication?

It is important to tell clinical staff about any treatments or medications you may be taking, including non-prescription medications, vitamins or herbal remedies.

What can I do to help make the procedure a success?

< *If applicable* insert text>

What complications can happen?

- Eg complications of anaesthesia, complications of any procedure, complications of this procedure

< insert text>

How soon will I recover?

< insert text>

Summary

< insert text>

Acknowledgements

< *If applicable* insert text>

Further Information

Patients experiencing complications as a result of their recent procedure should contact the Monash Health switch board and ask to be put through to the relevant registrar on call.

MMC Clayton	MMC Moorabbin	Dandenong	Casey	Kingston
03 9594 6666	03 9928 8111	03 9554 1000	03 8768 1200	03 9265 1000

All other queries should be directed to your treating doctor.

Write questions or notes here

<Month, Year>

APPENDIX 27: AGENDA TEMPLATE

TECHNOLOGY/CLINICAL PRACTICE COMMITTEE

Agenda

Date and Time:	
Location:	
Members:	
Invitees:	

(KEY: Attachments indicated by (*)			
	Item		Presenter (initials)
1.	Apologies:		
2.	Confirmation of previous minutes <meeting date>		
3.	Declaration of conflicts of interest		
4.	Business arising from previous minutes for discussion		
5.	New Business		
	5.1	New applications	
		5.1.1 <Application Title>	
	5.2	Change of use application	
		5.1.2 <Application Title>	
	5.3	Applications for review for standard practice	
		5.1.3 <Application Title>	
	5.4	Adverse events	
		5.1.4	
	5.5	HREC application entailing a TCP new to Monash Health	
		5.1.5 <Application Title>	
	5.6	Extraordinary applications	
		5.1.6 <Application Title>	
7.	Credentialing follow up		
	7.1		
9.	Other Business		
	9.1	Any other business?	
10.	Date of next meeting <Date, Time, Location>		

Business arising from previous minutes - tabled items

Action Item	Action in progress for discussion by the Committee	Person Responsible	Timeline
1.			
	Actions in Progress awaiting response from second party		
1.			
	Actions Completed		
1.			

APPENDIX 28: MINUTES TEMPLATE

TECHNOLOGY/CLINICAL PRACTICE COMMITTEE

Minutes

Date and Time	
Location	
Present: Committee members	
Present: Invitees	

Agenda Item	Discussion/Decision	Action	By Whom (Date)
**1.** Apologies			
**2.** Confirmation of previous minutes			
**3.** Declaration of conflicts of interest			
**4.** Business arising from previous minutes – for discussion			
**5.** New Business			
5.1 New Expression of Interest Applications			
5.2 Change of Use Applications			
5.3 Applications for Review for Standard Practice			
5.4 Adverse events			
5.5 HREC application involving a new TCP			
5.6 Extraordinary applications			
**6.** Credentialing follow up			
**7.** Other Business			
**8.** Date of next meeting			

APPENDIX 29: REGISTER OF APPLICATIONS

**1. APPLICATION**		
**Application No.**	**08001V**	
**Title**	XYZ scanner	
**Submission date**	29/01/2008	
**Application completed correctly at first submission (Yes=1 No=0)**	0	
**Meeting date**	08/02/2008	
**Lead Contact Person**	Dr John Smith	
**Position**	Director of CT	
**Location**	MMC	
**Phone**	9594 7576	
**Email**	johnsmith@southernhealth.org.au	
**Department/Unit**	Diagnostic Imaging	
**Present at meeting (Yes=1 No=0)**	1	
**Head of Department/Unit**	Prof Mary Brown	
**Email**	marybrown@med.monash.edu.au	
**Present at meeting (Yes=1 No=0)**	1	
**Program**	Specialty Program	
**Program Director**	Prof Lee Chang	
**Present at meeting (Yes=1 No=0)**	0	
**Additional Program Director**	N/A	
**Additional Program Director present (Yes=1 No=0)**	N/A	
**2. APPROVAL**		
**Approved by TCPC (Yes=1 No=0)**	1	
**Approved by VPACT (Yes=1 No=0)**	1	
**Outcome letter sent (Yes=1 No=0)**	1	
**Appeal made to CE if not approved (Yes=1 No=0)**	N/A	
**Outcome of appeal**	N/A	
**Conditions of approval required (Yes=1 No=0)**	1	
**Conditions of approval received (Yes=1 No=0)**	1	
**Date conditions of approval received**	15/04/2008	
**3. MONITORING**		
**Reporting and patient outcome data required (Yes=1 No=0)**	1	
**Jan - Jun progress report received (Yes=1 No=0) + year**	2008 - N/A	
**Jul - Dec progress report received (Yes=1 No=0) + year**	2008 - received on 22/1/2009	
**Jan - Jun progress report received (Yes=1 No=0) + year**		
**Jul - Dec progress report received (Yes=1 No=0) + year**		
**Jan - Jun progress report received (Yes=1 No=0) + year**		
**Jul - Dec progress report received (Yes=1 No=0) + year**		
**Jan - Jun patient outcome data received (Yes=1 No=0) + year**	2008 - N/A	
**Jul - Dec patient outcome data received (Yes=1 No=0) + year**	2008 - received on 22/1/2009	
**Jan - Jun patient outcome data received (Yes=1 No=0) + year**		
**Jul - Dec patient outcome data received (Yes=1 No=0) + year**		
**Outcome spreadsheet used (TCPC or own)**	TCPC	
**4. REVIEW**		
**Date of review**	July 2010	
**Approved by TCPC (Yes=1 No=0)**		
**Approved by VPACT (Yes=1 No=0)**		
**Outcome letter sent (Yes=1 No=0)**		
**Appeal made to CE if not approved (Yes=1 No=0)**		
**Outcome of appeal**		
**Conditions of approval required (Yes=1 No=0)**		
**Conditions of approval received (Yes=1 No=0)**		
**Date conditions of approval received**		

APPENDIX 30: REPORTING DATABASE

REPORTING DATABASE TEMPLATE

**APPLICATIONS**		**YEAR 1**		**YEAR 2**		**YEAR 3**	
Number #	Title	Jan – Jun<br>Due <August>	Jul – Dec<br>Due <Feb>	Jan – Jun<br>Due <August>	Jul – Dec<br>Due <Feb>	Jan – Jun<br>Due <August>	Jul – Dec<br>Due <Feb>
eg 001	Gastric Sleeving	SUBMITTED	DUE	DUE	DUE	REVIEW	

Technology/Clinical Practice Committee

Evaluation Report 2008

Establishment of systems and processes for the introduction, monitoring and reporting of technologies and clinical practices at Southern Health

icare | Southern Health

integrity • compassion • accountability • respect • excellence | Better Health for Our Community

EXECUTIVE SUMMARY

This is the first Evaluation Report of the Southern Health Technology/Clinical Practice Committee (TCPC) and consists of activities undertaken in 2008. This report includes background information and explanations of the outcome measures and will form the basis of a paper for publication in a peer reviewed journal. Future reports will be briefer, focusing only on outcomes and future planning based on results.

Definition of technologies and clinical practices

Technologies and clinical practices (TCPs) are defined as therapeutic interventions (including prostheses; implantable devices; high cost pharmaceuticals; medical, surgical or other clinical procedures) or diagnostic procedures that are considered by a reasonable body of clinical opinion to be significantly different from existing clinical practice.

Establishment of new systems and processes at Southern Health

With executive endorsement from Southern Health, the TCPC aimed to enhance the existing systems and processes by developing a framework that met the following needs of the organisation in the area of safe and appropriate introduction of technologies and clinical practices:

- Increased transparency in decision-making
- Clear decision-making criteria
- Timetables to allow sufficient time for the application and decision-making processes.

Subsequent to detailed analysis of best practice, and in line with the Department of Human Services (DHS) Guidance for TCPCs, a program was established for the development, implementation and evaluation of the following components:

- Governance of the TCPC
- Application process for introduction or change of use of TCPs
- Decision-making for introduction or change of use of TCPs and subsequent review
- Monitoring and reporting of newly introduced TCPs
- Administration of the process
- Resources

These components have been piloted and refined based on feedback from applicants, support staff and TCPC members.

Audience for the evaluation

The key audiences for the evaluation are the Southern Health TCPC, the Executive Management Team (EMT) and the Southern Health Board. Other stakeholders who may be interested in the results of this evaluation include the Australian Council of Healthcare Standards Surveyors, the DHS Health Technology Program and the Victorian Policy Advisory Committee on Clinical Practice and Technology (VPACT). It is planned that this evaluation report will be disseminated to these stakeholders on completion.

Evaluation plan

The evaluation plan is presented in Appendix 1.

Conclusion

This evaluation report highlights that the Southern Health TCPC achieved its aim of developing a framework that meets the needs of the organisation in the area of safe and appropriate introduction of TCPs. The evaluation also reveals areas of achievement and success as well as opportunities for improvement.

The updating of systems and processes for introduction of a new TCP complies with current best practice. In updating the systems and processes, the TCPC has achieved its aim of developing a framework that meets the needs of the organisation in the area of safe and appropriate introduction of TCPs. This achievement is reflected in recommendations by DHS to other health services and requests to utilise Southern Health resources and expertise.

Opportunities for improvement include further revision of the application form, enhancement of decision-making by increasing committee member attendance and applicant representation at committee meetings, encouragement of formal feedback from applicants and continuing data collection on the application process. Transparency of the TCPC processes will be improved by the inclusion of approved patient information on the Southern Health intranet.

In 2009 the TCPC will work towards streamlining the process of application by modularising the application forms and integrating them with documentation for other Southern Health Committees. The TCPC will also establish a review process for recently introduced TCPs at the conclusion of their two year restricted approval period. In partnership with other Victorian health services, the Southern Health TCPC hope to undertake a comparison of decision-making for new TCPs and contribute to the development of a database of this information to prevent duplication of applications reviewed in Victoria.

The Southern Health TCPC will continue to be transparent and accountable in all its processes and decision-making.

ESTABLISHMENT OF BEST PRACTICE

Does the Southern Health Technology/Clinical Practice Program match current best practice?

Evidence Mapping

Prior to the establishment of the new systems and processes for introduction of TCPs at Southern Health, the TCPC Secretariat undertook a mapping exercise to establish national and international standards of best practice. Several guides to establishing a TCPC or TCP Program were identified. These came from Australian Capital Territory (ACT) Health, Australian Safety and Efficacy Register of New Interventional Procedures – Surgery (ASERNIP-S), New South Wales (NSW) Health, New Zealand National Health Committee and the Victorian Department of Human Services (DHS)[1-5]. The mapping exercise highlighted that the DHS guidance covered most of the key areas outlined by the other organisations with the exception of informing local consumer health councils and networks of applications received and their outcomes (*See Appendix 2*).

During 2008, the Southern Health TCPC attempted to meet all the criteria in the best practice map and also introduced the following additional components to the program:

- Declarations of conflict of interest by applicant and decision makers
- Publication of a decision summary to ensure transparency of decision-making
- Applications for change of use to an existing TCP
- Review of TCPs with restricted approval at the conclusion of two years to assess requirement for further monitoring

Summary

The Southern Health TCP program complies with current best practice for the introduction of TCPs to a health service. The single exception to this is that we have not implemented a process for informing local consumer groups.

Action

The TCPC will continue to maintain the current high standards and will consult with the Consumer Representative and the Southern Health Consumer Advisory Committee regarding communication with consumer groups.

GOVERNANCE

Is the program transparent and accountable?

Publication of systems and processes

To ensure that the purpose and scope of the TCPC are transparent the Terms of Reference are made available on the internet http://www.mihsr.monash.org/cce/pdf/tcpc_tor.pdf. The Terms of Reference are authorised by the Chair of the TCPC and are due for review in January 2011.

To ensure transparency of decision-making, a detailed summary of the factors considered in applications for introduction of new TCPs and a brief summary of decisions regarding change of use of TCPs in current practice are also published on the internet.

This Evaluation Report will also be available on the TCPC webpage.

A suite of documents and resources to support the implementation of the updated systems and processes were developed in 2008 and made available on the TCPC webpage. Along with the Terms of Reference these include:

- Application protocol
- Application form for introduction of new TCPs
- Application form for change of use of TCPs in current practice
- Application form and instructions for submission to VPACT
- Patient information templates
- Finding the Evidence: Guide to the best available evidence to support introduction of New Technologies and Clinical Practices 2008 (Centre for Clinical Effectiveness workbook)
- Timetable of meeting dates and application deadlines
- Decision summaries for introduction of new TCPs
- Summary of decisions made about change of use applications
- Progress report template
- Patient outcomes audit template

To ensure that the TCPC is accountable the Terms of Reference stipulate that the committee will report to the Executive Management Team (EMT) and DHS detailing applications submitted, procedures approved, reviews of existing TCPs and monitoring of introduced TCPs. These reports are due every six months.

Attendance at meetings

During 2008 the TCPC convened nine out of the twelve meetings scheduled. It is a requirement of the committee that a quorum of four members be present if any decisions are to be made. Every meeting held in 2008 achieved this.

Two meetings were cancelled in 2008 as there were no applications submitted and no business to discuss and one meeting was cancelled due to committee members being called away at short notice and a quorum was not available to meet.

TABLE 1: TCPC MEMBER ATTENDANCE 2008

ROLE	NAME	MEETINGS ELIGIBLE IN 2008	MEETINGS ATTENDED IN 2008	MEETINGS WHERE FEEDBACK WAS PROVIDED WHEN UNABLE TO ATTEND
Chair	A/Prof Richard King	9	9	Data collection incomplete in 2008
Secretary	Dr Claire Harris	9	9	
Executive Sponsor	A/Prof Wayne Ramsey	9	3	
Legal/Ethics	Ms Malar Thiagarajan	9	6	
Operational/Financial	Dr Cate Kelly	9	7	
Consumer Representative	Dr Beverley Castleman	9	7	
Nursing Representative	A/Prof Kylie Ward<br>Ms Lynne Bickerstaff	9	KW – 3<br>LB – 1	
Surgery Representative	Mr Ton Tran	6	0	
Medical Representative	Prof Ian Meredith	6	0	

*Ms Lynne Bickerstaff (LB) represented A/Prof Kylie Ward (KW)

Reporting on Southern Health TCPC activities

As stated in the Terms of Reference the TCPC is required to operate within a reporting structure to ensure corporate and clinical governance. The TCPC is committed to reporting to EMT and DHS biannually. Reports contain details of applications submitted, approved and monitored.

To comply with the DHS requirements of six monthly reporting on all activities of Victorian Health Service TCPCs, the Southern Health TCPC drafted a formal report outlining new processes introduced for TCP applications. Decision summaries for all approved applications were also included. This was approved at the September meeting of the Southern Health EMT and forwarded to DHS in October.

Feedback received from Dr Paul Fennessy (Manager, Genetics and Health Technology Programs Branch) suggested that the report provided to DHS was informative and would be tabled at the next VPACT meeting (November 2008) for information and discussion.

Data for the July – December 2008 report are being collated and the report is due for submission in March 2009.

Summary

Transparency and accountability of the TCPC and its processes was accomplished in 2008. However it was difficult for several of the committee members to attend all the meetings and one meeting was cancelled as a quorum was not available. This issue needs to be addressed during 2009.

Action

Actions to address the inability of TCPC members to attend meetings include:

- Review of available dates and times, conflicting meetings, existing commitments, etc with change of meeting schedule if possible and/or change of membership
- Meetings where no applications are tendered will start half an hour later to accommodate member's availability
- Members unable to attend a meeting will be encouraged to provide feedback regarding agenda items at the time of an apology

APPLICATIONS

Is the application process and documentation being utilised? Are applicants happy with the process? Were all TCPs introduced at Southern Health captured in the application process?

The Southern Health TCPC considers applications for the following:

- Introduction of a new TCP that has not been performed at Southern Health and requires external funding prior to implementation
- Introduction of a new TCP that has not been performed at Southern Health and can be implemented within existing funding
- Change of use of a TCP in current practice at Southern Health

VPACT provides an annual funding round to Victorian health services for the introduction of new TCPs where the cost of implementing is greater than $250,000. The role of the Southern Health TCPC in this situation is to review and make recommendations on applications prior to submission to VPACT.

Applications for the introduction of a new TCP at Southern Health whether for VPACT or internal funding are considered based on the assessment of safety; effectiveness and cost effectiveness; operational requirements such as cost, capability and credentialing; considerations related to access, equity, legal and ethical issues; and review of patient information. The application forms require details in each of these categories.

The application process for a change of use to a TCP in current practice at Southern Health was introduced in October 2008 and is designed to identify potential risks for the patient, clinician and the organisation as a result of the change. This process is currently being piloted.

TCP applications received

All applications to the TCPC are required to be submitted two weeks prior to the predetermined meeting dates. This allows time to follow up with applicants regarding any omissions in content as well as providing the TCPC members a week to review the application prior to the meeting. The application deadlines are posted on the TCPC web page.

During 2008 the TCPC reviewed fifteen applications; five for the 2008-09 VPACT funding round, five for the introduction of a new TCP, and five for the change of use of a current TCP.

Data has been collected to determine how many applications were received, submitted by the set timelines and completed correctly at first submission. This data is outlined in Table 2.

In 2008 two thirds (10/15) of the applications received were submitted on time however eight of the fifteen required further information and amendments.

TABLE 2: APPLICATIONS RECEIVED IN 2008

APPLICATION	SUBMITTED ON TIME	COMPLETED CORRECTLY	APPROVAL		COMMENT
			SOUTHERN HEALTH	DHS	
08001V[*]	✓	✓	✓	✓	Minor amendments made for format and presentation only – Submitted to DHS
08002V	✓		✓		Additional information was sought from applicants and amendments were required for format and presentation – Submitted to DHS
08003V	✓		✓	✓	
08004V	✓		✓		
08005V					Application lacked sufficient information for the TCPC to make a decision – Not submitted to DHS
08007N[†]	✓		✓	N/A	Amendments were required for content, format and presentation.
08010N	N/A	N/A	✓	N/A	This application was tabled at a meeting as a reinstatement of use and did not require a full application.
08012N	✓		✓	N/A	Amendments were required for content, format and presentation.
08013N	✓		✓	N/A	
08014N	✓			N/A	

COU 1[#]	✓	✓	✓	N/A	All completed satisfactorily.
COU 2		✓	✓		
COU 3	✓	✓	✓		
COU 4		✓	✓		
COU 5		✓	✓		

[*] **V** = VPACT application, [†]**N** = Application for introduction of a new TCP, [#]**COU** = Application for change of use to TCP

Satisfaction with the application process

2008 was a pilot phase for the new systems and processes introduced by the TCPC. To obtain feedback from applicants surveys were included with all application forms. Feedback was requested for 'content and wording' and 'format' of the application form, 'resources' provided for completion of the application form and 'assistance' available for the application process.

The TCPC received feedback from two of the eleven applicants who submitted an application for introduction of a new TCP in 2008. One applicant commented that the information on who to contact to complete various sections was useful. The applicant also felt that the space provided for answers in the application form confined the response able to be given and that all questions should not be tick boxes as applicants need room to answer via prose.

In addition to the feedback surveys, the TCPC convened a meeting in March 2008 for those who participated in the VPACT funding round. Items discussed at the meeting included internal timelines, the application form, the support process, other support/input/approval required, and sign off for applications. A number of suggestions for possible improvements were made.

Several changes were made for the 2009-10 VPACT funding round:

- Timelines revised
    - Application process brought forward to September
    - Deadlines introduced for contacting and receiving support from Coding, Clinical Information Management, the Centre for Clinical Effectiveness and Finance
    - Inclusion of instructions in the application form regarding deadlines for support services
- Sign off from Southern Health Finance Department required
- Invitation extended to EMT members to participate in the TCPC meetings when VPACT applications are discussed
- Consistency created between application forms for internal and external funding

Other changes suggested but not yet implemented include:

- Organising a 'library' of applications that would be ready to submit once the funding round had opened
- Informing other health services of Southern Health's earlier timelines for VPACT applications
- Utilising coding data for TCP Program evaluation

Change of use applications had the same feedback form attached. The TCPC received feedback from three of the five applications submitted. The feedback was positive and has been taken into consideration at each revision of the application form (Figure 1).

FIGURE 1: CHANGE OF USE FORM FEEDBACK RECEIVED IN 2008

'CONTENT AND WORDING'

- Pretty straight forward
- Easy to fill in
- No question included to ask if the TCP has TGA approval, does not ask if a similar device is in current use

'FORMAT'

- Would be better to automatically populate the first few fields
- Very straight forward
- Tick boxes were easy, form is difficult to use as a word document

'OTHER COMMENTS'

- Form does not ask purpose for the technology

Feedback was also received from TCPC members regarding usability and formatting of the change of use application form. This resulted in the following amendments:

- Inclusion of the date for when the change of use was endorsed by the Head of Department/Unit
- Addition of 'increased scope' as an option for nature of change of use
- Revision of questions to incorporate Yes-No as the answer options

- Inclusion of endorsement by appropriate Executive Director (Acute, Continuing Care, Mental Health) for related expenses or additional resources
- Inclusion of a decision summary
- Inclusion of a text response for provision of a brief summary of the change of use

Comparison of the Southern Health TCPC application process and decision-making with other health services

In 2008 DHS planned to invite all Victorian TCPCs to a meeting to discuss the application process for introduction of TCPs. It was hoped that the Southern Health TCPC would utilise this meeting to establish networks to share information and find out what applications other TCPCs had received, decisions they had made and how this compared to our own applications and decisions. No data for 2008 is available as the meeting was postponed and is due to take place in 2009.

Capturing TCPs that were introduced into practice at Southern Health but not reviewed by the TCPC

To determine if the new TCPC systems and processes have been effective we have developed strategies to identify new technologies or clinical practices that have been introduced without approval from the Southern Health TCPC. These will be undertaken in 2009.

Summary

The process and documentation have been revised during 2008 based on feedback and ongoing evaluation.

Timelines for submitting applications were met by most applicants in 2008 and although applications for change of use were completed correctly we found that applications for introduction of a TCP lacked information that committee members required to make an informed decision.

Action

Further work is required in 2009 to compare our processes and decision-making with other health services as well as establishing methods of capturing the introduction of TCPs or changes to the current use of TCPs that are not approved by the Southern Health TCPC. The TCPC will also continue to revise the application form.

DECISION-MAKING

Are processes and documentation for decision-making being utilised?

Decision summaries were introduced into the TCPC decision making process in March 2008. Their aim is to provide transparency for Southern Health and external stakeholders on all decisions made by the TCPC for applications submitted.

The decision summary includes details about conflict of interest; safety, effectiveness, cost, clinical feasibility, patient information and consent, access and equity, legal and ethical implications, and conditions of approval.

Recommendations for approval are made with the following qualifications:

- Recommended: Approved with no further need for assessment
- Restricted Recommendation – Audit: Approval subject to implementation under audit conditions. Conditions are specific to the technology or clinical practice being introduced
- Restricted Recommendation – Clinical Trial: Endorsed, however approval subject to implementation in clinical trial with Southern Health Human Research and Ethics Committee approval.
- Restricted Recommendation – Operational Restrictions: Endorsed, however financial or operational restrictions apply
- Not Recommended

Appropriate representation for decision-making

For introduction of new TCPs, the TCPC requires attendance by the Applicant, Department/Unit Head, Program Director and, if for a high cost pharmaceutical, the Chair and Executive Officer of the Southern Health Therapeutics Committee. To ensure there is an independent perspective an additional Program Director is invited to attend and contribute to the decision.

Change of use applications do not require representation at TCPC meetings.

In 2008, ten applications were received for introduction of a TCP at Southern Health, nine of which required representation at the TCPC meeting. The Chair decided that one application did not require support from the applicant as it was a reinstatement of use. Seven applications were represented by at least two people, while two were represented by only one person. Only one was attended by an independent Program Director.

TABLE 3: APPLICANT ATTENDANCE IN 2008

ATTENDANCE	APPLICATIONS									
	08001V	08002V	08003V	08004V	08005V	08007N	08010N*	08012N	08013N	08014N
Applicant (proxy)	✓	✓†	(✓)†			✓	N/A	(✓)	(✓)†	
Head of Department/Unit (proxy)	✓			✓	✓		N/A			
Program Director (proxy)		✓	(✓)	✓	✓	✓	N/A			✓
Additional Program Director							N/A		✓	

* 08010N – did not require representation as this was an application for reinstatement of practice, † Denotes that applicant was the Head of Department/Unit

Utilisation of decision summaries

The TCPC utilised the decision summary for all applications submitted from March 2008, with the exception of the application for reinstatement of use. All applications reviewed for approval since March 2008 covered each criterion of the decision summary. Decision summaries for TCP applications can be found on the TCPC webpage http://www.mihsr.monash.org/cce/shtcp.html.

Decisions made regarding change of use of current TCPs are recorded on the application form and are also summarised on the TCPC webpage http://www.mihsr.monash.org/cce/pdf/cou_summaryofdecisions2008.pdf.

Informing applicants of decision-making outcomes

The process for informing applicants of decisions was inconsistent prior to September 2008. Four applications have been submitted since September 2008 and an outcome letter was forwarded informing applicants of the due dates for reporting and any special conditions related to the application.

Compliance with conditions of approval

Of the ten applications approved in 2008, notification was received that all conditions of approval had been met. Deadlines for compliance were not issued prior to September 2008. Adherence to deadlines will be audited in 2009.

Process of appeal for TCPC applications

At the November 2008 meeting it was noted that the Southern Health TCPC did not have a process for appeal. A decision was made that all appeals should be directed to the Chief Executive of Southern Health. Applicants are made aware of this process when issued with the outcome letter of the TCPC meeting. The TCPC has published this process in the procedure protocol. There were no appeals in 2008.

Decision-making for review of TCPs introduced

Newly introduced TCPs are monitored closely, however after some time will be considered to be “standard practice”. The Southern Health TCPC has decided to review TCPs two years after introduction to assess the need for continued monitoring, restricted practice or special conditions.

All TCPs introduced prior to 2008 were reviewed; details are in the Monitoring and Reporting section below. A more formal review process for recently introduced TCPs will be implemented in 2009.

Summary

The transparency of the TCPC decision-making process has improved with the introduction of the decision summary in 2008. Methods for documenting and communicating decisions and ensuring compliance have improved throughout the year. Participation by appropriate representatives in the decision-making process can still be improved

Action

For 2009 the TCPC Administrator has revised the administration process and register of applications to address the following areas which require improvement from 2008 processes:

- Requesting attendance of an additional Program Director for all applications for introduction of a TCP
- Improving Applicant, Head of Department/Unit, Program Director attendance
- Sending of outcome letters following TCPC meeting that includes a due date for meeting special conditions of the application
- Collecting dates for when special conditions of applications are met by the applicant

MONITORING AND REPORTING

Are monitoring and reporting processes being utilised? Are applicants happy with the process? Were patient outcomes as expected?

Since January 2008 there have been considerable changes to the process of monitoring and reporting of new TCPs at Southern Health. These new processes are based on guidance provided by DHS. The TCPC Secretariat has developed data collection tools and a reporting template to assist Southern Health applicants.

Applicants are required to complete six monthly progress reports and patient outcome data spreadsheets for the periods January – June and July – December for a minimum of two years. Patient outcome data is requested in the proforma provided by the TCPC however if applicants already have a system in place they are free to forward patient outcome data in that format. Reporting data are collated by the TCPC Administrator and reports are prepared for the Southern Health EMT and DHS.

Applications requiring reporting in 2008

In 2008 the TCPC requested reporting from applicants listed in Tables 4 and 5.

Progress reports were received by the due date from all applicants in the January – June period and from six of the nine applicants in the July – December reporting period.

The new TCP had not been implemented during the relevant reporting period for three of the approved applications. All but one completed the progress reports correctly. Two applicants did not submit a data collection spreadsheet. None of the applicants used their own patient outcome data collection tool.

Table 4: January – June Reporting

APPLICATIONS	RECEIVED BY DUE DATE 29/8/2008	PROGRESS REPORTING TEMPLATE COMPLETED CORRECTLY	TCPC PATIENT OUTCOME DATA SPREADSHEET UTILISED	APPLICANTS OWN PATIENT OUTCOME DATA COLLECTION TOOL UTILISED
06001N	✓	✓	N/A	N/A
06004N	✓	✓	N/A	N/A
07007N	✓	N/A – no patients seen	N/A	N/A
08007N	✓	✓	✓	N/A

Table 5: July – December Reporting

**APPLICATIONS**	**RECEIVED BY DUE DATE 27/2/2009**	**PROGRESS REPORTING TEMPLATE COMPLETED CORRECTLY**	**TCPC PATIENT OUTCOME DATA SPREADSHEET UTILISED**	**APPLICANTS OWN PATIENT OUTCOME DATA COLLECTION TOOL UTILISED**
06001N	✓	✓	✓	N/A
06004N	✗	✓	✓	N/A
07004N	✓	N/A – no patients seen	N/A	N/A
07007N	✗	N/A – no patients seen	N/A	N/A
08001V	✓	✓	✓	N/A
08003V	✗	✓	✓	N/A
08007N	✓	✓	✓	N/A
08012N	✓	N/A – no patients seen	N/A	N/A
08013N	✓	✓	✓	N/A

Available data for comparison between original application and progress report data

Table 6 compares the number of patients actually treated with the number expected at the time of application. It should be noted that expected number of procedures to be performed is estimated per year, while actual number of procedures performed is collected six monthly. Underperformance should take into consideration that not all TCPs were introduced

at the beginning of reporting periods eg commencement of a procedure in October will impact on achieving expected number of performed procedures.

Some procedures exceeded the number expected and others did not reach the anticipated figure. No data were collected on the possible reasons for any discrepancies, so no firm conclusions can be drawn from this information.

There was one reported death, but this was not attributed to the procedure. The patient had neutropenia and thrombocytopenia due to myelodysplastic syndrome and died of sepsis of unknown origin. Six patients suffered adverse events including cerebral oedema, confusion and minor stroke, nosocomial infection, rejection of transplant and two unplanned readmissions post discharge. None of these adverse events were notified to the committee at the time.

Table 6: Reporting data 2008

**APPLICATIONS & REPORTING PERIODS**		**DATA**					
		**PATIENTS**		**PROCEDURES PERFORMED**		**DEATHS**	**OTHER ADVERSE EVENTS**
		**REFERRED**	**TREATED**	**EXPECTED (ANNUAL)**	**ACTUAL (6MONTHS)**		
06001N	October 2006 – June 2008	14	14	25	14	1	0
	July – December 2008	4	4	25	4	0	0
06004N	January – June 2008	10	7	10-20	7	0	0
	July – December 2008	10	9	6-8	9	0	3
07004N	July – December 2008	0	0	2-3	0	0	0
07007N	January – June 2008	0	0	10-15	0	0	0
	July – December 2008	0	0	10-15	0	0	0
08001V	July – December 2008	4	2	12	2	0	3
08003V	July – December 2008	270	270	5760	270	0	0
08007N	January – June 2008	10	3	6-10	3	0	0
	July – December 2008	12	9	6	9	0	0
08012N	July – December 2008	0	0	50-70	0	0	0
08013N	July – December 2008	7	7	200	7	0	0

Monitoring of TCP applications submitted to TCPC during 2001 – 2007

The TCPC undertook a review of all applications approved prior to 2008 and requested information from previous applicants regarding the current status of the TCP. The results are as follows:

TABLE 7: RESULTS OF REVIEW OF 2001 – 2007 TCP APPLICATIONS

	**VPACT**	**INTERNAL FUNDING**	**TOTAL**
Applications received	2	22	24
Applications approved	2	17	19
TCPs no longer in practice	0	4	4/19 (21%)
TCPs now considered to be routine practice	2	8	10/19 (53%)
TCPs still requiring monitoring and reporting	0	5	5/19 (26%)

Satisfaction with the reporting process

The survey feedback forms were also included with all Progress Report templates. Feedback about the reporting process was requested for 'content and wording', 'format of the progress report' and 'format of the outcome spreadsheet' (Figure 2). Three responses were received and all highlighted the need for revision of the forms which were seen as repetitive and difficult to use.

FIGURE 2: REPORTING FEEDBACK RECEIVED IN 2008

'CONTENT AND WORDING'

What worked well? Why?

- Minimal experience in using thus far as no cases used the new procedure
- Reasonably brief

What didn't work well? Why?

- The reporting of patients not treated was unexpected and therefore data had not been collected
- Questions repetitive particularly when considering the outcome spreadsheet as well. The same thing is asked three times

Should anything else be included?

- I do not see why we have to report on patients that do not undergo the procedure
- Additional measures specific to the TCP

Should anything be excluded?

- The repeated questions on infections and adverse outcomes, just one would do

'FORMAT OF THE PROGRESS REPORT'

What worked well? Why?

- Reasonably brief

What didn't work well? Why?

- Fields to fill in are not very easy to use. Would suggest using radio buttons for Yes/No responses
- Repeated questions

'FORMAT OF THE OUTCOME SPREADSHEET'

What worked well? Why?

- Reasonably brief

What didn't work well? Why?

- There is no need to summarise the data on this form, when we have to do a separate report on this form
- Some columns not relevant and were deleted, Formula didn't work on my computer so I re-did it

How could we improve the format?

- I have added some relevant outcome measures

Summary

Overall, compliance with the proposed reporting schedule was satisfactory. Applicants correctly completed progress reports and all utilised the templates developed by the Secretariat. Some adverse events were identified in the routine data collection cycle but were not reported to the committee at the time of the incident.

Action

In 2009 the TCPC will action suggestions made by applicants regarding progress reporting. The TCPC will continue to collect progress reports and outcome data on a six monthly basis in accordance with DHS requirements. The administrator will report on adverse events collected from the six monthly patient outcomes reporting, however the committee must address lack of reporting of adverse events as they arise.

RESOURCES

Are resource documents and support systems being utilised?

Southern Health staff from various disciplines are available to assist in the completion of applications. Resources are available from Health Information Services, Clinical Information Management, the Centre for Clinical Effectiveness, Medical Support Unit and Finance. These resources are advertised on the TCPC webpage and contact details are included in the application form.

Patient Information

It is a requirement of the TCPC that all newly introduced TCPs have an evidence-based patient information sheet. To assist applicants, the TCPC initially developed a patient information template which was based on the Southern Health Human Research and Ethics Committee template. In consultation with the TCPC Consumer Representative, all research aspects of the template were removed and an amended version was made available on the TCPC webpage.

In September 2008 a decision was made to reformat the template to match a suite of patient information brochures purchased by Southern Health from the Royal Australasian College of Surgeons for surgical procedures. The previously

approved patient information was reformatted and submitted to the Patient Information Committee for approval and uploading onto the Southern Health intranet.

It is planned that TCPC approved patient information will be available on the intranet in early 2009.

Applicant satisfaction with quality of resources

Feedback provided by two applicants stated that staff from the Centre for Clinical Effectiveness were helpful in supporting the evidence component of their applications.

Applicant feedback regarding accessibility of resources

No feedback was provided regarding the accessibility of the resources and support offered in 2008.

External requests for Southern Health TCP Program resources and expertise

TCPC have been sought by the Peter McCallum Cancer Centre in Victoria and, on the recommendation of DHS, by Queensland Health and the South Eastern Sydney and Illawarra Area Health Service.

Queensland Health has also asked that the TCPC Executive Officer conduct a workshop on establishing a TCP Program in 2009.

Summary

Due to the lack of feedback data, it is unclear whether or not the users of the Southern Health TCP Program found the resources and support useful. The Southern Health documents, processes and expertise are being sought by other health services and state health departments.

Action

In 2009 the TCPC plan to continue to request and collect feedback from applicants regarding the usefulness of the TCPC resources.

The TCPC will also liaise with the Quality Unit to ensure that the TCPC approved patient information is uploaded onto the Southern Health intranet.

REFERENCES

1. VICTORIAN DEPARTMENT OF HUMAN SERVICES (2006) Guidance for Victorian Health Services to Establish Technology/Clinical Practice Committees. Melbourne, Victoria, Victorian Department of Human Services.
2. AUSTRALIAN SAFETY AND EFFICACY REGISTER OF NEW INTERVENTIONAL PROCEDURES General Guidelines for Assessing, Approving and Introducing New Procedures into a Hospital or Health Service. Royal Australian College of Surgeons.
3. NEW SOUTH WALES HEALTH (2005) Clinical Practice - Model Policy for Safe Introduction of New Interventional Procedures. North Sydney, NSW, NSW Health.
4. AUSTRALIAN CAPITAL TERRITORY HEALTH (2007) Policy: Introduction of new health technologies. Canberra, ACT, ACT Health.
5. NATIONAL HEALTH COMMITTEE (2005) Decision-Making about New Health Interventions: A Report to the New Zealand Minister of Health. New Zealand, National Health Committee New Zealand.

APPENDIX 1: EVALUATION PLAN

COMPONENTS	KEY EVALUATION QUESTIONS	SUCCESS MEASURES/INDICATORS (WHAT TO MEASURE)	METHOD OF DATA COLLECTION AND SOURCE *(WHERE & HOW TO FIND IT)*	WHEN TO BE…..	
				Collected	Reported
Establishment of best practice	Does the SH TCP Program match current best practice?	Current best practice – Evidence Mapping	Revise mapping exercise of State/National/International Sources	End of establishment phase:3-5 year intervals	
Governance	Is the process transparent and accountable?	Publication of TOR, procedure protocols, application deadlines, meeting dates	Review of TCPC website, Southern Health intranet	Annually	Annually
		Attendance at meetings	Attendance list	Monthly	Annually
		Feedback from TCPC re processes	TCPC meetings – review minutes	Annually	Annually
		Achieving reporting requirements (EMT/ DHS)	Reports sent	Biannually	Biannually
		Appropriateness of reporting to EMT & DHS	Feedback from EMT & DHS	Biannually	Biannually
Applications<br>New TCPs	Has an application process and documentation in accordance with DHS requirements been established and is it being utilised?<br>Are applicants happy with the process?	Number of applications received	Audit of TCP register	Monthly	Annually
		Number of applications completed correctly at first submission	Audit of TCP register	Monthly	Annually
		Applicant satisfaction with application process	Audit application feedback forms	Monthly	Annually
		Number of VPACT applications approved by DHS	DHS feedback	Monthly	Annually
		Compliance with the Southern Health VPACT schedule	Audit VPACT timetable	Annually	Annually
		Comparison with other health services<br>▪ number of applications received<br>▪ comparison of applications (same/different)<br>▪ were the same decisions made	Collect this information from the group that Paul Fennessey sets up	Annually	Annually
	Did we capture all TCPs introduced at Southern Health	Number of TCPs introduced at Southern Health that did not go through the TCPC process	Query unit managers and theatre<br>Query enquiries received by Steven Anderson/Kevin Ericson<br>Query high cost drug list produced by Pharmacy<br>Query presentations made at the Southern Health State of Art Lectures and grand rounds<br>Query Capital Expenditure "Unfunded Capital Expenditure budget process of prioritisation"	Quarterly	Annually
Applications	Has a change of use application	Number of applications received	Audit of TCP register	Monthly	Annually

COMPONENTS	KEY EVALUATION QUESTIONS	SUCCESS MEASURES/INDICATORS (WHAT TO MEASURE)	METHOD OF DATA COLLECTION AND SOURCE *(WHERE & HOW TO FIND IT)*	WHEN TO BE…..	
				Collected	Reported
COU of existing TCPs	process and documentation been established and is it being utilised?<br>Are applicants happy with the process?	Number of applications completed correctly at first submission	Audit of TCP register	Monthly	Annually
		Applicant satisfaction with COU application process	Audit application feedback forms	Monthly	Annually
Decision-making<br>New TCPs	Have processes and documentation for decision-making been established and are they being utilised?	Appropriate representation at TCPC meetings to discuss applications	Audit of minutes for attendance by applicant/HOD/Program Director	Monthly	Annually
		Number of applications that the TCPC utilised the decision summary for	Audit of application folders	Monthly	Annually
		Number of decision summaries published on the website	Cross check applications with webpage	Monthly	Annually
		Number of applicants that complied with the conditions of approval and were received by the due date	Audit of TCP register	Monthly	Annually
		Number of outcome letters for approval for provisional use sent	Audit of TCP register	Monthly	Annually
		Number of applicants that appealed to the Chief Executive re the TCPC decision	Audit of TCP register	Monthly	Annually
Decision-making<br>Review of approved TCPs	Have processes and documentation for decision-making for reviewed TCPs been established and are they being utilised?	Number of reviews that the TCPC utilised the decision summary for	Audit of application folders	Monthly	Annually
		Number of decision summaries published on the website	Cross check applications with webpage	Monthly	Annually
		Number of TCPs withdrawn after review	Audit of TCP register	Monthly	Annually
		Number of decisions made that were consistent with the evidence	Review of the evidence	Monthly	Annually
Monitoring and reporting for newly introduced TCPs	Have monitoring and reporting processes been established and are they being utilised?	Number of reports<br>▪ received<br>▪ received by due date<br>▪ received late	Audit of TCP register	Biannually	Annually
		Number of applicants who used TCPC outcome spreadsheet	Audit of TCP register	Biannually	Annually
		Number of applicants who used their own outcome data collection tool	Audit of TCP register	Biannually	Annually

COMPONENTS	KEY EVALUATION QUESTIONS	SUCCESS MEASURES/INDICATORS (WHAT TO MEASURE)	METHOD OF DATA COLLECTION AND SOURCE *(WHERE & HOW TO FIND IT)*	WHEN TO BE…..	
				Collected	Reported
	Are applicants happy with the process?<br>Were patient outcomes as expected?	Number of reporting templates completed correctly	Audit of TCP register	Biannually	Annually
		Applicant satisfaction with reporting processes	Audit application feedback forms	Biannually	Annually
		Number of procedures performed<br>Referred versus treated<br>Expected versus actual<br>Deaths<br>Other adverse events	Comparison between original applications and progress report data	Biannually	Biannually
Resources	Has a support system and resource documents been developed and are these being utilised?	Number of applicants that utilised patient information template	Audit of application documents	Biannually	Annually
		Applicant satisfaction with quality and accessibility of resources	Audit application feedback forms	Monthly	Annually
		Feedback from resource providers	Via formal meeting or request feedback via email	Biannually	Annually
		Number of requests for use of resources/expertise externally	Audit of requests	Annually	Annually

APPENDIX 2: MAPPING EXERCISE

BEST PRACTICE CRITERIA MAPPING FOR SOUTHERN HEALTH TECHNOLOGY AND CLINICAL PRACTICE PROGRAM	VICTORIAN DHS[1]	ASERNIP-S[2]	NSW HEALTH[3]	ACT HEALTH[4]	NEW ZEALAND[5]	SOUTHERN HEALTH	
						BEFORE	AFTER
Principles underpinning the safe introduction of a TCP							
A TCP committee is established	✓	✓	✓	✓	✓	✓	✓
Any conflicts of interests are disclosed	✓	✓	✓				✓
Safety of new TCP is established							✓✸
Evidence concerning a new TCP is robust and reliable	✓	✓	✓	✓	✓		✓
Resources required and future/recurring costs of the TCP are estimated as accurately as possible	✓	✓	✓	✓	✓		✓
Ethics procedures are in place to protect patients, clinicians and the community	✓	✓	✓		✓	✓	✓
Issues of access and equity are considered							✓✸
Legislative requirements are met							✓✸
Risk management procedures are in place	✓		✓	✓			✓
Patient information and informed consent procedures are established	✓	✓	✓			✓	✓
Evidence-based practice informs conditions and logistics for introduction	✓		✓	✓	✓		✓
Appropriate, credentialed and trained staff are in place for the introduction of the new TCP	✓	✓	✓	✓	✓	✓	✓
Appropriate clinical and physical infrastructure/facilities exist to support the introduction of new TCP	✓	✓	✓	✓	✓		✓
Recommendations for introduction have clearly noted conditions eg audit, clinical trial, operational restrictions							✓✸
TCP committee responsibilities							
TCP committee meetings are held at regular intervals	✓						✓
There is a range of clinical disciplines represented on the TCP committee	✓	✓		✓	✓	✓	✓
There is a consumer representative on the TCP committee	✓					✓	✓
There are established criteria for assessment of applications to introduce a new TCP	✓					✓	✓
Clinical and financial effects of each TCP are considered at all levels and in all departments	✓						✓
Decisions of the committee are published to ensure transparency and accountability							✓✸
A register of applications and approved procedures is maintained	✓		✓			✓	✓
Information about the TCP is disseminated and advice provided	✓	✓	✓		✓	✓	✓
Appropriate training is provided to all staff so that each TCP is performed (and all equipment is handled) safely	✓		✓			✓	✓
Determine processes for monitoring and reviewing existing TCP	✓		✓				✓
Monitor requirements for each approved TCP	✓	✓	✓	✓	✓		✓
Any adverse event occurring with an approved TCP is notified to the TCP committee	✓	✓	✓			✓	✓
The TCP committee operates within a reporting structure to ensure corporate and clinical governance	✓	✓				✓	✓
Six monthly reports are submitted to the state health department detailing applications, approved procedures, reviews	✓		✓				✓

of existing TCP and monitoring of introduced/referred TCP							
Six monthly reports are submitted to the health service executive	✓		✓				✓
TCP Application							
Clinician and/or unit making the application will receive endorsement from their departmental head	✓		✓			✓	✓
Completed application will be forwarded to the chair of the TCP committee or other nominated delegate	✓		✓			✓	✓
Reassessment of newly introduced TCPs at the end of the two year monitoring period							✓❋
Local consumer health councils and networks will be informed of applications and of their outcomes			✓				✓
Resources							
Expertise in coding, data analysis, evidence review, finance and credentialing provided							✓❋
Guide to finding the evidence of effectiveness of TCP to support the application							✓❋
Template for Patient information brochure							✓❋
Templates for data collection tools and reporting proformas							✓❋

❋ Additional items introduced by Southern Health

Technology/Clinical Practice Committee

Evaluation Report 2009

Establishment and maintenance of systems and processes for the introduction, monitoring and reporting of technologies and clinical practices at Southern Health

icare
integrity • compassion • accountability • respect • excellence

Southern Health
Better Health for Our Community

EXECUTIVE SUMMARY

This is the second Evaluation Report of the Southern Health Technology/Clinical Practice Committee (TCPC) and consists of activities undertaken in 2009. This report is intended to be brief, focusing only on outcomes and future planning based on results. Details of background information and explanations of the outcome measures are included in the 2008 Evaluation Report available at http://www.southernhealth.org.au/icms_docs/2159_Evaluation_Report_2008.pdf.

Definition of technologies and clinical practices

Technologies and clinical practices (TCPs) are defined as therapeutic interventions (including prostheses; implantable devices; high cost pharmaceuticals; medical, surgical or other clinical procedures) or diagnostic procedures that are considered by a reasonable body of clinical opinion to be significantly different from existing clinical practice.

Systems and processes at Southern Health

In 2009 the TCPC continued to refine and improve its systems and processes. Two new components were introduced and are currently being piloted. These components include; the review (taken two years after implementation) of recently introduced TCPs to determine if they can be classified as standard practice at Southern Health or if further monitoring and reporting is required and the Quality Assurance requirements for clinical audit following implementation of a new TCP at Southern Health.

Audience for the evaluation

The key audiences for the evaluation are the Southern Health TCPC, the Executive Management Team (EMT) and the Southern Health Board. Other stakeholders who may be interested in the results of this evaluation include the Australian Council of Healthcare Standards Surveyors, the DHS Health Technology Program and the Victorian Policy Advisory Committee on Clinical Practice and Technology (VPACT). It is planned that this evaluation report will be disseminated to these stakeholders on completion.

Evaluation plan

The evaluation plan is presented in Appendix 1.

Conclusion

This evaluation report highlights that the Southern Health TCPC is continuing to achieve its goal of meeting the needs of the organisation in the area of safe and appropriate introduction of TCPs. The 2009 evaluation has revealed areas of achievement and success as well as opportunities for improvement.

The greatest achievement of the Technology/Clinical Practice Program for 2009 was receiving the Australian Council of Healthcare Standards Quality Improvement Award for Non-Clinical Service Delivery.

The Committees systems and processes continue to comply with best practice and this is reflected in the requests received from South Eastern Sydney and Illawarra Area Health Service and Queensland Health to access Southern Health TCPC documents.

Compared with 2008 data the Committee noticed improved attendance at meetings in 2009 by its members as well as applicant representation. The Committee was also successful in establishing a review process for recently introduced TCPs at the conclusion of their two year restricted approval period.

Opportunities for improvement include further revision of the Quality Assurance process, review of six monthly reporting for any adverse events and nomination of a new Executive Sponsor and a delegate for the Southern Health Director of Nursing and Midwifery.

In 2010 the TCPC will continue to work towards streamlining the process of application by modularising the application forms and integrating them with documentation for other Southern Health Committees. The Committee will also look at appropriate methods to ensure that all adverse events related to the newly introduced TCPs are reported to the Committee at the time they occur.

In 2008 the Committee attempted to undertake a comparison of decision-making data for new TCPs introduced by other Victorian health services and contribute this information to the development of a database to prevent duplication of applications reviewed in Victoria. This information was requested from the Department of Health in 2009 but at the time the report was completed had not been received. The Committee will continue to request this data from the Department of Health in 2010.

The Southern Health TCPC will continue to be transparent and accountable in all its processes and decision-making.

ESTABLISHMENT OF BEST PRACTICE

Does the Southern Health Technology/Clinical Practice Program match current best practice?

Evidence Mapping

During 2009, the Southern Health TCPC continued to meet all the criteria in the 2008 best practice map.

The Southern Health Technology Clinical Practice Program won the 2009 Australian Council of Healthcare Standards Quality Improvement Award for Non-Clinical Service Delivery and was also nominated for the 2009 Victorian Public Healthcare Award for Most Appropriate Care: providing least intrusive and earliest effective care.

Action

A review of the best practice criteria for technology and clinical practice programs will be undertaken in 2011.

GOVERNANCE

Is the program transparent and accountable?

Publication of systems and processes

In 2009, the TCPC webpage moved to the new Southern Health website. Maintaining transparency and accountability the website accommodates the following suite of documents and resources to support the implementation of the systems and processes of the TCPC:

- TCPC Terms of Reference
- Procedure Protocol for the TCP Program at Southern Health
- Timetable of meeting dates and application deadlines
- Application form for introduction of new TCPs
- Application form for change of use of TCPs in current practice
- Application form for review for reclassification to standard practice
- Application form and instructions for submission to VPACT
- Patient information template
- Finding the evidence: Guide to the best available evidence to support introduction of New Technologies & Clinical Practices 2008 (Centre for Clinical Effectiveness workbook)
- Decision summaries for introduction of new TCPs
- Summary of decisions made about change of use applications
- Progress report template
- Patient outcomes audit template
- TCPC Program Logic Map 2008
- TCPC Evaluation Report 2008

In 2009, the TCPC submitted an application for Quality Assurance for generic data collection items in the Department of Health (DOH) Progress Reports. This application was approved by the Southern Health Human Research and Ethics Committee (HREC) in July 2009; however requires notification of individual clinical audits from newly approved applicants. The TCPC have prepared template letters for this purpose.

Attendance at meetings

During 2009 the TCPC convened eight out of the twelve meetings scheduled. It is a requirement of the committee that a quorum of four members be present if any decisions are to be made. Every meeting held in 2009 achieved this.

Four meetings were cancelled in 2009 as there were no applications submitted and no business to discuss and one meeting was postponed due to committee members being called away at short notice and a quorum was not available to meet.

Table 1: TCPC member attendance 2009

Role	Name	Meetings eligible in 2009	Meetings attended in 2009	Meetings where feedback was provided when unable to attend
Chair	A/Prof Richard King	8	7	1 (November)
Secretary	Dr Claire Harris	8	8	N/A
Executive Sponsor	A/Prof Wayne Ramsey	8	0	1 (May)
Legal/Ethics	Ms Malar Thiagarajan	8	7	0
Operational/Financial	Dr Cate Kelly	8	6	0
Consumer Representative	Dr Beverley Castleman	8	7	1 (July)
Consumer Representative	Ms Pamela Sloss	5	4	0
Nursing Representative	A/Prof Kylie Ward<br>Ms Anne Doherty<br>Mrs Chayne Chalmers	8	KW – 0<br>AD – 0<br>CC – 0	N/A
Surgery Representative	Mr Ton Tran	4	0	0
Medical Representative	Prof Ian Meredith	4	1	0
Surgery Representative	Mr Cliff Choong	7	7	N/A
Procurement	Mr Brendan Hoare	3	2	0

* A/prof Kylie Ward (kw) left southern health in march 2009, Ms Anne Doherty was acting nursing representative from march 2009, Ms Chayne Chalmers was invited to join the TCPC in October 2009 as the newly appointed director of nursing and midwifery at southern health.

Committee members who attended less than eight meetings joined the TCPC at different times of the year.

Reporting on Southern Health TCPC activities

During 2009, the TCPC provided the following reports to the southern health executive management team:

- July – December 2008 progress report
- January – June 2009 progress report
- TCPC 2008 evaluation report
- Notification of changes to VPACT 2010-2011 funding round

During 2009, the TCPC provided the following reports to the Victorian Department of Health:

- July – December 2008 progress report
- January – June 2009 progress report

Summary

Transparency and accountability of the TCPC and its processes was accomplished in 2009. The TCPC improved on the attendance of members in 2008 however it was still evident that surgical and medical representatives found meetings difficult to attend. In 2009 both the surgical and medical representatives were replaced to improve attendance. The TCPC was not successful in attracting a nursing representative in 2009 and did not have sufficient executive sponsorship; this should be addressed in 2010.

Action

Actions to address transparency of quality assurance requirements:

- Include information and templates on the TCPC webpage

Actions to address transparency of reporting to EMT and DOH:

- Once approved include all reports on the TCPC webpage

Actions to address nursing and executive sponsorship representation

- The committee should seek a delegate for Chayne Chalmers and a replacement for Wayne Ramsey.

Applications

Is the application process and documentation being utilised? Are applicants happy with the process? Were all TCPs introduced at Southern Health captured in the application process?

The review process for recently introduced TCPs for reclassification as standard practice at Southern Health was introduced in May 2009 and is designed to determine whether a recently introduced TCP requires further monitoring and reporting. This process is currently being piloted.

TCP applications received

During 2009 the TCPC reviewed fifteen applications;

- Three for the 2009-2010 VPACT funding round
- Four for the introduction of a new TCP
- Three for the change of use of a current TCP
- Two for reclassification as standard practice
- Three for the 2010-2011 VPACT funding round

As part of the 2010-2011 VPACT funding round the Committee also reviewed four expression of interest applications, all of which were submitted to the DoH for consideration.

Data has been collected to determine how many applications were received, submitted by the set timelines and completed correctly at first submission. This data is outlined in Table 2.

In 2009 only four out of the fifteen applications received were submitted on time with more than half (8/15) completed correctly.

Table 2: Applications received in 2009

**Application**	**Submitted on time**	**Completed correctly**	**Approval**		**Comment**
			**Southern Health**	**DoH**	
06001N	N/A	✓	✓		Both applicants assisted the TCPC in piloting the new forms for reclassification as standard practice
06004N	N/A	✓	✓		
09001V*			✓	✓	Additional information was sought from applicants and amendments were required for format and presentation – Submitted to DHS
09002V			✓	✓	
09003N†		✓	✓		
09004V			✓	✓	Additional information was sought from applicants and amendments were required for format and presentation – Submitted to DHS
09005N	✓	✓		N/A	
09007N	✓		✓	N/A	Amendments were required for content, format and presentation
09008N		✓	✓	N/A	
09009V			✓		At the time this evaluation report was drafted these application were still being considered by DoH
09010V			✓		
09011V			✓		
COU6#	✓	✓	✓	N/A	All completed satisfactorily
COU7		✓	✓	N/A	
COU8	✓	✓	✓	N/A	

* **V** = VPACT application, † **N** = Application for introduction of a new TCP, # **COU** = Application for change of use to TCP

Satisfaction with the application process

Feedback was requested for „content and wording‟ and „format‟ of the application form, „resources‟ provided for completion of the application form and „assistance‟ available for the application process.

The TCPC received feedback from two of the thirteen applicants who submitted an application to the TCPC in 2009.

One applicant commented that the application form for introduction of new technologies and clinical practices was well formatted and the „Finding the Evidence‟ workbook was a useful document.

Another applicant commented that the change of use form was easy and quick to complete.
The two applicants who piloted the review for reclassification form were happy with the process.

Comparison of the Southern Health TCPC application process and decision-making with other health services

It was hoped that the Southern Health TCPC would utilise a planned meeting by DoH for all Victorian health services to discuss the application processes for introduction of TCPs. The aim of the meeting would be to establish networks to share information and find out what applications other TCPCs had received, decisions they had made and how this compared to our own applications and decisions. Unfortunately this meeting did not eventuate in 2009 and instead information regarding applications received and approved by other health services was requested by Southern Health from DoH in mid 2009. At the time of this report Southern Health had not yet received a response regarding this request.

Capturing TCPs that were introduced into practice at Southern Health but not reviewed by the TCPC

To determine if the new TCPC systems and processes have been effective we have developed strategies to identify new technologies or clinical practices that have been introduced without approval from the Southern Health TCPC. These activities were not consistently undertaken in 2009. Effort will be made in 2010 to capture this information with the assistance of the coding department.

Summary

The process and documentation have been revised during 2009 based on feedback and ongoing evaluation.
Timelines for submitting applications were not well adhered to with only four of the thirteen applications being submitted on time. It was also found that only six of the thirteen applications for introduction of a new TCP or change of use of existing TCP were completed correctly at first submission.

Action

Further work is required in 2010 to compare our processes and decision-making with other health services as well as establishing methods of capturing the introduction of TCPs or changes to the current use of TCPs that are not approved by the Southern Health TCPC. The TCPC will also continue to revise the application form.

DECISION-MAKING

Are processes and documentation for decision-making being utilised?

Appropriate representation for decision-making

For introduction of new TCPs, the TCPC requires attendance by the Applicant, Department/Unit Head, Program Director and, if for a high cost pharmaceutical, the Chair and Executive Officer of the Southern Health Therapeutics Committee. To ensure there is an independent perspective an additional Program Director is invited to attend and contribute to the decision.

Change of use applications do not require representation at TCPC meetings.

In 2009, ten applications were received for introduction of a TCP at Southern Health, nine of which required representation at the TCPC meeting. The Chair decided that one application did not require support from the applicant as it had previously been approved by Southern Health for submission to VPACT but rejected by DoH. Eight applications were represented by two or more people and seven were attended by an independent Program Director.

Table 3: Applicant attendance in 2008

Attendance	Applications									
	09001V	09002V	09003N	09004V	09005N	09007N	09008N*	09009V	09010V	09011V
Applicant (proxy)	✓	✓†	✓†	✓†		✓†	✓	✓	N/A	✓
Head of Department/Unit (proxy)					(✓)				N/A	
Program Director (proxy)	✓	✓	✓			✓	✓	✓	N/A	
Additional Program Director	✓		✓		✓	✓	✓	✓	N/A	✓

† Denotes that applicant was the Head of Department/Unit, (✓) denotes proxy representative.

Utilisation of decision summaries

The TCPC utilised the decision summary for nine of the ten applications submitted in 2009, the exception being a VPACT application led by another health service. Decision summaries for TCP applications can be found on the TCPC webpage http://www.southernhealth.org.au/page/Health_Professionals/TCPC/.

Decisions made regarding change of use of current TCPs are recorded on the application form and are also summarised on the TCPC webpage.

Informing applicants of decision-making outcomes

All 2009 applicants have been informed of the decision made by the Committee regarding their application. All applicants received an outcome letter outlining the due dates for reporting and any special conditions related to the application.

Compliance with conditions of approval

Conditions of approval were met by five of the six eligible applications approved in 2009. Special conditions for the three VPACT application approved by the TCPC are not due until 2010.

Process of appeal for TCPC applications

The one application rejected by the TCPC in 2009 did not appeal the decision with the Southern Health Chief Executive.

Decision-making for review of TCPs introduced

In 2009 the TCPC reviewed and approved two applications (introduced at Southern Health in 2006) for reclassification as standard practice. Summaries of the decisions made are available on the TCPC webpage.

Summary

Participation by appropriate representatives in the decision-making process has vastly improved during 2009. All decisions made by the TCPC in 2009 were transparent and freely available on the Committee's webpage.

Action

No specific actions outlined for 2010.

MONITORING AND REPORTING

Are monitoring and reporting processes being utilised? Are applicants happy with the process? Were patient outcomes as expected?

Applications requiring reporting in 2009

In 2009 the TCPC requested reporting from applicants listed in Tables 4 and 5.

Progress reports were received by the due date from all applicants in the January – June period and from two of the eight applicants in the July – December reporting period. Most applicants notified the TCPC secretariat that they would have difficulty in submitting reports by the due date as it coincided with Southern Health Clinical Accreditation.

In the January – June period one applicant did not submit a data collection spreadsheet and no applicants used their own patient outcome data collection tool. In the July – December period one applicant used their own data collection spreadsheet.

Table 4: January – June Reporting

Applications	Received by Due Date 29/8/2009	Progress reporting template completed correctly	TCPC patient outcome data spreadsheet utilised	Applicants own patient outcome data collection tool utilised
07004N	✓	N/A – no patients seen	N/A	N/A
07007N	✓	N/A – no patients seen	N/A	N/A
08001V	✓	✓	✓	N/A
08003V	✓	✓	✓	N/A
08007N	✓	✓	✓	N/A
080012N	✓	✓	✓	N/A
080013N	✓	✓	N/A	N/A

Table 5: July – December Reporting

Applications	Received by Due Date 26/02/2010	Progress reporting template completed correctly	TCPC patient outcome data spreadsheet utilised	Applicants own patient outcome data collection tool utilised
07007N	x	N/A	N/A	N/A
08001V	x	✓	✓	N/A
08003V	✓	✓	✓	N/A
08007N	x	✓	✓	N/A
080012N	✓	x	N/A	✓
080013N	x	✓	✓	N/A
09008N	x	✓	✓	N/A

* At the time this evaluation report was completed no information was received for this application to indicate if any patients had been treated with the TCP

Available data for comparison between original application and progress report data

Table 6 compares the number of patients actually treated with the number expected at the time of application. It should be noted that expected number of procedures to be performed is estimated per year, while actual number of procedures performed is collected six monthly. Underperformance should take into consideration that not all TCPs were introduced at the beginning of reporting periods eg commencement of a procedure in November will impact on achieving expected number of performed procedures.

Some procedures exceeded the number expected and others did not reach the anticipated figure. No data were collected on the possible reasons for any discrepancies, so no firm conclusions can be drawn from this information.

There were three reported deaths, none of which were attributable to the treatment. Thirty-eight patients suffered adverse events including twelve cases of catheter related bacteraemia, twenty-two cases of blocked catheters and four cases of unplanned readmission post discharge.

Table 6: Reporting data 2008

Applications & Reporting Periods		Data					
				Procedures performed		Deaths	Other adverse events
		Referred	Treated	Expected (annual)	Actual (6 months)		
07004N	January – June 2009	0	0	2-3	0	0	0
07007N	January – June 2009	0	0	10-15	0	0	0
08001V	January – June 2009	6	0	2	0	0	0
	July – December 2009	3	1	2	1	0	0
08003V	January – June 2009	393	393	600	393	0	0
	July – December 2009	655	655	420	655	0	0
08007N	January – June 2009	12	5	6	5	0	0
	July – December 2009	9	3	8	3	0	0
08012N	January – June 2009	14	19	50-70	14	0	14
	July – December 2009	70	70	30-40	70	3	24
08013N	January – June 2009	23	23	20	23	0	0
	July – December 2009	24	24	20	24	0	0
09008N	July – December 2008	12	3	5	3	0	0

Satisfaction with the reporting process

Feedback about the reporting process was requested for 'content and wording', 'format of the progress report' and 'format of the outcome spreadsheet'. Two responses were received and highlighted that the pre-filled format was helpful and requirements for short answers worked well. These applicants also felt that applying specific information to a generic form was not difficult and that the questions in the progress report and patient outcome spreadsheet were repetitive.

When asked what could be improved one applicant suggested inclusion of information specific to the technology and the other suggested the inclusion of a section for comments on the relative quality of the patient outcomes.

Quality Assurance supplements submitted to HREC

Two applications approved by the TCPC in 2009 were required to submit a supplement to HREC for the TCPC Quality Assurance application. At the conclusion of 2009 these supplements had not yet been submitted.

Summary

Overall, compliance with the proposed reporting schedule was satisfactory. Some adverse events were identified in the routine data collection cycle but were not reported to the committee at the time of the incident. This will require attention from the Committee in 2010.

Action

Reporting

- Committee to review six monthly reporting for any adverse events
- Committee to decide on actions to ensure that adverse events are reported at the time they occur

Quality Assurance

- Follow up with applications that are required to submit a supplement to the TCPC Quality Assurance application

RESOURCES

Are resource documents and support systems being utilised?

Patient Information

In 2009, TCPC approved patient information was made available on the Southern Health Intranet. The patient information template has been utilised by four out of nine approved applications. Five of the nine applications have not yet commenced and the Committee is awaiting patient information as a condition of approval.

Applicant satisfaction with quality of resources

No feedback was received regarding the quality of resources is 2009.

Applicant feedback regarding accessibility of resources

No feedback was received regarding the accessibility of the resources and support offered in 2009. However one applicant commented that the 'Finding the Evidence' workbook was a useful document.

External requests for Southern Health TCP Program resources and expertise

During 2009 the South Eastern Sydney and Illawarra Area Health Service and Queensland Health requested access to the Southern Health TCPC documents.

Representatives from Queensland Health met with the Executive Officer of the TCPC in February 2009 and sought advice on how to establish a health service wide TCP Program. In September 2009, the Executive Officer of the Southern Health TCPC conducted a workshop for Queensland Health on establishing a TCP Program.

Summary

Due to the lack of feedback data, it is unclear whether or not the users of the Southern Health TCP Program found the resources and support useful. The Southern Health documents, processes and expertise are being sought by other health services and state health departments.

Action

In 2010 the TCPC plan to continue to request and collect feedback from applicants regarding the usefulness of the TCPC resources. The TCPC will also update the 'Finding the Evidence' workbook.

REFERENCES

1. Victorian Department Of Human Services (2006) Guidance for Victorian Health Services to Establish Technology/Clinical Practice Committees. Melbourne, Victoria, Victorian Department of Human Services.
2. Australian Safety And Efficacy Register Of New Interventional Procedures General Guidelines for Assessing, Approving and Introducing New Procedures into a Hospital or Health Service. Royal Australian College of Surgeons.
3. New South Wales Health (2005) Clinical Practice - Model Policy for Safe Introduction of New Interventional Procedures. North Sydney, NSW, NSW Health.
4. Australian Capital Territory Health (2007) Policy: Introduction of new health technologies. Canberra, ACT, ACT Health.
5. National Health Committee (2005) Decision-Making about New Health Interventions: A Report to the New Zealand Minister of Health. New Zealand, National Health Committee New Zealand.

Appendix 1: Evaluation Plan

Components	Key evaluation questions	Success measures/indicators (What to measure)	Method of data collection and source (Where and how to find it)	When to be… Collected	Reported
Establishment of best practice	Does the SH TCP Program match current best practice?	Current best practice – Evidence Mapping	Revise mapping exercise of State/National/International Sources	End of establishment phase – 3 to 5 year intervals	
Governance	Is the process transparent and accountable?	Publication of TOR, procedure protocols, application deadlines, meeting dates	Review of TCPC website, Southern Health intranet	Annually	Annually
		Attendance at meetings	Attendance list	Monthly	Annually
		Feedback from TCPC re processes	TCPC meetings – review minutes	Annually	Annually
		Achieving reporting requirements (EMT/ DHS)	Reports sent	Biannually	Biannually
		Appropriateness of reporting to EMT & DHS	Feedback from EMT & DHS	Biannually	Biannually
Applications<br>New TCPs	Has an application process and documentation in accordance with DHS requirements been established and is it being utilised?<br>Are applicants happy with the process?	Number of applications received	Audit of TCP register	Monthly	Annually
		Number of applications completed correctly at first submission	Audit of TCP register	Monthly	Annually
		Applicant satisfaction with application process	Audit application feedback forms	Monthly	Annually
		Number of VPACT applications approved by DHS	DHS feedback	Monthly	Annually
		Compliance with the Southern Health VPACT schedule	Audit VPACT timetable	Annually	Annually
		Comparison with other health services<br>number of applications received<br>comparison of applications (same/different)<br>were the same decisions made	Collect this information from the group that Paul Fennessey sets up	Annually	Annually
	Did we capture all TCPs introduced at Southern Health	Number of TCPs introduced at Southern Health that did not go through the TCPC process	Query unit managers and theatre<br>Query enquiries received by Steven Anderson/Kevin Ericson<br>Query high cost drug list produced by Pharmacy<br>Query presentations made at the Southern Health State of Art Lectures and grand rounds<br>Query Capital Expenditure “Unfunded Capital Expenditure budget process of	Quarterly	Annually

Components	Key evaluation questions	Success measures/indicators (What to measure)	Method of data collection and source (Where and how to find it)	When to be…	
				Collected	Reported
			prioritisation”		
Applications<br>COU of existing TCPs	Has a change of use application process and documentation been established and is it being utilised?<br>Are applicants happy with the process?	Number of applications received	Audit of TCP register	Monthly	Annually
		Number of applications completed correctly at first submission	Audit of TCP register	Monthly	Annually
		Applicant satisfaction with COU application process	Audit application feedback forms	Monthly	Annually
Decision-making<br>New TCPs	Have processes and documentation for decision-making been established and are they being utilised?	Appropriate representation at TCPC meetings to discuss applications	Audit of minutes for attendance by applicant/HOD/Program Director	Monthly	Annually
		Number of applications that the TCPC utilised the decision summary for	Audit of application folders	Monthly	Annually
		Number of decision summaries published on the website	Cross check applications with webpage	Monthly	Annually
		Number of applicants that complied with the conditions of approval and were received by the due date	Audit of TCP register	Monthly	Annually
		Number of outcome letters for approval for provisional use sent	Audit of TCP register	Monthly	Annually
		Number of applicants that appealed to the Chief Executive re the TCPC decision	Audit of TCP register	Monthly	Annually
Decision-making<br>Review of approved TCPs	Have processes and documentation for decision-making for reviewed TCPs been established and are they being utilised?	Number of reviews that the TCPC utilised the decision summary for	Audit of application folders	Monthly	Annually
		Number of decision summaries published on the website	Cross check applications with webpage	Monthly	Annually
		Number of TCPs withdrawn after review	Audit of TCP register	Monthly	Annually
		Number of decisions made that were consistent with the evidence	Review of the evidence	Monthly	Annually
Monitoring and reporting for newly introduced TCPs	Have monitoring and reporting processes been established and are they being utilised?	Number of reports<br>received<br>received by due date<br>received late	Audit of TCP register	Biannually	Annually
		Number of applicants who used TCPC outcome spreadsheet	Audit of TCP register	Biannually	Annually
		Number of applicants who used their own outcome data collection tool	Audit of TCP register	Biannually	Annually
		Number of reporting templates	Audit of TCP register	Biannually	Annually

Components	Key evaluation questions	Success measures/indicators (What to measure)	Method of data collection and source (Where and how to find it)	When to be…	
				Collected	Reported
		completed correctly			
	Are applicants happy with the process?	Applicant satisfaction with reporting processes	Audit application feedback forms	Biannually	Annually
	Were patient outcomes as expected?	Number of procedures performed<br>Referred versus treated<br>Expected versus actual<br>Deaths<br>Other adverse events	Comparison between original applications and progress report data	Biannually	Biannually
Resources	Has a support system and resource documents been developed and are these being utilised?	Number of applicants that utilised patient information template	Audit of application documents	Biannually	Annually
		Applicant satisfaction with quality and accessibility of resources	Audit application feedback forms	Monthly	Annually
		Feedback from resource providers	Via formal meeting or request feedback via email	Biannually	Annually
		Number of requests for use of resources/expertise externally	Audit of requests	Annually	Annually
