# Supplementary material for: Development, implementation and evaluation of an evidence-based program for introduction of new health technologies and clinical practices in a local healthcare setting
Source: BMC Health Serv Res. 2015 Dec 28;15:575. doi: 10.1186/s12913-015-1178-4 (PMC4692064; doi:10.1186/s12913-015-1178-4)
Supplement: Additional file 2: — Best Practice Guide. (PDF 423 kb) [file 12913_2015_1178_MOESM2_ESM.pdf]

Additional file 2: Best Practice Guide

PRINCIPLES	Literature citations		Identified by Monash Health staff			Monash Health performance	
Dev = Development phase, Imp = Implementation phase, Rev = Revision phase, N/A = Not applicable (identified after Development phase)<br>*✓= Principles incorporated in Monash Health processes, + = Principles identified by Monash Health staff*	Dev N=5	Rev N=16	Dev	Imp	Rev	Dev	Rev
**Governance**							
A Technology/Clinical Practice Committee (TCPC) is established	5	16				✓	✓
There is a range of clinical disciplines represented on the TCPC	4	8				✓	✓
Risk management procedures are in place	3	13					✓
Any conflicts of interests are disclosed	3	10					✓
The TCPC operates within a reporting structure to ensure corporate and clinical governance	2	11				✓	✓
There is a consumer representative on the TCPC	1	5				✓	✓
TCPC meetings are held at regular intervals	1	7					✓
A clear process for appeal is in place		9	+				✓
Review of complex applications is facilitated by communication with other relevant committees (eg Research Ethics, Clinical Ethics, etc)		3	+				✓
Sufficient staffing levels are provided to administer the Technology/Clinical Practice Program		2	+				✓
Definition of new technologies and clinical practices (TCPs) is provided		11		+		N/A	✓
Organisational policy on 'Introduction of new TCPs' is available		11		+		N/A	✓
Additional members can be co-opted to the TCPC for expertise, independence, etc as required		7		+		N/A	✓
Responsibility for management, administration and review of policy on 'Introduction of new TCPs' is stated		6		+		N/A	✓
Organisational policy states that new TCPs cannot be introduced without approval		6		+		N/A	✓
Meeting dates are scheduled in advance and published		3		+		N/A	✓
Compliance with organisational policy on 'Introduction of new TCPs' is mandatory		2		+		N/A	✓
Advice on whether TCP falls within the scope of 'Introduction of new TCPs' policy is provided		2		+		N/A	✓
Manufacturers, vendors and suppliers are not permitted to submit a TCP application		1		+		N/A	✓
There is expertise in EBP, Corporate Operations, Finance, Infrastructure and Equipment needs, Ethics and Legal issues on the TCPC				+		N/A	✓
External/independent expertise is available for advice to the decision-making committee		8		+		N/A	✓
Sufficient staffing levels are provided for expert and independent input to application process		2			+	N/A	✓
TCPC members have sufficient levels of seniority, credibility and influence to make and implement appropriate and acceptable decisions		2			+	N/A	
**Decision-Making**							
Evidence concerning a new TCP is robust and reliable	5	16					✓
Ethics procedures are in place to protect patients, clinicians and the community	4	15				✓	✓
Patient information and informed consent procedures are established	3	10	+			✓	✓
There are established criteria for assessment of applications to introduce a new TCP	1	11	+			✓	✓
Safety of a new TCP is established		16	+				✓
Legislative requirements are met		10	+				✓
Issues of access and equity are considered		8	+				✓
Recommendations for introduction have clearly noted conditions eg audit, clinical trial, operational restrictions		8	+				✓

PRINCIPLES	Literature citations		Identified by Monash Health staff			Monash Health performance	
Dev = Development phase, Imp = Implementation phase, Rev = Revision phase, N/A = Not applicable (identified after Development phase)<br>*✓ = Principles incorporated in Monash Health processes, + = Principles identified by Monash Health staff*	Dev N=5	Rev N=16	Dev	Imp	Rev	Dev	Rev
Decisions of the committee are published to ensure transparency and accountability		6	+				✓
A clear process for handling urgent introductions of new TCPs to minimise patient harm is in place		4	+				✓
Approval required for change of use of current TCP eg new indication/population/practitioners or modification to equipment/technique		4	+				✓
Newly introduced TCPs are reassessed at the end of a predetermined monitoring period to reclassify as 'standard care'		1	+				✓
The new TCP has been evaluated or used elsewhere		15		+		N/A	✓
Approval required, in addition to Human Research Ethics Committee authorisation, for introduction of a new TCP in a research project		5		+		N/A	✓
High level evidence is required if the application is based on a case for increased effectiveness (eg Systematic Review, RCT)		4		+		N/A	✓
Any available evidence of cost-effectiveness of a new TCP is provided		8		+		N/A	✓
Regulatory approval is required		8		+		N/A	✓
Standards of practice set by professional associations are met		6		+		N/A	✓
Health economics approach is included eg considering opportunity costs, indirect and direct costs and benefits, etc		11				N/A	
**Application Process**							
Appropriate clinical and physical infrastructure/facilities exist to support the introduction of new TCPs	5	16					✓
Appropriate, credentialed and trained staff are in place for the introduction of new TCPs	5	16				✓	✓
The projected financial costs for proposed TCP are estimated	5	9					✓
Evidence-based practice informs conditions and logistics for introduction	4	14					
Information about the TCP is disseminated and advice provided	4	4				✓	✓
Completed applications are forwarded to the Chair of the TCPC or other nominated delegate	2	10				✓	✓
A register of applications and approved TCPs is maintained	2	6				✓	✓
Applications require endorsement from Departmental Head and Program/Division Director	2	12				✓	✓
Appropriate training is provided to all staff so that each TCP is performed (and all equipment is handled) safely	2	14				✓	✓
Clinical and financial effects of each TCP are considered at all levels and in all departments	1	10					✓
Application forms contain questions on all decision-making criteria		8	+				✓
Application forms are not accepted if they are incomplete or if there is insufficient detail for decision-making		3	+				✓
Opportunities for disinvestment of current practices following introduction of new TCP are identified		2	+				✓
Application forms are completed and submitted electronically		1	+				✓
Application submission deadlines allow sufficient time for adequate review by committee members		1	+				✓
Application forms for introduction of new TCPs are provided		14		+		N/A	✓
Clinical need for TCP is addressed		14		+		N/A	✓
The existing financial costs for current practice are estimated		7		+		N/A	✓
Description of clinical governance arrangements and processes that oversee implementation of new TCP is provided		6		+		N/A	✓
A detailed implementation plan and timeframe for introducing new TCP in a health service is provided		4		+		N/A	✓
Applicants are informed in writing of the outcome and recommendations of the committee		4		+		N/A	✓
Details of any assessment of the TCP by national health policy agency are provided (eg Australian Medical Services Advisory Committee)		3		+		N/A	✓

PRINCIPLES	Literature citations		Identified by Monash Health staff			Monash Health performance	
Dev = Development phase, Imp = Implementation phase, Rev = Revision phase, N/A = Not applicable (identified after Development phase) *✓= Principles incorporated in Monash Health processes, + = Principles identified by Monash Health staff*	Dev N=5	Rev N=16	Dev	Imp	Rev	Dev	Rev
Procurement staff are aware of need for TCPC approval before purchases are made		3		+		N/A	✓
Applicants, Department Heads and Program/Division Directors respond to queries raised by TCPC (at meeting or by correspondence)		2		+		N/A	✓
Application forms meet the requirements of regional, state and/or national applications for introduction or funding of new TCPs		2		+		N/A	✓
Application guidelines are available		1		+		N/A	✓
Contact details of external referees with experience in the new TCP are provided		1		+		N/A	✓
Successful applicants are informed in writing of the conditions of implementation and reporting requirements		1		+		N/A	✓
Application forms are provided for two year review, change of use of existing TCP and use of new TCP in research				+		N/A	✓
Objective and suitable expertise is used for issues relating to resources (financial, space, equipment, staff)		7			+	N/A	
Objective and suitable expertise is used to identify the best available evidence from the research literature		3			+	N/A	
Evidence provided is based on a rigorous systematic review of the research literature		3			+	N/A	
Relevant existing policies/procedures in the organisation are considered when introducing new TCPs		10				N/A	
An evaluation protocol for the new TCP is provided (including all relevant indicators and defined time points)		6				N/A	
Alternatives to the new TCP are listed and compared		5				N/A	
Applicant discusses 'Impact of Not Proceeding' considering patient safety, government policy, financial implications, service delivery, etc		2				N/A	
Issue of credentialing in emergency situations is addressed		2				N/A	
Applicant completes Workforce Impact Statement considering current/future shortages, education and training, industrial issues, etc		1				N/A	
Include manufacturer, vendor, supplier information for purposes of describing new TCP, legal/contractual issues, etc		1				N/A	
Choice between new, existing and other alternatives should be made based on direct comparisons ie 'head to head'					+	N/A	
**Monitoring and Reporting**							
Specified outcomes for each approved TCP are monitored and reviewed	5	11	+				✓
Any adverse event occurring with a new TCP is notified to the TCPC	3	6				✓	✓
Determine processes/requirements for monitoring and reviewing existing TCP	2	11	+				✓
Regular reports are submitted to the state health department detailing applications, approvals, monitoring of new TCPs	2	5					✓
Regular reports (at an agreed/ defined interval) are submitted to the health service executive	2	4					✓
Local consumer health councils and networks will be informed of applications and of their outcomes	1	1					✓
Prompts are sent to applicants prior to the due date for reporting of outcomes				+		N/A	✓
Reminders are sent if outcomes reports are not received by the due date				+		N/A	✓
Permission to practice using the new TCP is withdrawn if outcomes reports are not received after a specified number of reminders				+		N/A	✓
Applicants are required to notify TCPC that all specified conditions have been met prior to implementation of a new TCP				+		N/A	✓
Outcomes are collated in a database/register		7		+		N/A	✓
Applicants are required to report specified outcomes to TCPC at agreed defined interval (eg 6 monthly for 2 years)		5		+		N/A	✓
Any adverse event occurring with a new TCP is notified to the relevant authority (if regulated eg Therapeutic Goods Administration)		4		+		N/A	✓
If the TCP carries risk of adverse events, criteria for reviewing outcomes are established prior to procedures being performed		4				N/A	
Ethics approval as a Quality Assurance activity is obtained prior to data collection					+	N/A	✓

PRINCIPLES	Literature citations		Identified by Monash Health staff			Monash Health performance	
Dev = Development phase, Imp = Implementation phase, Rev = Revision phase, N/A = Not applicable (identified after Development phase)<br>*✓ = Principles incorporated in Monash Health processes, + = Principles identified by Monash Health staff*	Dev<br>N=5	Rev<br>N=16	Dev	Imp	Rev	Dev	Rev
**Resources**							
Expertise in coding, data analysis, evidence review, finance and credentialing is provided		5	+				✓
Applicants are directed to guidance on finding the evidence of effectiveness of TCPs to support the application		4	+				✓
Template for Patient information brochure is provided		2	+				✓
Templates for data collection tools and reporting proformas are provided (for therapeutic interventions)		1	+				✓
Website housing documents and resources is developed and maintained		1	+				✓
List of organisations that can provide evidence for the effectiveness of TCPs and in some cases service configuration are provided		8		+		N/A	✓
Assessment guidelines are provided ie how evidence submitted will be assessed		2		+		N/A	✓
Templates for appraising, summarising and presenting the evidence are provided		2		+		N/A	✓
Business case guidelines are available		3				N/A	
Formal Risk Assessment Tool is provided		2				N/A	
Templates for data collection tools and reporting proformas are provided (for diagnostic tests)		1			+	N/A	
Business case template is provided		1				N/A	
Life-cycle costing template is provided		1				N/A	
Guidance on approach for conducting economic evaluation is available		1				N/A	
Expertise in contract negotiation and equipment maintenance requirements is provided					+	N/A	
**Administration**							
Staff with appropriate expertise and sufficient time are designated to manage the TCP program (eg Executive Officer, Administrator, etc)		7		+		N/A	✓
Systems, processes and resources are developed, implemented, maintained, evaluated and improved		4		+		N/A	✓
Processes are facilitated through checklists, timelines, diarising of due dates, electronic reminders, etc				+		N/A	✓
**Evaluation and Quality Improvement**							
Data collection methods are established		4		+		N/A	✓
Evaluation findings are published		3		+		N/A	✓
A framework and plan for evaluation of the TCP program is developed and implemented		2		+		N/A	✓
Feedback is sought from decision-makers and administrators on systems and processes		1		+		N/A	✓
Application forms have feedback page to capture comments from users				+		N/A	✓
Feedback is sought from applicants on Content, Wording and Format of Application forms, Assistance provided and Resources available				+		N/A	✓
Improvements to systems, processes, documents and resources are implemented based on evaluation findings				+		N/A	✓
**TOTAL** **122**	**27**	**109**	**25**	**51**	**9**	**14**	**100**
