# Supplementary material for: Development, implementation and evaluation of an evidence-based program for introduction of new health technologies and clinical practices in a local healthcare setting
Source: BMC Health Serv Res. 2015 Dec 28;15:575. doi: 10.1186/s12913-015-1178-4 (PMC4692064; doi:10.1186/s12913-015-1178-4)
Supplement: Additional file 3: — Barriers and enablers and strategies to address them. (PDF 424 kb) [file 12913_2015_1178_MOESM3_ESM.pdf]

Additional file 3: Barriers and enablers and strategies to address them

BARRIERS	STRATEGIES
**Economic and political context (financial arrangements, regulations, policies)**	
The state Department of Health has an annual funding round for new high cost TCPs. Stakeholders may be frustrated, confused or waste time with duplication if the TCPC documents have different content and format to the Department of Health.	Make TCPC application form meet Department of Health requirements for funding of high cost TCPs
The TCPC will be guided by decisions of the Australian Medical Services Advisory Committee (MSAC). All stakeholders will waste time and be frustrated if applicants are unaware of national policy and complete the application process unnecessarily.	Add a step in the process that requires the applicant to check for MSAC reviews on the new TCP. If the TCP is recommended by MSAC the applicant does not need to provide detailed evidence from research. If the TCP is not recommended, the application should not be continued.
Lack of finances to buy technologies	Addressed by explicit criteria to assess cost and affordability and transparency of publishing decisions
**Organisational context (organisation of care processes, staff, capacities, resources, structures)**	
TCPC may not be held in sufficiently high regard for applicants to respect and abide by processes	Introduce mandatory policy that all new TCPs must go through new authorisation process
	Raise profile and influence of TCPC by upgrading committee relationship structure so that TCPC reports to the Executive Management Team
Decision-maker's lack of time to read extensive documentation prior to meeting due to busy workloads	Secretariat to provide all documents at least one week prior to meeting
	Secretariat to do all the 'work' of the committee (eg preparation, following up actions, etc)
Applicant's lack of time to complete application form for introduction of new TCP due to busy workloads	Make application form as user-friendly as possible eg use 'tick boxes' as alternatives to free text
	CCE staff provide help to find evidence eg assistance with searches
	TCPC Secretariat to provide assistance with document completion in the initial phase so that applicants can see what is required of them
Applicant's lack of time to complete application form for Change of use and Two year review due to busy workloads	Use 'tick box' format throughout
	Accept documented declaration by applicant of endorsement by Program Director, Executive Director and Business Manager without actual signatures required
Administrator's lack of time to manage the proposed processes of the seven new components due to no time allocation for TCPC processes	Allocate resources by diverting CCE staff time from other areas to TCPC. CCE Director as Executive Officer (1-2 hours/week) and a CCE Project Officer as Administrator (1-2 days/week).
Specialist resource staff (eg coders, data analysts) lack of time to provide adequate information for decision-making due to applicants leaving requests to the last minute	Introduce time limits eg Applicants must contact coders at least two days and data analysts at least two weeks before information is required
	Include instructions in the application form regarding deadlines for support services
Six-monthly reports to monitor new TCPs based on the date of introduction are inconvenient, confusing and create extra work due to multiple deadlines	Change all reporting periods to single format (Jan-Jun and Jul-Dec). Applicants may report for part of the first and last six-monthly period if they introduce a new TCP in that time frame
Lack of central source of information for TCPC processes	Create and promote a webpage to house all information, documents and resources
Purchases will continue to be made without appropriate authorisation	Inform Procurement Department of requirements and involve Procurement Director in program
Decision-makers cannot attend meetings due to other commitments	Set meeting dates in advance to maximise attendance and allow appropriate representation
	Encourage those unable to attend to provide feedback regarding agenda items at the time of apology
**Social context (opinion of colleagues, culture of the network, collaboration, leadership)**	
CCE Project team has no role in the process therefore limited influence	Make Project team responsible for the process. CCE staff become the TCPC Secretariat
Potential duplication of activities between the project team and TCPC administrators	
Decision-makers under pressure to approve applications, particularly if new TCP in use elsewhere eg overseas, in private hospitals	Addressed by program elements to achieve transparency, accountability and EB decisions eg Explicit criteria, published Decision Summaries, etc
	Seek support for these principles from Executive Management Team and health service Board
Applicants perceive that health service management priorities are about saving money	Promote decision-making principles, stress safety and effectiveness, better patient outcomes, etc

Power and budget struggles affecting perceptions and acceptance of decisions	Addressed by program elements to achieve transparency, accountability and EB decisions eg Explicit criteria, published Decision Summaries, etc
**Patient (knowledge, skills, attitude, compliance)**	
Lack of consumer input if single consumer representative unable to attend meeting	Increase to two consumer representatives
Evidence that having less than two consumers on committees is not best practice	
Applicants do not know how to write high quality patient information (usually too much, too technical and omits information the patient wants to know)	Include input from consumer representatives on draft patient information materials
	Develop template for patient information
Consumer representatives will incur costs to print out meeting papers	Send hardcopy of papers in Express Post at same time as electronic circulation
**Individual professional (awareness, knowledge, attitude, motivation to change, behavioural routines)**	
Applicant's lack of awareness of process and requirements	Introduce mandatory policy that all new TCPs must go through new authorisation process
	Develop protocol outlining steps in new processes
	Explain reasons for new processes in communication strategy
	Notify all staff via organisational newsletters
	Send bulletins to likely applicants via All Managers, Dept Head and Senior Medical Staff email lists
	Hold face-to-face meetings with Medical Program Directors
	Communicate with Managers of Operating Suites and Procedural facilities
	Inform Procurement Department of requirements and involve Procurement Director in program
	Create and promote a webpage to house all information, documents and resources
	Require that use of new TCP introduced without authorisation is ceased until process is complete
Applicant's lack of knowledge regarding what should be considered a 'new TCP' or 'Change of use' and when applications are required	Provide clear definitions for 'new TCP' and 'Change of use' and instructions for when applications are required
Applicant's lack of autonomy: unwillingness to submit control to application process or to wait until process complete before commencing	Same as lack of awareness (above)
Applicant's belief in benefit of TCP: use new TCP without authorisation to do what they think is best for their patients	Same as lack of awareness (above)
Applicants forget to apply	Same as lack of awareness (above)
Applicant's animosity towards 'red tape'	Same as lack of awareness (above)
	Remove any unnecessary 'red tape'
	TCPC Secretariat to be welcoming, respond to enquiries, provide information and assistance, etc
Applicants do not usually have the appropriate skills to provide the level of detail and quality of information required for decision-making	Provide assistance from relevant experts within the organization eg CCE (evidence), Health Information Services (coding), Clinical Information Management (health service utilisation data), Medical Support Unit (credentialing) and Finance Department (business case).
Applicants do not usually have the appropriate skills in systematic review methods and are often not familiar with the sources of high quality evidence	Develop step-by-step 'Guide to Finding the Evidence' that follows the sequence of questions on the application form.
Applicants continue to provide low level or non-research evidence, or do not use a systematic approach therefore do not provide the best available evidence)	Be explicit about requirement for high level evidence, appropriate evidence re safety, etc
	Provide tools to identify best available evidence and templates to document it
Applicant's frustration with lack of timeliness or relevance of research	Explain that high level high quality evidence is required to introduce change across the organisation
Applicant's frustration with poor quality of research	Explain that high level high quality evidence is required to introduce change across the organisation
Applicants do not monitor and/or report outcomes	Provide prompts one month before deadline
	Issue monthly reminders after deadline
	Withdraw permission to use TCP if no response to second reminder
Applicant's poor handwriting, application difficult to read	Require electronic submission of documents

Applicant's lack of experience in word processing (some senior medical staff had never created an electronic document before)	TCPC Secretariat to provide assistance with document completion in the initial phase so that applicants can see what is required of them
	TCPC Secretariat to help Applicant's Personal/Executive Assistants understand the requirements
Sections of document incomplete or inadequate detail provided	Provide alternatives with 'tick boxes' where appropriate
	TCPC Secretariat to provide assistance when the problem is due to lack of technical expertise
	Require application two weeks before meeting – one for Secretariat to check and one for TCPC to read
	Return document to applicant for completion
Many applicants do not know how to collect data, which data collection tools to use, etc therefore quality may be poor and collation very time-consuming	Create generic data collection tool
Some applicants are very experienced in collecting data and may even be collecting standardised data sets for national or international registries so do not want to duplicate data collection by using generic TCPC tool in addition to their own	Allow generic tool to be optional if applicants already have well developed audit methods
**Innovation (advantages in practice, feasibility, credibility, accessibility, attractiveness)**	
New processes may lack credibility as there is no clear evidence or recognised experts to determine process for introduction of new TCPs if applicants do not consider the national, state and professional bodies who produced the guidance to be credible organisations	Promote decision-making principles, stress safety and effectiveness, better patient outcomes, etc
	Explain role of local consultation in development process
	Explain role of ongoing feedback to allow local needs to influence program
New process is highly complex and requires time, skills and expertise	Same as lack of credibility (above)
	Make processes and documents as simple and user-friendly as possible
	Seek ongoing feedback and refine processes and documents based on feedback
Application form detailed, complicated and probably not attractive to applicants	Same as lack of credibility (above) and high complexity (above)
New process is significantly different from status quo	Same as lack of credibility (above) and high complexity (above)
Applicants may not consider the new program to have any advantages over status quo	Promote decision-making principles, stress safety and effectiveness, better patient outcomes, etc
Applicants have difficulty accessing documents	Create and promote a webpage to house all information, documents and resources

ENABLERS	STRATEGIES
New TCPC processes are a high priority for the organisation	Raise profile and influence of TCPC by upgrading committee relationship structure so that TCPC reports to the Executive Management Team
The organisation is committed to the new TCPC processes	Introduce mandatory policy that all new TCPs must go through new authorisation process
Funding has been provided to establish the new program	Use rigorous methods to develop, implement and evaluate the new program
CCE has high level skills in Evidence Based Practice	Make Project team responsible for the process. CCE staff become the TCPC Secretariat
The decision-makers and project team are willing to change the system based on feedback	Make feedback process known, act upon it, advertise that changes are based on feedback received
improving patient outcomes is known to be a motivator for clinical staff	Promote and explain how processes enhance safe and effective care
All clinical Program/Division Directors are supportive	Use Program/Division Directors to communicate within their programs/divisions
Chair of TCPC highly regarded by applicants and influential within the organisation	TCPC Chair to correspond (in person or in writing) when seeking influence for change or to communicate with applicants who are not following the process
TCPC decision-makers are committed to evidence-based decision-making	Build in rigorous and explicit methods of evidence based practice
Ethics approval processes are well established and accepted in the organisation	Cite ethics process as example of similar system that is both rigorous and familiar to stakeholders
	Addressed by program elements to streamline processes between committees
